# CAR-engineered cytolytic Tregs reverse pulmonary fibrosis and remodel the fibrotic niche with limited CRS

**DOI:** 10.1172/jci.insight.182050

**Published:** 2025-07-08

**Authors:** Yun-Han Jiang, Meng Zhou, Meng-Di Cheng, Sai Chen, Ying-Qiang Guo

**Affiliations:** 1 Department of Cardiovascular Surgery,; 2 Institute of Cardiovascular Surgery,; 3 Laboratory of Human Diseases and Immunotherapies, and; 4 Institute of Immunology and Inflammation, Frontiers Science Center for Disease-related Molecular Network, West China Hospital, Sichuan University, Chengdu, China.

**Keywords:** Immunology, Pulmonology, Therapeutics, Cytokines, Fibrosis, T cells

## Abstract

Idiopathic pulmonary fibrosis (IPF) is a severe, diffuse, progressive, and fibrosing interstitial disease leading to respiratory failure and death in the absence of organ transplantation. Substantial evidence has confirmed the pivotal role of fibroblasts in the progression of IPF, yet effective therapeutic options are scarce. Single-cell transcriptomics profiling revealed that among the diverse fibroblast subsets, FAP1^+^ alveolar fibroblasts (AFs) were pivotal for the progression of IPF. On the basis of these findings, we developed FAP1-targeting chimeric antigen receptor cytotoxic effector regulatory T cells (CAR-cTregs), which leveraged the targeted killing advantage of the currently trending CAR-based immunotherapy for tumors and incorporated the immunosuppressive functions of Tregs to mitigate the inflammation caused by both the disease itself and CAR-T cell infusion. Accordingly, CAR-cTregs were constructed to effectively eliminate FAP1^+^ fibroblasts in vitro. This cytotoxic effect could be abrogated by inhibitors of the granzyme B/perforin pathway. In the bleomycin-induced PF model, CAR-cTregs were found to reverse fibrosis characterized by diminished recruitment of fibrocytes and improved remodeling of epithelial cells. Together, our results demonstrate that CAR-cTregs can serve as a promising therapeutic option for IPF and provide an alternative strategy for treating multiple chronic inflammatory diseases by inducing both cytotoxicity and immunosuppression.

## Abstract 2

FAP1+ alveolar fibroblasts are pivotal for the progression of Idiopathic pulmonary fibrosis. FAP1-targeting CAR cytotoxic effector Tregs reverse pulmonary fibrosis with fibrotic niche remodeling.

## Introduction

Idiopathic pulmonary fibrosis (IPF) is a severe, diffuse parenchymal lung disease characterized by the accumulation of fibroblasts (Fibs) and increased extracellular matrix (ECM) deposition. The mean survival time of patients with IPF is 2–5 years after diagnosis (1–3), and IPF ultimately leads to respiratory failure and death in the absence of organ transplantation. Although antifibrotic agents, such as pirfenidone and nintedanib, inhibit the decrease in forced vital capacity and improve survival, these agents cannot halt disease progression and have adverse effects, including gastrointestinal disorders, skin-related problems, and liver damage (4). Thus, more effective therapeutic approaches for IPF need to be further explored.

Substantial effort has been made to study the role and origin of Fib subsets, as they are promising targets for antifibrotic therapy. At the tissue level, IPF is defined by a fibroblastic focus with an immature hyaluronic acid–rich matrix underneath the epithelial layer, loss of alveolar type 1 cell differentiation, and increased α-smooth muscle actin^+^ (αSMA^+^) myofibroblasts (5). However, owing to the unresolved delineation of the precise roles and operational mechanisms of the diverse Fib subpopulations implicated in the pathogenesis of IPF, developing targeted therapeutic strategies remains challenging.

The genetic redirection of conventional T cells (Tconvs) with chimeric antigen receptors (CARs) has enabled polyclonal peripheral blood T cells to be redirected to a specific tumor-associated antigen (6, 7). These CAR-T cells have achieved extraordinary success in cancer treatment and are approved for the treatment of patients with certain types of B cell lymphoma (8, 9). In our preceding studies, CAR-T cell infusion significantly ameliorated the stenosis of transplanted venous grafts in a murine model of coronary artery bypass grafting (10). More recently, despite the substantial success of CAR-T cell therapies in a range of nonneoplastic conditions, such as autoimmune diseases and senescence, severe cytokine-release syndrome (CRS) has significantly limited the application of CAR-T cell therapies in nononcological inflammatory diseases.

Tregs have been shown to play protective roles in acute and chronic inflammation (11–13) and decrease CAR-T cell–induced CRS (14). Emerging evidence suggests that specific subsets of cytotoxic effector Tregs (cTregs) possess both cytotoxic functions and immunosuppressive capabilities (15). Adoptive transfer of cTregs suppresses graft-versus-host disease (GVHD) while effectively preserving graft-versus-leukemia (GVL) activity (16, 17).

In the present study, we identified alveolar Fibs (AFs) expressing fibroblast activation protein 1^+^ (FAP1^+^) as a key subset involved in IPF progression by mining single-cell RNA sequencing (scRNA-seq) data. CAR-cTregs were constructed to effectively eliminate FAP1^+^ Fibs (aFibs) via the granzyme B (GZMB)/perforin (PFP) pathway and reverse bleomycin-induced (BLM-induced) PF, which is characterized by diminished recruitment of fibrocytes and improved remodeling of epithelial cells. Leveraging the targeted cytotoxicity of CAR-T cells and the CRS-suppressive effects of Tregs, CAR-cTreg represents a promising therapeutic modality for the treatment of chronic inflammatory diseases, particularly PF.

## Results

### FAP1^+^ AFs are the main collagen producers involved in IPF.

**Figure 1:**
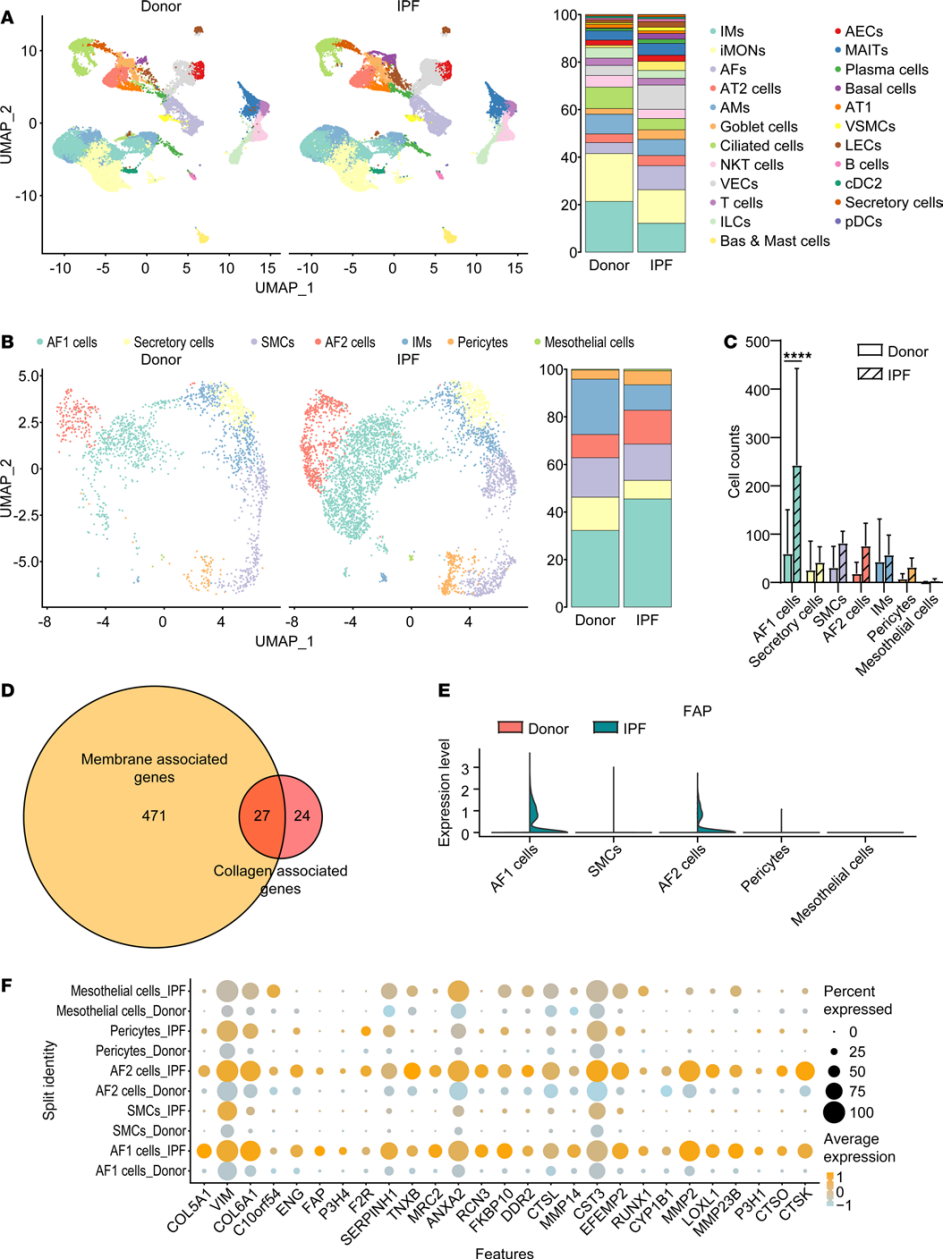
FAP1^+^ AF cells are the main collagen producers in IPF. (**A**) UMAP of scRNA-seq data from healthy donors (HDs, *n* = 10) and patients with IPF (*n* = 8). cDCs, conventional dendritic cells; pDCs, plasmacytoid dendritic cells; IMs, interstitial macrophages; iMONs, inflammatory monocytes; AMs, alveolar macrophages; VECs, venous epithelial cells; ILCs, innate lymphoid cells; MAITs, mucosal-associated invariant T cells; LECs, lymphatic endothelial cells. (**B**) UMAP analysis of mesenchymal cells including alveolar fibroblasts (AFs) and vascular smooth muscle cells (VSMCs) in HDs (*n* = 10) and IPF patients (*n* = 8). (**C**) Absolute counts of the 5 stromal subsets in HDs (*n* **=** 10) and IPF patients (*n* **=** 8). (**D**) Venn diagram of 498 membrane-associated genes and 51 collagen-associated genes. (**E**) Violin plot of FAP expression is shown in 5 stromal subsets, including AFs (AF1 and AF2), mesothelial cells, pericytes, and smooth muscle cells (SMCs). (**F**) The expression of 27 selected genes is shown in AF1, AF2, mesothelial cells, pericytes, and SMCs. ****P < 0.0001 by 2-way ANOVA with Tukey’s post hoc test. The data in **C** are presented as the mean ± SD.

To investigate the cell subset responsible for PF, we analyzed the single-cell transcriptomes of patients with IPF and healthy donors (NCBI GEO GSE128033). The basic characteristics are listed in Supplemental Table 1; supplemental material available online with this article; https://doi.org/10.1172/jci.insight.182050DS1 The single-cell profiles clustered into 23 subsets (Figure 1A), which were labeled according to the cell markers of lung CellCards (18). The robustness and reproducibility of the subsets were confirmed in all the samples, which were proportionally distributed across patients (Supplemental Figure 1, A and B) and an alternative clustering algorithm was used (Supplemental Figure 15, A–C). Among the 23 subsets in both healthy donors and IPF patients, venous endothelial cells exhibited increases in both the relative proportion and absolute count within the IPF cohort (Supplemental Figure 1, C and D). The proportion of AFs was significantly elevated in IPF patients, whereas the absolute number of AFs was nearly significantly increased (*P* = 0.0563) (Supplemental Figure 1, C and D), which is in line with previous findings (19). Furthermore, the mesenchymal cells included 5 stromal subsets (Figure 1B), with a significant increase in the proportion of AF1 cells observed in patients with IPF (Figure 1C and Supplemental Figure 1E). Previous studies have shown that AF1 and AF2 are the main subsets involved in PF (19). Similarly, we observed that AF1 and AF2 cells collectively accounted for more than 50% of the mesenchymal cells in patients with IPF (Supplemental Figure 1E).

To further clarify the role of AFs in IPF, we assessed the activity of fibrosis-related pathways across cellular subsets. We retrieved 17 pathway-related gene sets linked to fibrosis from the biological process category of the Gene Ontology, Kyoto Encyclopedia of Genes and Genomes (KEGG) pathway, and Reactome pathway databases (Supplemental Table 2). These cover multiple stages of fibrosis from initiation to maturation. Our data revealed that AF1 and AF2 cells presented high expression of most genes within these 17 gene sets (Supplemental Figure 19A, Supplemental Figure 21A, Supplemental Figure 23A, Supplemental Figure 26A, Supplemental Figure 27A, Supplemental Figure 33A, Supplemental Figure 35A, Supplemental Figure 37A, Supplemental Figure 39A, Supplemental Figure 41A, Supplemental Figure 43A, Supplemental Figure 45A, Supplemental Figure 47A, Supplemental Figure 50A, Supplemental Figure 51A, Supplemental Figure 53A, Supplemental Figure 55A, Supplemental Figure 58A, and Supplemental Figure 59A). These cells presented significantly increased activity in most fibrosis-related pathways, including “ECM assembly,” “ECM organization,” “regulation of ECM organization,” “positive regulation of ECM organization,” “ECM - basal lamina,” “anchoring fibril formation,” “collagen biosynthesis and modifying enzymes,” “collagen chain trimerization,” “crosslinking of collagen fibrils,” “assembly of collagen fibrils and other multimeric structures,” “collagen formation,” “molecules associated with elastic fibers,” “elastic fiber formation,” and “ECM proteoglycans”. Notably, AF1 cells also presented high module scores in pathways such as “regulation of ECM assembly” and “positive regulation of ECM assembly” (Supplemental Figure 19, B and C, Supplemental Figure 21, B and C, Supplemental Figure 23, B and C, Supplemental Figure 25, A and B, Supplemental Figure 31, A and B, Supplemental Figure 34, B and C, Supplemental Figure 36, B and C, Supplemental Figure 38, B and C, Supplemental Figure 40, B and C, Supplemental Figure 42, B and C, Supplemental Figure 44, B and C, Supplemental Figure 46, B and C, Supplemental Figure 48, A and B, Supplemental Figure 51, B and C, Supplemental Figure 53, B and C, Supplemental Figure 55, A and B, Supplemental Figure 57, B and C). In patients with IPF, AF1 and AF2 cells displayed significantly increased activity in most fibrosis-related pathways, including “ECM assembly,” “ECM organization,” “ECM - basal lamina,” “anchoring fibril formation,” “collagen biosynthesis and modifying enzymes,” “collagen chain trimerization,” “crosslinking of collagen fibrils,” “assembly of collagen fibrils and other multimeric structures,” “collagen formation,” “molecules associated with elastic fibers,” “elastic fiber formation,” “ECM proteoglycans,” and “fibronectin matrix formation” (Supplemental Figure 19, D and E, Supplemental Figure 20A, Supplemental Figure 21, D and E, Supplemental Figure 22A, Supplemental Figure 23, D and E, Supplemental Figure 24A, Supplemental Figure 25, C and D, Supplemental Figure 28A, Supplemental Figure 29A, Supplemental Figure 30A, Supplemental Figure 32, C and D, Supplemental Figure 34A, Supplemental Figure 35, D and E, Supplemental Figure 36A, Supplemental Figure 37, D and E, Supplemental Figure 38A, Supplemental Figure 39, D and E, Supplemental Figure 40A, Supplemental Figure 41, D and E, Supplemental Figure 42A, Supplemental Figure 43, D and E, Supplemental Figure 44A, Supplemental Figure 45, D and E, Supplemental Figure 46A, Supplemental Figure 47, D and E, Supplemental Figure 48A, Supplemental Figure 49, C and D, Supplemental Figure 52A, Supplemental Figure 53, D and E, Supplemental Figure 54A, Supplemental Figure 55, D and E, Supplemental Figure 56A, Supplemental Figure 57, C and D, Supplemental Figure 58B, Supplemental Figure 59, D and E, and Supplemental Figure 60A). These findings indicate that AF1 and AF2 cells are characterized by high expression of genes related to PF and significant activity in fibrosis-related pathways, and that AF cells may be the main collagen-producing cells in IPF.

To identify membrane-associated and collagen-producing–associated target molecules, the differentially expressed genes (DEGs) of AF1 cells and AF2 cells were further analyzed in both healthy donors and patients with IPF (Supplemental Table 3). Among the 866 DEGs, 498 membrane-associated genes and 51 collagen-associated genes were found, 27 of which were associated with both the membrane and collagen (Figure 1D). The expression of 27 genes in each subset was further investigated. Among the 27 eligible genes, FAP1 was specifically expressed in AF1 and AF2 cells (Figure 1F, Supplemental Figure 1F, and Supplemental Figure 2). Moreover, FAP1 was barely expressed in any of the other subsets except AF1 and AF2 cells, and it was highly expressed specifically in the AF1 and AF2 cells of patients with IPF (Figure 1E, Supplemental Figure 1G, and Supplemental Figure 2). Immunofluorescence assays confirmed the significant increase in the number of FAP1^+^ AFs within the context of IPF (Supplemental Figure 3, A and B). Thus, FAP1^+^ AFs may be a potential target of collagen-producing subsets that are responsible for IPF.

### Generation and characterization of FAP1-specific CAR-cTregs.

**Figure 2:**
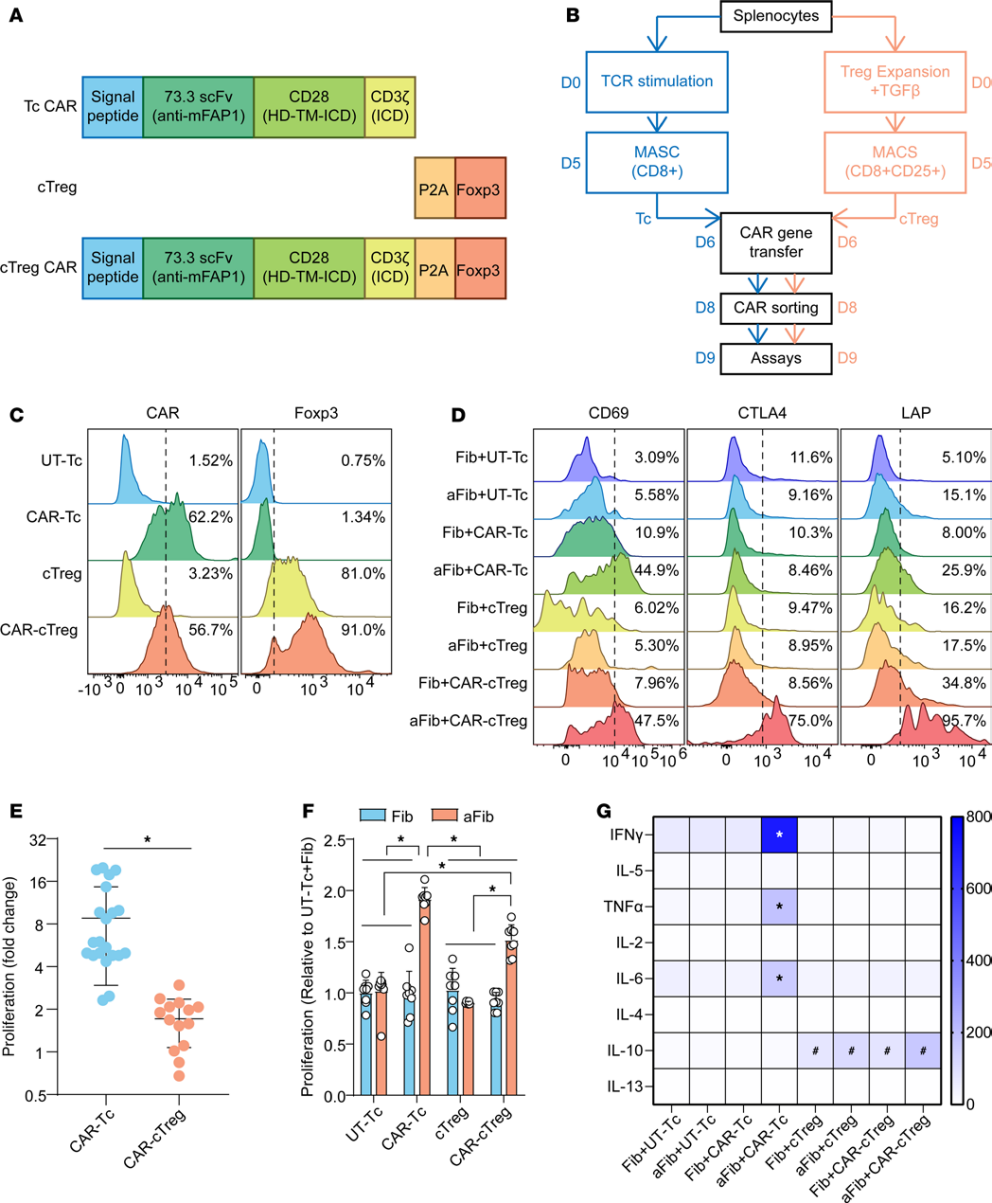
Generation and characterization of FAP1-specific CAR-cTregs. (**A**) Schematic representation of the retroviral vector expressing the FAP1 CAR. HD,; TM, transmembrane domain; ICD, intracellular domain; HD, hinge domain. (**B**) Scheme for the generation and expansion of CAR-cTregs and CAR-Tcs. MACS, magnetic-activated cell sorting. (**C**) The transduction efficiency of the CAR and the expression of FoxP3 in mouse Tregs. (**D**) The expression of T cell activation marker (CD69) and Treg activation–associated markers (CTLA4 and LAP) was measured by flow cytometry after T cells were cocultured with lung Fibs. (**E**) Fold expansion on days 6–8, 3 days after CAR gene transfer (*n* = 21, or 14). (**F**) Proliferation of UT-Tc, CAR-Tc, cTreg, or CAR-cTregs cocultured with aFibs or Fibs. (**G**) The secretion of multiple cytokines in the cocultured media was evaluated by a cytometric bead assay. *P < 0.05 by 2-tailed Student’s *t* test (**E**) or 2-way ANOVA with Tukey’s post hoc test (**F** and **G**). ^#^*P* < 0.05 for comparisons with Fib+UT-Tc, aFib+UT-Tc, Fib+CAR-Tc, or aFib+CAR-Tc. The data are presented as the mean ± SD (**E** and **F**) or the mean (**G**). *n* = 14 or 28 (**E**), *n* = 8 (**F**), *n* = 5 (**G**). CAR-cTreg, chimeric antigen receptor cytotoxic effector Treg cell; cTreg, cytotoxic effector Treg cell; CAR-Tc, chimeric antigen receptor cytotoxic T cell; UT-Tc, untransduced cytotoxic T cell.

To explore whether the expression of FAP1 on the surface of AF1 and AF2 cells could serve as an alternative target for adoptive cell immunotherapy for PF, a FAP1-specific second-generation CAR construct was synthesized. To generate the FAP1 CAR, we cloned a single-chain variable fragment (scFv), clone 2.15. The scFv is followed by transmembrane and cytoplasmic CD28 and intracellular CD3ζ domains capable of activating murine T cells (20). Given the suboptimal therapeutic outcomes of CAR-T cell therapy for PF (21), we opted to utilize cTregs, which possess both cytotoxic functions and immunosuppressive capabilities (15), as the cellular source for CAR-based therapy. To increase the stability of the Treg phenotype, the FoxP3 gene was integrated into the vector and separated from the CAR gene by a P2A peptide sequence (Figure 2A) (22). The murine splenocytes were evenly partitioned into 2 distinct fractions. One fraction was subjected to a 5-day stimulation regimen with anti-CD3/anti-CD28 antibodies in the presence of IL-2 and transforming growth factor β1 (TGF-β1) (Figure 2B). cTregs were enriched and purified via magnetic-activated cell sorting (MACS) on the basis of marker expression in CD8^+^CD25^+^ cells. The other fraction was sorted for CD8^+^ T cells after stimulation with anti-CD3/anti-CD28 antibodies and IL-2, which are considered to be cytotoxic T (Tcs) cells. CAR retroviruses were transduced into these 2 fractions, and then CAR^+^ cells were sorted. The transduction efficiency of the same CAR construct for murine Tcs and cTregs was on average 50%–70% before sorting, and approximately 80%–95% of the cells expressed FoxP3 in the final harvested cell population (Figure 2C). Before CAR sorting, the expansion of CAR-cTregs approximately doubled, a magnitude that was significantly less pronounced than the proliferation of T cells, the latter of which demonstrated an expansion that was approximately 8 times greater (Figure 2E).

Next, to determine the antigen-specific recognition of CAR-cTregs by both activation (Figure 2D and Supplemental Figure 4, A and B) and proliferation (Figure 2F) and the secretion of Th1/2 cytokines (Figure 2G), we utilized aFibs induced by TGF-β1 to emulate the FAP1^+^ AF1 population in IPF. CAR-cTregs expressed significantly greater levels of the activation markers CD69, CTLA4, LAP, and GITR after coculture with aFibs but not after coculture with Fibs (Figure 2D and Supplemental Figure 4A). Phenotypic analysis of lymphocytes revealed that CAR-cTregs robustly differentiated into effector CD44^+^CD62L^–^ Tregs (Supplemental Figure 4B). After stimulation with aFibs, the number of CAR-cTregs increased approximately 1.5-fold, which was slightly lower than that of CAR-Tcs (Figure 2F). CAR-cTregs hardly produced the proinflammatory cytokines IFN-γ, TNF-α, and IL-6 in response to aFibs, but exhibited increased secretion of the antiinflammatory cytokine IL-10, similar to that of cTregs (Figure 2G). Moreover, CAR-cTregs did not express higher levels of the exhaustion markers PD-1 and Tim-3 (Supplemental Figure 4D) or activation-induced cell death (Supplemental Figure 4C) than the other groups did in in vitro target antigen stimulation assays. These results demonstrated that CAR-cTregs were successfully engineered and exhibited the capacity to specifically recognize target aFibs.

### Functional in vitro evaluation of FAP1-specific CAR-cTregs.

**Figure 3:**
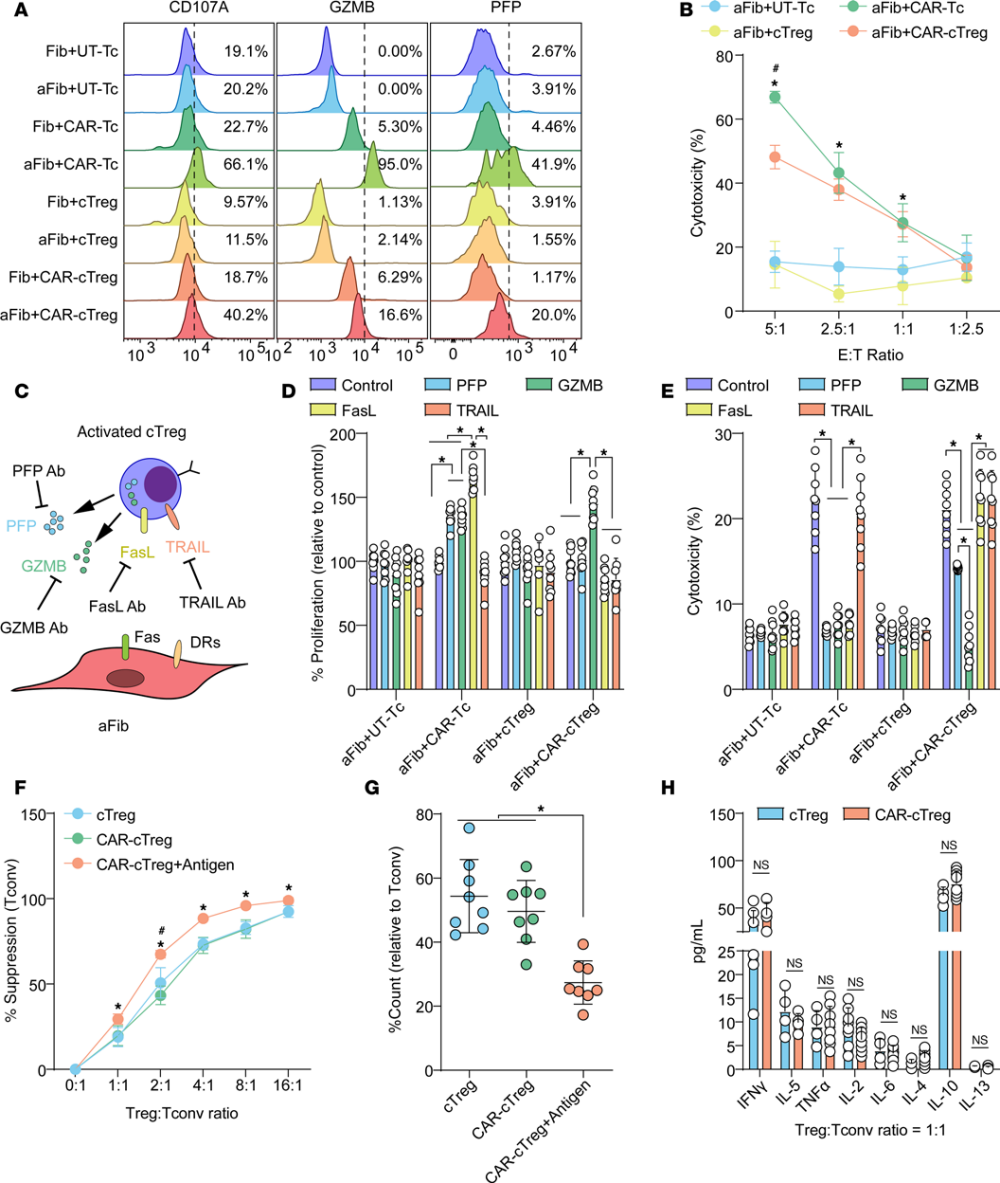
The cytotoxic and immunosuppressive properties of FAP1-specific CAR-cTregs. (**A**) The expression of CD107A, PFP, and GZMB in T cells following coculture with lung Fibs. (**B**) The specific antifibrotic effect on aFibs was measured by LDH activity in culture media at the indicated E:T ratios. (**C**) Schematic diagram of the Fib cytotoxicity assay using neutralizing antibodies against PFP, GZMB, FasL, and TRAIL. DR, death receptor. (**D**) Quantification of Fib proliferation after treatment with neutralizing antibodies. (**E**) Quantification of the results of the Fib cytotoxicity assay using neutralizing antibodies. (**F**) Normalized in vitro suppression of CTV-labeled Tconv proliferation in cells cocultured with cTregs, CAR-cTregs, or FAP1-stimulated CAR-cTregs at the indicated Treg/Tconv ratios. (**G**) Normalized in vitro suppression of the proliferation of CTV-labeled Tconv cells cocultured with cTregs, CAR-cTregs, or FAP1-stimulated CAR-cTregs. (**H**) Detection of IFN-γ, IL-5, TNF-α, IL-2, IL-6, IL-4, IL-10, and IL-13 in the supernatants of untransduced (cTreg) and CAR-cTregs cocultured with Tconvs for 24 hours in vitro. *P* values determined by 2-way ANOVA with Tukey’s post hoc test (**B**, **D**, **E**, **F**, and **H**) or 1-way ANOVA with Tukey’s post hoc test (**G**). (**B**) *P < 0.05 for the comparison to aFib + UT-Tc and aFib + cTreg; ^#^*P* < 0.05 for the comparison between aFib + CAR-Tc and aFib + CAR-cTreg. (**F**) *P < 0.05 for the comparison between CAR-cTreg + antigen and all other groups; ^#^*P* < 0.05 for the comparison between CAR-cTreg and all other groups. The data are presented as the mean ± SD (**B**, and **D**–**H** *n* = 8 each). CAR-cTreg, chimeric antigen receptor cytotoxic effector Treg cell; cTreg, cytotoxic effector Treg cell; CAR-Tc, chimeric antigen receptor cytotoxic T cell; UT-Tc, untransduced cytotoxic T cell.

To evaluate the in vitro antifibrotic effect of CAR-cTregs, the expression profiles of molecules associated with cytotoxicity were examined. Our investigation revealed that the expression levels of CD107a, GZMB, and PFP were significantly increased in the coculture of CAR-cTregs with aFibs, albeit to a lesser extent than in the coculture of CAR-Tcs with aFibs (Figure 3A). Primary lung aFibs were cocultured with untransduced cytotoxic T (UT-Tc) cells, CAR-Tcs, cTregs, or CAR-cTregs at different effector T cell to target cell (E:T) ratios. As shown in Figure 3B and Supplemental Figure 16A, CAR-cTregs effectively and specifically induced aFib apoptosis, although the in vitro antifibrotic efficiency of CAR-cTregs was lower than that of CAR-Tcs. High context analysis revealed that CAR-cTregs can approach aFibs and induce aFib apoptosis (Supplemental Videos 1–4). To further assess the targeting specificity of CAR-cTregs, Fibs overexpressing FAP and those with FAP knockdown were cocultured with UT-Tcs, CAR-Tcs, cTregs, or CAR-cTregs at various E:T ratios. The results demonstrated that CAR-Tcs and CAR-cTregs exhibited E:T ratio–dependent cytotoxic effects only when cocultured with Fibs overexpressing FAP1 (Supplemental Figure 16, B and C). These outcomes indicated that the antifibrotic effect of CAR-cTregs may be attributed to contact-induced apoptosis.

To confirm the molecular mechanism of contact-induced apoptosis, neutralizing antibodies were applied to block the PFP, GZMB, FasL, and TRAIL pathways during contact between aFibs and CAR-cTregs (Figure 3C). The results revealed that the addition of a GZMB-neutralizing antibody increased the proliferation of aFibs (Figure 3D) and blocked the antifibrotic effect of CAR-cTregs (Figure 3E). Inhibition of the PFP pathway partially abrogated the cytotoxic effect of CAR-cTregs (Figure 3, D and E). To determine whether CAR-cTregs can suppress the proliferation and cytokine production of Tconvs, we performed an in vitro suppression assay. Like Tregs, CAR-cTregs were able to suppress the proliferation of Tconvs, and this suppressive effect was enhanced upon stimulation with the target protein FAP1 (Figure 3, F and G). The cytokine production of Tconvs was also suppressed by CAR-cTregs, similar to the effect of Tregs (Figure 3H). These results demonstrate that CAR-cTregs are able to suppress the proliferation and activation of Tconv cells.

Next, we evaluated the function of Fibs in vitro. Upon coculture with CAR-cTregs or CAR-Tcs, the increase in the migratory activity of aFibs relative to that of conventional Fibs was significantly attenuated (Supplemental Figure 5, A and B). There was a notable decrease in the activation of aFibs in the group cocultured with CAR-cTregs or CAR-Tcs, as indicated by reduced expression of αSMA, in comparison with that in the group cocultured with UT-Tcs or cTregs (Supplemental Figure 5C). Additionally, the proliferative capacity of aFibs was significantly compromised in the presence of CAR-cTregs or CAR-Tcs (Supplemental Figure 5D). These findings indicate that CAR-cTregs exhibit specific recognition and elimination of target aFibs, effectively modulating their in vitro functional capabilities in a manner similar to that observed with CAR-Tcs.

### CAR-cTregs reverse PF.

**Figure 4:**
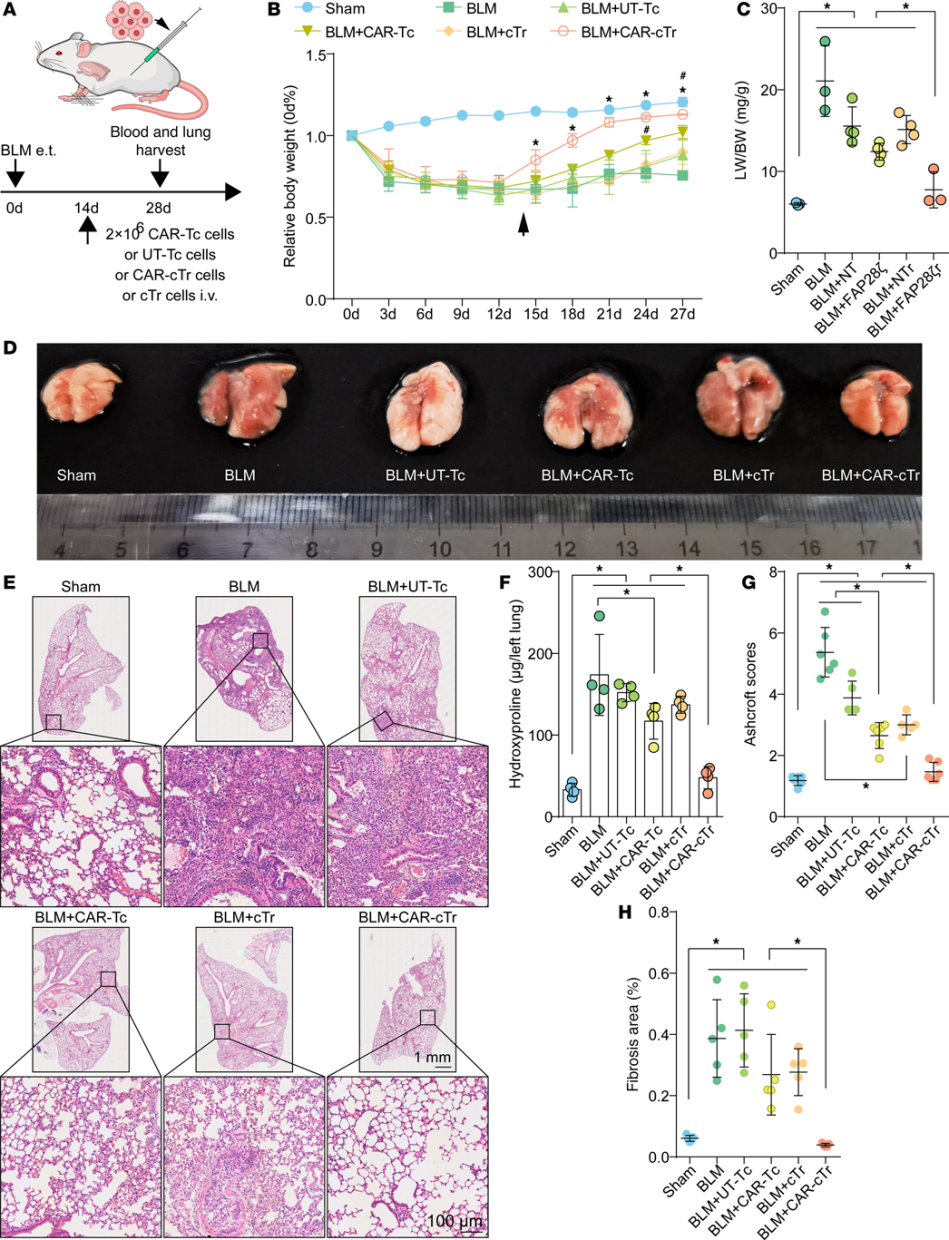
Antifibrotic effect of CAR-cTregs against BLM-induced lung fibrosis. (**A**) Groups of 6- to 8-week-old C57BL/6J mice (*n* = 8) were intratracheally injected with 2.5 U/kg BLM and then adoptively intravenously (i.v.) injected with untransduced T cells (UT-Tc), CAR-Tc, untransduced Tregs (cTregs), or CAR-cTregs (2 × 10^6^ total T cells) on day 14 after BLM infusion. (**B**) Changes in the relative body weight (BW) of mice after BLM infusion (*n* = 8 each group). (**C**) The ratio of lung weight (LW) to BW was also determined following CAR-cTreg treatment (*n* = 8 each group). (**D**) Images of lungs from all the groups are presented. (**E**) H&E staining of lung tissue after T cell infusion. Scale bars: 1 mm (gross views) and 100 μm (higher magnification). (**F**) Hydroxyproline (HYP) concentrations were measured in the presence or absence of CAR-cTreg infusion (*n* = 8 each group). (**G**) Inflammation was quantified by Ashcroft scores (*n* = 8 each group). (**H**) The fibrotic area was analyzed as shown in the bar chart (*n* = 8 each group). *P* values were determined using 2-way ANOVA with Tukey’s post hoc test (**B**) or 1-way ANOVA with Tukey’s post hoc test (**C** and **F**–**H**). (**B**) *P < 0.05 for the comparison between the BLM + CAR-cTreg group and all other groups; ^#^*P* < 0.05 for the comparison between the BLM + CAR-Tc group and all other groups. The data are presented as the mean ± SD (**B**, **C**, and **F**–**H**). *n* = 5 (**B**), *n* = 8 (**C** and **F**–**H**). CAR-cTreg, chimeric antigen receptor cytotoxic effector Treg cell; cTreg, cytotoxic effector Treg cell; CAR-Tc, chimeric antigen receptor cytotoxic T cell; UT-Tc, untransduced cytotoxic T cell.

To evaluate the antifibrotic effect of CAR-cTregs in vivo in a murine BLM model, we injected 2.5 U/kg BLM into the trachea of C57BL/6J mice. Mice were intravenously infused with approximately 2 × 10^6^ CAR-Tcs, UT-Tcs, CAR-cTregs, or cTregs via the tail vein on day 1 or day 14 to determine the optimal therapeutic time point (Figure 4A and Supplemental Figure 7A). CAR-cTreg infusion on day 1 did not significantly restore the changes in body weight (BW), the lung weight (LW)/BW ratio, or fibrosis-associated parameters, including hydroxyproline (HYP) content, inflammatory cell infiltration, and fibrosis area (Supplemental Figure 7, B–H). Histological analysis of the lungs revealed no significant change in the infiltration of inflammatory cells around the bronchi in the CAR-cTreg–injected group (Supplemental Figure 7E). There was no significant reduction in the Ashcroft score in the CAR-cTreg–treated group (Supplemental Figure 7G). CAR-cTregs also did not restore fibrotic responses in the peribronchial region, as evidenced by Masson’s trichrome staining (Supplemental Figure 7H and Supplemental Figure 8A), and increased collagen levels in the lungs compared with those in the controls (Supplemental Figure 7H and Supplemental Figure 8A). These results suggested that early-stage lung inflammation may be protective in the progression of BLM-induced PF and that CAR-cTreg infusion may prolong the duration of inflammation.

However, after infusion on day 14, CAR-cTreg treatment had an excellent therapeutic effect on PF (Figure 4A). After CAR-cTreg infusion, the BW rapidly recovered to the normal level (Figure 4B), the LW/BW ratio decreased (Figure 4C), and the LW was similar to that of the sham group (Figure 4D). To demonstrate the antifibrotic effect of CAR-cTregs, we examined the HYP content and Masson’s staining in the lungs of all groups. CAR-cTreg infusion significantly reduced the HYP content and fibrotic area in the lung (Figure 4, F and H, and Supplemental Figure 6A). In addition, infiltration of inflammatory cells into the lung mesenchyme was significantly reduced (Figure 4E), and the Ashcroft score decreased to a level close to that of the sham group in the CAR-cTreg–injected group (Figure 4G). The expression of the fibrotic signature proteins collagen type 1 α1 (Col I), collagen type 3 α1 (Col III), αSMA, and FAP1 was decreased in the lung tissue of the mice infused with CAR-cTregs (Supplemental Figure 6, B–E). Additionally, CAR-cTregs exerted antifibrotic effects on day 28 after BLM infusion (Supplemental Figure 17, A–I). Thus, CAR-cTreg infusion significantly reversed PF.

### CAR-cTreg infusion does not induce systemic inflammation.

**Figure 5:**
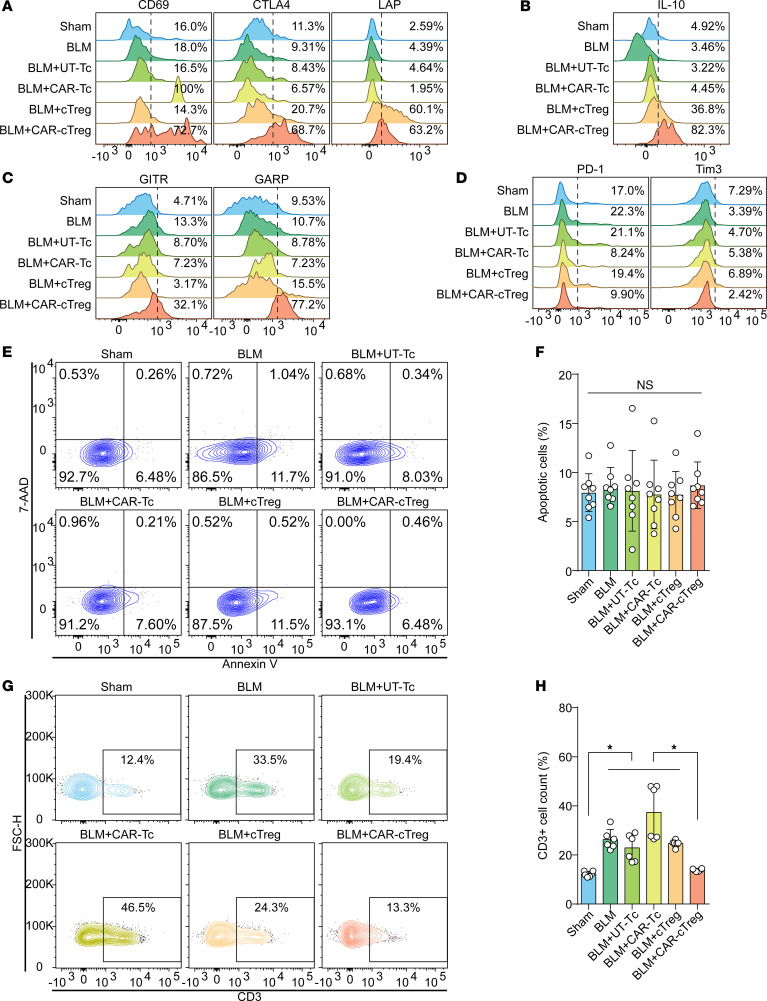
Changes in the phenotype and polyfunctionality of CAR-cTregs in lung tissue. (**A**) The expression of activation markers (CD69, CTLA4, and LAP) was measured by flow cytometry after T cell infusion. (**B**) The secretion of IL-10 was evaluated by intracellular flow cytometry following T cell infusion. (**C**) The expression of Treg activation–associated markers (GITR and GARP) was measured by flow cytometry after T cell infusion. (**D**) The expression of exhaustion markers (PD-1 and Tim3) was measured by flow cytometry after T-cell infusion. (**E**) The percentage of apoptotic T cells in the lung at 2 weeks after T cell infusion is presented as a representative density plot. (**F**) Bar chart of T cell apoptosis in the lung at 2 weeks after T cell infusion (*n* = 8 each group). (**G**) The infiltration of CD3^+^ T cells was evaluated via representative density plots. (**H**) Bar chart of the infiltration of CD3^+^ T cells (*n* = 8). *P < 0.05 by 1-way ANOVA with Tukey’s post hoc test (**F** and **H**). The data in **F** and **H** are presented as the mean ± SD. *n* = 8 (**F**), *n* = 6 (**H**). CAR-cTreg, chimeric antigen receptor cytotoxic effector Treg cell; cTreg, cytotoxic effector Treg cell; CAR-Tc, chimeric antigen receptor cytotoxic T cell; UT-Tc, untransduced cytotoxic T cell.

Next, we determined the phenotype and polyfunctionality of CAR-cTregs in lung tissue. One month after infusion, 60%–70% of the lung CD3^+^ T lymphocytes were CAR-cTregs, as measured by flow cytometry (Supplemental Figure 9A), suggesting the long persistence of the CAR-cTregs. In fibrotic lungs, CAR-cTreg activation was confirmed by the upregulation of activation markers (CD69, CTLA4, and LAP; Figure 5A), Treg activation–associated markers (GITR and GARP; Figure 5C), and cytotoxicity-associated molecules (CD107a, PFP, GZMB, PD-L1, and FasL; Supplemental Figure 13A). There were no differences in exhaustion or apoptosis among the groups (Figure 5, D–F). In addition, intracytoplasmic FACS analysis revealed an increase in the level of the Treg activation–associated cytokine IL-10 (Figure 5B) and no significant increase in the production of other cytokines, including IFN-γ, TNF-α, and IL-6 (Supplemental Figure 12A, C, and E), suggesting the suppression of inflammation in BLM-induced PF. This finding was confirmed by the cell component of the bronchoalveolar lavage fluid (BALF) (Supplemental Figure 6F). Like in the sham group, CAR-cTreg infusion did not increase the infiltration of CD3^+^ T cells (Figure 5, G and H). Phenotypic analysis of infiltrating UT-Tc lymphocytes revealed no significant differentiation into Th1 or Th2 subsets (Supplemental Figure 9, B–E).

In the peripheral blood, T lymphocytes contained 20%–30% CAR-cTregs (Supplemental Figure 11A), and the levels of cytotoxicity-associated molecules, such as CD107a, PFP, and GZMB, were increased in the infused CAR-cTregs (Supplemental Figure 13B). No differences were detected in the expression of representative activation markers (CD69, CTLA4, and LAP; Supplemental Figure 10A), exhaustion markers (PD-1 and Tim3; Supplemental Figure 10D), activation-induced cell death (Supplemental Figure 10, E and F), or Th1/Th2 differentiation after CAR-cTreg infusion (Supplemental Figure 11, B–E). In addition, several Treg activation–associated markers (GARP and LAP) and the cytokine IL-10 were upregulated (Supplemental Figure 10, B and C). The percentage of CD3^+^ T cells increased in the CAR-cTreg infusion group (Supplemental Figure 10, G and H). Therefore, these results suggest that CAR-cTregs reverse PF with less additional inflammation.

### CAR-cTregs lead to remodeling of the fibrotic niche.

**Figure 6:**
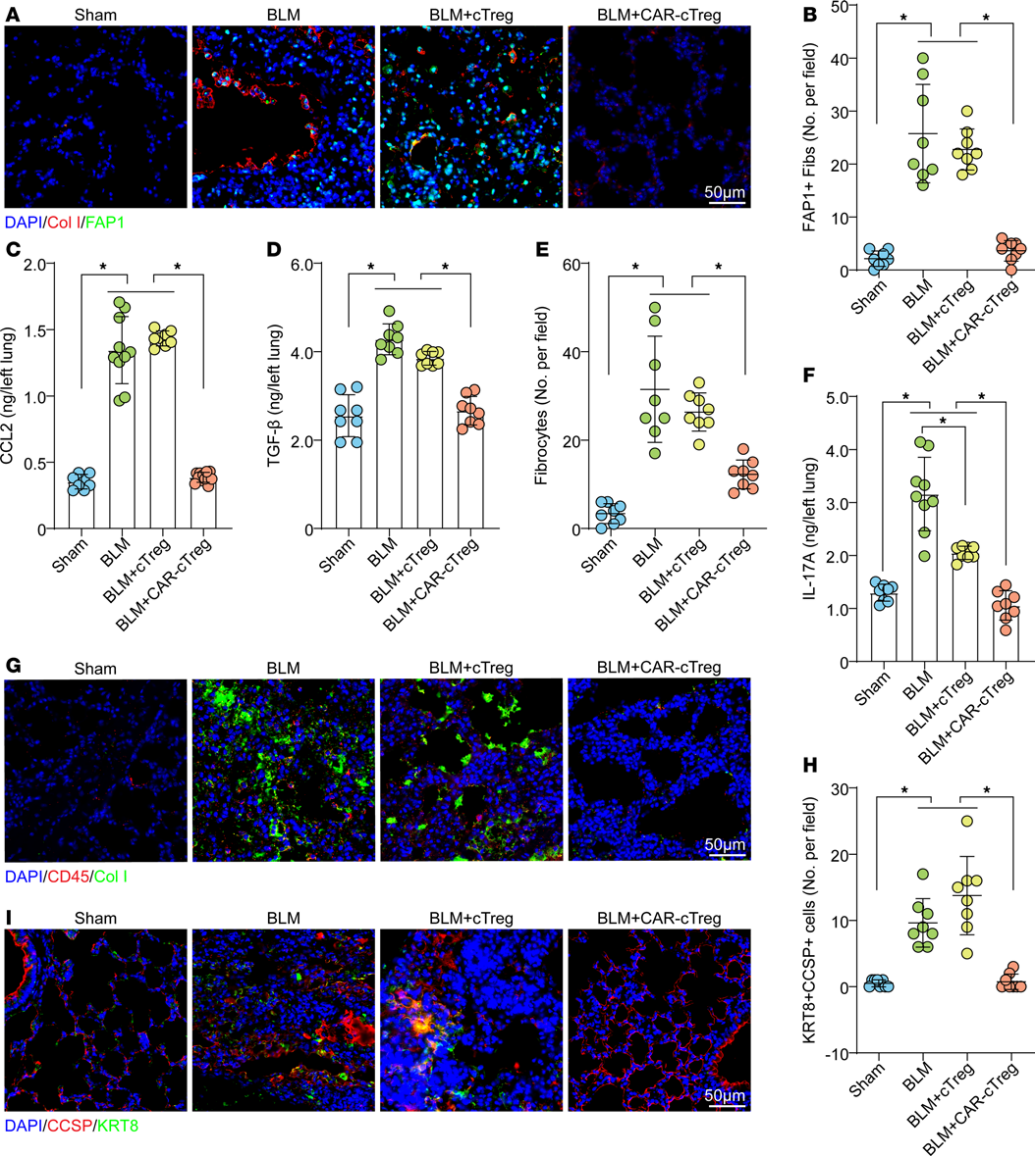
The recruitment of fibrocytes decreased and epithelial cell remodeling improved after treatment with CAR-cTregs. (**A**) Representative fluorescent staining of Col I^+^FAP1^+^ cells in lung tissue after T cell infusion. (**B**) Quantification of Col I^+^FAP1^+^ cells is presented as a bar chart (*n* = 8 each group). (**C**) CCL2 concentrations measured in the presence or absence of CAR-cTreg infusion in the sham, BLM, BLM + cTreg, and BLM + CAR-cTreg (*n* = 8 each group). (**D**) TGF-β1 concentrations measured in the presence or absence of CAR-cTreg infusion in the sham, BLM, BLM + cTreg, and BLM + CAR-cTreg (*n* = 8 each group). (**E**) Quantification of CD45^+^Col I^+^ cells (fibrocytes) is presented as a bar chart (*n* = 8 each group). (**F**) IL-17a concentrations measured in the presence or absence of CAR-cTreg infusion in the sham, BLM, BLM + cTreg, and BLM + CAR-cTreg (*n* = 8 each group). (**G**) Representative fluorescent staining of CD45^+^Col I^+^ cells (fibrocytes) in lung tissue after T cell infusion. (**H**) Quantification of KRT8^+^CCSP^+^ cells is presented as a bar chart (*n* = 8 each group). (**I**) Representative fluorescent staining of KRT8^+^CCSP^+^ cells in lung tissue after T cell infusion. *P < 0.05 by 1-way ANOVA with Tukey’s post hoc test (**B**–**F** and **H**). The data in **B**–**F** and **H** are presented as the mean ± SD. *n* = 8 (**B**), *n* = 8–11 (**C**), *n* = 8 (**D**), *n* = 8 (**E**), *n* = 8–9 (**F**), *n* = 8 (**H**). Scale bars: 50 μm. CAR-cTregs, chimeric antigen receptor cytotoxic effector Treg cells; cTregs, cytotoxic effector Treg cells.

Within the context of BLM-induced PF, a significantly increase of approximately 25-fold in the aFib population was noted. However, posttreatment with CAR-cTregs resulted in a pronounced decrease in the aFib count, which was consistent with the findings in the sham group (Figure 6, A and B, and Supplemental Figure 18, A and D). The number of FAP1^+^ nonfibroblasts did not significantly change after CAR-cTreg infusion, likely due to the low abundance of these cells, which also reflects the good safety profile of CAR-cTregs (Supplemental Figure 18, A and D). The pathogenesis of PF is hypothesized to arise from a multifaceted interplay involving cellular mechanisms of structural cell death and the subsequent infiltration of inflammatory leukocytes, including collagen-producing fibrocytes and differentiated Th17 cells. Bone marrow–derived circulating fibrocytes (23) can be recruited to sites of tissue injury by chemokines, such as CCL2 (24), and actively participate in the fibrotic cascade (25), including the activation of profibrotic growth factors such as TGF-β1 and myofibroblast transformation (26). Given that Tregs have been shown to significantly ameliorate these fibrogenic transformations (27), we measured the levels of the fibrocyte chemoattractant CCL2 and the number of fibrocytes in BLM-induced fibrotic lungs. The adoptive transfer of CAR-cTregs significantly attenuated the increased expression of CCL2 and TGF-β1, which are characteristic of PF (Figure 6, C and D), coincident with a decrease in the size of the fibrocyte population (Figure 6, E and G, and Supplemental Figure 18, B and E). Th17 cells have been implicated in the pathogenesis of BLM-induced PF and can promote the proliferation, differentiation, and inflammatory cytokine generation of fibrocytes via the secretion of IL-17a (28). Considering the inhibitory effect of Tregs on the differentiation and function of Th17 cells (28), we analyzed the concentration of IL-17a in fibrotic lung tissue. The results revealed that the pulmonary levels of IL-17a in mice subjected to BLM were significantly decreased, approximating those in sham-operated mice, 2 weeks after CAR-cTreg infusion (Figure 6F). This regulatory effect was further corroborated in vitro through coculture experiments involving Th17 cells, which demonstrated that CAR-cTregs had more pronounced suppressive effects (Supplemental Figure 14A).

Current research has revealed an additional salient characteristic of PF, which involves the continual presence of transitional cells with apparent origins from both alveolar epithelial cell type 2 (AEC2s) and club cell secretory protein 1–expressing (CCSP1-expressing) airway cells. These cells are characterized by elevated expression of transcripts related to TGF-β1 signaling pathways, ECM components, and genes associated with cell-cycle arrest and senescence. Furthermore, these transitional cells can be specifically identified by the marker cytokeratin 8 (KRT8) (29–35). To ascertain the degree to which epithelial cells remodeling during chronic PF, we evaluated KRT8^+^CCSP^+^ cells. In the fibrotic lung parenchyma, there was a considerable decrease in KRT8^+^CCSP^+^ transitional cells after CAR-cTreg infusion (Figure 6, H and I, and Supplemental Figure 18, C and F). Therefore, these findings suggested that the infusion of CAR-cTregs leads to a reduction in the mobilization of fibrocytes, the suppression of the profibrotic cytokine IL-17a, and an improvement in the remodeling of AECs.

## Discussion

In the present study, we identified FAP1^+^ AF cells as the main collagen-producers in IPF (Figure 1 and Supplemental Figures 19–58). Given the suboptimal therapeutic outcomes of CAR-T cell therapy for PF due to severe CRS, CAR-cTregs were generated to specifically target and eliminate aFibs, and their in vitro cytotoxic and suppressive effects were confirmed. In the BLM model, CAR-cTreg infusion reversed PF (Figure 4) without infiltration of inflammatory cells (Supplemental Figure 9) or systemic inflammation (Supplemental Figure 12). In this context, we further showed that the remarkable antifibrotic efficacy of CAR-cTregs is characterized by a decrease in the recruitment of fibrocytes, curtailing the production of the profibrotic cytokine IL-17a, and facilitating the remodeling of AECs. Therefore, CAR-cTregs, which are endowed with cytotoxic and immunosuppressive properties, were found to reverse BLM-induced PF and improve the fibrotic niche, providing a promising therapeutic strategy for the treatment of IPF.

More recently, single-cell atlases of normal lungs and IPF lungs have been generated. AF1 is enriched in genes associated with collagen-producing Fibs, such as elastin (*Eln*), PDGFRα (*Pdgfra*), and collagen (*Col1a1*) (36), and contains lipofibroblasts and matrix Fibs, whereas AF2 is another source of matrix Fibs and is responsible for the matrix structure of the lung. AF1 contributes to the majority of αSMA^+^ myofibroblasts (36). The ability of AF1 to upregulate *Acta2* (which encodes αSMA) and differentiate into myofibroblasts after BLM injury and the relative inability of these cells to support AT2 cell growth in organoids (36). In addition, PDGFRα-expressing lipofibroblasts differentiate into myofibroblasts upon injury and transdifferentiate back to lipofibroblasts during fibrosis resolution in the mouse lung (37, 38). Thus, AF1 is the main Fib subset associated with PF (18, 39). Furthermore, we found that FAP1^+^ AF1 cells represent a more distinct subpopulation that is closely associated with differentiating into myofibroblasts (36) and producing collagen in the context of PF (Supplemental Figures 19–58).

Multiple studies have identified aFibs as an activated subset of Fibs, which is distinct from the traditionally recognized “myofibroblasts” that express αSMA, but with some overlap (20, 21). This cell subset is primarily distributed in areas undergoing tissue remodeling, including wound healing, arthritis, tissue fibrosis, atherosclerosis, and the tumor microenvironment (21), and is less prevalent in most normal adult tissues (20). However, the specific roles of aFibs remain unclear. The gene expression profile of aFibs is enriched mainly in inflammation-related pathways, the ECM, matrix remodeling enzymes, and epithelial cell growth factors (40). On the other hand, FAP1 is a cell surface protease involved in collagen degradation and collagen turnover (41). The primary role of aFibs may involve degrading matrix and matrix turnover through the enzymatic activity of FAP1 and other matrix-degrading enzymes, while producing collagen and other matrix components to participate in fibrosis. These aspects require further research.

Due to the enormous success of CAR-T cell therapy in homology oncology (42), this strategy provides an approach allowing these synthetic immune cells to recognize and eliminate sickened cells in a powerful and precise manner. Accordingly, substantial effort has been made to apply CAR-based therapy to a wide range of disease contexts, such as autoimmune diseases (e.g., systemic lupus erythematosus) (43), infectious diseases (44), liver disease, and diabetes (45).

Previous studies have shown that FAP^+^ CAR-T cell therapy exacerbates fibrosis in a BLM-induced PF model (21). However, in our study, CAR-T cells still had partial therapeutic effects. Albelda et al. (21) reported that the number of FAP1^+^ cells decreased after CAR-T cell therapy but still differed significantly from that in the control group, indicating suboptimal CAR-T cell cytotoxicity. Second, the percentage of CD45^+^ cells alone does not fully reflect pulmonary inflammation or CAR-T–associated CRS, as inflammation can also drive fibrosis. Therefore, inadequate CAR-T cell therapy efficacy and CRS may contribute to worsened fibrosis. In light of these potential factors, we have shifted our focus to a CAR-based cell subset — cTregs.

In some noncancerous diseases, mild inflammation affects the progression of the disease. Engineered Tregs have also been indicated as a promising therapy for chronic inflammatory disease, which ultimately restricts or resolves inflammation. CAR-based technology provides significant promise for directing Tregs specifically and potently toward an antigenic target. CAR-Tregs have been used to modulate immune responses in multiple disease conditions, including GVHD (46), multiple sclerosis (22), inflammatory bowel disease (47), asthma (48), vitiligo (49), and hemophilia (50). Compared with Tregs, infused CAR-Tregs have shown longer survival times in recent clinical trials. Extensive research has indicated that CAR-Tregs exhibit minimal cytotoxicity (51). Furthermore, due to the plasticity of Tregs, CAR-Tregs are prone to transdifferentiate into proinflammatory Tregs, which may attenuate therapeutic efficacy (52). cTregs are Treg subsets that possess both cytotoxic functions and immunosuppressive capabilities (15). Adoptive transfer of cTregs suppresses GVHD while effectively preserving GVL activity (16, 17). In the present study, the overexpression of FoxP3 in CAR-cTregs stabilized the phenotype of antiinflammatory Tregs. Our work confirmed that CAR-cTregs not only suppress the inflammatory microenvironment in diseased regions but also have a certain cytotoxic ability similar to that of CAR-T cells. However, further studies are needed to confirm the effect of this CAR-cTreg strategy in other inflammatory diseases.

In conclusion, we identified FAP1^+^ AF cells as collagen producers in IPF. CAR-cTregs are used to control potential inflammation and induce cytotoxicity in aFibs. CAR-cTreg infusion reversed PF without systemic inflammation characterized by diminished recruitment of fibrocytes, curtailing the production of the profibrotic cytokine IL-17a, and facilitating the remodeling of AECs, which provides a promising therapeutic strategy for the treatment of IPF. However, further clinical trials are needed to test the effect of CAR-cTregs in patients with IPF.

## Methods

### Sex as a biological variable.

Our study examined male mice because male animals exhibited less variability in phenotype. It is unknown whether the findings are relevant for female mice.

### Human tissues.

All IPF samples were obtained via either lung transplantation surgery or lung biopsy procedures. The diagnosis and classification of IPF for each individual were determined by their primary pulmonologist according to American Thoracic Society/European Respiratory Society guidelines. Healthy lung tissue samples were obtained from organ donors who were diagnosed with brain death. Prior to tissue collection, written informed consent for tissue harvesting was obtained from each patient or their family members.

### FAP1-CAR generation.

The scFv fragment was cloned from a mouse-specific anti-FAP monoclonal antibody (clone 73.3) via published methods (53). The FAP1 CAR construct contained the FAP1 scFv fragment with mouse CD3ζ and CD28 intracellular signaling domains (54). The murine FoxP3 gene was inserted downstream of the CAR gene, with a P2A peptide sequence facilitating their segregation. After codon optimization for expression in mammalian cells, the full sequence of the FAP1 CAR was cloned and inserted into a retroviral vector. Surface expression was determined by flow cytometry with transfected spleen T cells (Lipofectamine 2000, Life Technologies). Viral particles were produced as previously described (55).

### Transduction of primary lymphocytes.

To generate FAP1 CAR-T cells, splenocytes were isolated and activated on non–tissue culture 6-well plates precoated with anti-mCD3 (30 ng/mL; BioLegend, 100340) and anti-mCD28 (30 ng/mL; BioLegend, 102116) antibodies for 24 hours. On day 5, activated T cells (4 × 10^6^ cells/well) were sorted for CD8^+^ T cells (STEMCELL Technologies, 19853) and transduced with the retroviral supernatant of the FAP1 CAR on RetroNectin (Takara, T100B) 6-well plates coated with 100 U/mL IL-2 (Sino Biological, 51061-MNAE). The transduced murine lymphocytes were expanded for 24 hours in the presence of 350 U/mL IL-2. The transduction efficiency was estimated by analyzing CAR-T cell surface expression on retrovirally transduced cells and comparing it to that on UT-Tc cells.

### Treg sorting, transduction, and expansion.

Murine splenocytes were isolated and activated with anti-mCD3 antibodies (30 ng/mL; BioLegend, 100340), anti-mCD28 antibodies (30 ng/mL; BioLegend, 102116), 1,000 U/mL IL-2 (Sino Biological, 51061-MNAE), 1 μM AS2863619 (MedChemExpress, HY-126675), 1 nM retinoic acid (MedChemExpress, HY-14649), and 2 ng/mL TGF-β1 (Sino Biological, 80116-RNAH) for 5 days. CD8^+^CD25^+^ Tregs were purified from stimulated T cells via magnetic cell sorting (Miltenyi Biotec) prior to sorting into live CD4^+^CD45RO^lo^CD45RA^hi^CD25^hi^ Tregs and CD4^+^CD45RO^lo^CD45RA^hi^CD25^lo^ Tconv cells via a FACSAria II (BD Biosciences). Sorted T cells were stimulated with artificial APCs (aAPCs) loaded with anti-CD3 mAbs as previously described (56) at 1,000 U/mL or 100 U/mL IL-2 for Tregs or Tconvs, respectively. One day later, the cells were transduced with retrovirus at an MOI of 10 virus particles/cell. On day 8, CAR^+^ cells were purified via magnetic selection (Miltenyi Biotec), restimulated with aAPCs as described above, and expanded for 1 day.

To test the effects of FAP1-mediated stimulation, Tregs were restimulated with primary lung Fibs or aFibs (derived from lung fibroblasts activated with 20 ng/mL TGF-β1 for 24 hours) at a 1:2 (fibroblast/T cell) ratio for 24 hours.

### Flow cytometry.

For phenotypic analysis, the cells were stained with Fixable Viability Stain 780 (BD Biosciences, 565388) and surface markers before fixing/permeabilizing with a FOXP3/Transcription Factor Staining Buffer Set (eBioscience), followed by staining for intracellular proteins. For analysis of cytokine production, the cells were stimulated with 10 ng/mL PMA (Sigma-Aldrich) and 500 ng/mL ionomycin (Sigma-Aldrich) in the presence of brefeldin A (10 μg/mL; Sigma-Aldrich) for 4 hours. The samples were read on a FACSAria SORP (BD Biosciences), and the results were analyzed via FlowJo Software version 10.0.6 (Tree Star). Surface staining was performed for F(ab)2 (Jackson ImmunoResearch, 115-066-006), CD4-PE-Cy7 (BioLegend, 100433), CD4-FITC (BioLegend, 100510), CD8- PerCP-Cy5.5 (BioLegend, 100734), CD8-PE (BioLegend, 100708), CD8-APC (BioLegend, 155006), CD44-BUV421 (BD Biosciences, 563970), CD44-PE-Cy7 (BioLegend, 103031), CD44-PerCP-Cy5.5 (BioLegend, 162106), CD25-APC (BD Biosciences, 557192), LAP-PerCP-Cy5.5 (BioLegend, 141409), GARP-PE (BioLegend, 142907), CD69-PE (BioLegend, 104507), CD69-APC (BioLegend, 104513), CD127-PE (BioLegend, 552543), Ki67 (Abcam, ab16667), Dylight 405 (Beyotime, A0605), AF488 (Cell Signaling Technology, 4408S), PD-1-PE (BioLegend, 135206), CTLA4-APC (BioLegend, 106309), CXCR3-PerCP-Cy5.5 (BioLegend, 126514), CD62L-PE (BioLegend, 104408), CD3e-BUV395 (BD Biosciences, 565992), PE-Cy7 (BioLegend, 405206), FoxP3-BUV395 (BioLegend, 126419), GITR-FITC (BioLegend, 120205), Tim3-APC (BioLegend, 119705), CCR4-PE (BioLegend, 131204), CD107A-APC (BioLegend, 121613), PFP-FITC (BioLegend, 154309), GZMB-PE (BioLegend, 372207), IL-10-FITC (BioLegend, 505005), CD103-PE (BioLegend, 121405), PD-L1-APC (BioLegend, 124311), and FasL-PE (BioLegend, 106605).

### Proliferation, activation, cytokine production, and antigen-nonspecific suppression.

To assess proliferation and activation, T cell lines were labeled with CPD (eBioscience, 65-0840-85 or 65-0842-85) and stimulated with Fibs loaded for 1 hour of preincubation with anti-CD3 and anti-CD28 mAbs (1 μg/mL each) or aFibs at a 1:2 (aFib/T cell) ratio. Antigen-nonspecific suppression was assessed with Tconvs labeled with CPD450 and stimulated via anti-CD3/anti-CD28–coated beads (Invitrogen) at a 1:8 or 1:16 bead-to-Treg ratio for 24 hours while keeping the Tconv numbers constant. CAR-cTregs were stimulated with 1 mg/mL FAP1 protein (Sino Biological, 10464-H07H). The proliferation of Tconv cells was quantified relative to that of unstimulated cells via cell counting. The percentage of suppression of CD3^+^ cells was calculated via the division index (DI) as follows: 100 – ([DI Tconvs + test]/[DI Tconvs]) × 100. To measure cytokine production, T cell lines were stimulated with the indicated Fibs (1 Fib:2 T cells) for 24 hours. The supernatants were collected, and the cytokine concentrations were determined with a Human Th1/Th2 Cytokine Kit (BD Biosciences) and analyzed with LEGENDplex 8.0 data analysis software.

### IFN-γ secretion.

Murine lung fibroblasts (1.2 × 10^5^ cells) were seeded in 24-well plates, followed by the addition of murine CAR-T cells (2.5 × 10^5^ cells) to complete RPMI 1640 media. The cell culture supernatants were harvested and assayed for IFN-γ 18 hours later via a mouse or human IFN-γ ELISA kit (R&D Systems) according to the manufacturer’s instructions.

### Cytotoxicity.

For the specific lysis assays, lung Fib targets (1 × 10^4^ cells) were cocultured with varying amounts of CAR-Tc, CAR-cTreg, UT-Tc, or cTregs for 18 hours. Cytotoxicity was measured via an LDH cytotoxicity assay (Sigma-Aldrich, 11644793001).

Cell counting was employed to perform precise cytotoxicity assays using GFP-expressing Fibs. After 18 hours of coculture, the GFP fluorescence of the cells was photographed and recorded via a Leica DMi8 fluorescence microscope. The fluorescent cells were counted, and all the experiments were performed in triplicate.

### In vivo experiments.

PF was initiated by one intratracheal instillation of bleomycin (2.5 U/kg) while the mice were under isoflurane anesthesia, and a laryngoscope was used to visualize the trachea. Eight-week-old male C57BL/6J mice were purchased from HFK Bio-Tech. Fibrosis was assessed by quantifying lung collagen by measuring HYP in the upper right lobe. Peripheral blood from the saphenous vein was centrifuged; then, erythrocytes were lysed, leukocytes were measured by flow cytometry, and plasma was aspirated and frozen at –80°C until use. The mice were sacrificed at the indicated time points, and tissue samples for histology were fixed in 10% formalin and embedded in paraffin.

### Histology.

Pathological changes were evaluated by H&E and Masson’s trichrome staining. H&E staining was used to assess the severity of interstitial fibrosis on a scale ranging from 0 to 8 (0 = normal lung; 8 = very severe fibrosis) via the Ashcroft method. For trichrome staining, collagen fibers were stained blue; muscle fibers, cytoplasm, and erythrocytes were stained red; and nuclei were stained black/blue. Immunohistochemistry was performed on paraffin sections via antibodies against αSMA (1:1,000; Cell Signaling Technology, 19245S), collagen 1 (1:400; Abcam, ab6308), collagen 3 (1:400; Abcam, ab6310), and FAP1 (1:200; Abcam, ab28244). Staining was visualized via the application of a red chromogenic substrate (Dako, K60404).

Ten images per mouse were assessed in a blinded manner on a Zeiss Axioskop 2 Plus. For quantitative analysis, images were acquired blindly with a 10× objective, and analysis was performed via Fiji software version 2.0.0-rc-30/1.49v (ImageJ; NIH).

### Isolation and culture of primary lung Fibs.

After euthanasia, the mice were perfused with 5 mL of cold PBS solution through the left or right ventricle. The lungs were dissociated in a C tube (Miltenyi Biotec) in digestion buffer (RPMI 1640 supplemented with collagenase type III [200 U/mL; Worthington] and DNase I [200 μg/mL; Sigma–Aldrich]) via a gentleMACS tissue dissociator (Miltenyi Biotec). After 30 minutes at 37°C, the samples were subjected to gentleMACS dissociation. The cell suspensions were passed through a 70-μm cell strainer (Greiner Bio-One) and cultured on poly-L-lysine–precoated (Beyotime, ST509) 6-well plates for 72 hours. To obtain FAP1^+^ fibroblasts (aFibs), 20 ng/mL TGF-β1 was added to primary lung Fibs for 24 hours, and an equal volume of PBS was added as a control.

To obtain Fibs overexpressing FAP1, primary lung fibroblasts were infected with an adenovirus overexpressing FAP1 (Genechem) via EndoFectin MAX (GeneCopoeia, EF013) according to the manufacturer’s protocol. Two days after transfection, the cells were used for subsequent experiments. For FAP1 knockdown experiments, primary lung Fibs were transfected with FAP1 small interfering RNA (siRNA) or negative control (GeneChem) using EndoFectin MAX following the manufacturer’s protocol. The sequences of the siRNAs are presented in Supplemental Table 4. The final concentration was 50 nM. The transfected cells were utilized for experiments after 24 hours.

### Wound healing assay.

The cells were grown to near-confluence in 6-well plates and subjected to serum-free medium for 24 hours of starvation. The monolayers were scratched using a sterile 1 mL tip, followed by an additional 48 hours of starvation. An inverted microscope (Olympus IX73) was used to capture images of the cells that migrated to the corresponding wound sites at 0 and 48 hours.

### BAL and differential cell counts.

When the mice were sacrificed, the lungs were removed and washed in cold PBS. BALF was obtained by cannulating the trachea and injecting and retrieving 1 mL aliquots of sterile physiological saline 3 times. The BALF was centrifuged at 300*g* for 8 minutes at 4°C. After RBC lysis, the BAL cell pellet was washed and resuspended in PBS. The total cell counts were determined via standard hematologic procedures. A BAL cytospin was prepared and stained via the Wright-Giemsa method. Monocytes, basophils, eosinophils, neutrophils, or lymphocytes were identified on the basis of 200 cells via standard morphological criteria.

### Neutralizing antibody administration.

The coculture mixture of Tregs and Fibs was supplemented with neutralizing antibodies (all at 100 μg/mL final concentration) against PFP (Invitrogen, PA5-109315), GZMB (Invitrogen, PA5-47214), FasL (Invitrogen, 16-5911-82), TRAIL (Invitrogen, 14-5959-85). IgG2b (BioLegend, 400622) was added as the isotype control.

### Construction of GFP-expressing Fibs.

The reporter plasmid pCDH-EGFP, encoding GFP under the control of the Chinese hamster elongation factor 1α (CHEF1) promoter was used for all experiments (Miaolingbio). The plasmid was transformed and amplified in *E*. *coli* DH5α competent cells (Tsingke Biotechnology) via Amphenol selection for positive transformants. Plasmid DNA from overnight cultures of *E*. *coli* grown in LB media (37°C, 220 rpm) was purified via a Plasmid Midi Kit (Qiagen) according to the manufacturer’s protocol. The plasmid concentration and purity were assessed with a NanoDrop One spectrophotometer (Thermo Fisher Scientific) at 260 nm. 293T cells in good condition and in the logarithmic growth phase were packaged with lentivirus. pCDH-EGFP, pMD2.G, and psPAX2 plasmids were used for transfection. The supernatant of the lentivirus particles was collected 72 hours after transfection. Centrifugation (50,000*g*, 2 hours, 4°C) was carried out on the collected supernatant of the lentivirus particles. The supernatant was carefully removed, and the lentivirus particles were dissolved in a small amount of culture medium (4°C, >2 hours) and stored at –80°C. When the virus was used to transfect primary lung Fibs, obvious fluorescence was detected 48 hours after transfection. G418 (Solarbio) screening began 3–4 days after transfection.

### High content analysis.

GFP-expressing Fibs were seeded onto 96-well plates at 1,000 cells/well. After 24 hours, the cTregs and CAR-cTregs were labeled with DiD (Beyotime, C1039) for 30 minutes at 37°C and then loaded onto plates for 6 hours at 37°C at 1:5 (Fib/Treg). The fluorescence was monitored briefly for aFibs in multiple fields for all wells. Image acquisition and data processing were carried out on an Opera Phenix Plus high-cContent screening system (PerkinElmer) with a 63× lens.

### Immunofluorescent staining.

The tissue sections were stained with primary antibodies against FAP1 (1:1,000; Abcam, ab28244), collagen 1 (1:400; Millipore, AB765P), CD45 (1:100; Invitrogen, MA5-11532), KRT8 (1:200; Invitrogen, MA5-14428), and CCSP (1:1,000; Proteintech, 26909-1-AP). Secondary antibodies conjugated with fluorescent dyes were utilized at a dilution of 1:100 (Invitrogen). A total of 10 images per specimen were evaluated in a manner blinded to the treatment conditions on a Zeiss Axioskop 2 Plus.

### ELISA.

Mouse lung CCL2, TGF-β1, and IL-17a levels were measured via ELISA kits purchased from R&D Systems. To quantify the levels of CCL2, TGF-β1, and IL-17a in pulmonary tissue, the right lung from each murine subject was immersed in 1 mL of ice-cold lysis buffer composed of 100 mM Tris-HCl (pH 7.4), 150 mM NaCl, 1 mM EDTA, and a cocktail of protease inhibitors (Complete Mini; Roche Diagnostics). The lung homogenates were subsequently prepared via a 2-mL tissue grinder (Wheaton Industries). Following the centrifugation of the lung homogenate at 10,000*g* for 5 minutes at 4°C, the supernatant fraction was carefully collected. The concentration of CCL2 in the supernatant was then determined via ELISA kit. The supernatants from each sample were serially diluted in PBS, and an aliquot of 100 μL of the diluted samples was subjected to analysis via the respective assay kits. The optical density (OD) of each sample was quantified at a wavelength of 450 nm via a microplate reader. All the samples were measured in duplicate.

### Bioinformatics analysis.

scRNA-seq data (GEO GSE128033) were used for bioinformatics reanalysis. After canonical correlation analysis, the scRNA-seq data were clustered and visualized with uniform manifold approximation and projection (UMAP) and t-distributed stochastic neighbor embedding (t-SNE). The identification of cell clusters was performed on the final aligned object guided by CellCards (18). DEG analysis was performed for AF1 cells and AF2 cells between healthy donors and patients with PF via the MAST package. Functional enrichment analysis of DEGs was conducted via the PANTHER database (57). UMAP plots, t-SNE plots, violin plots, and dot plots were generated via Seurat (https://satijalab.org/seurat/). To calculate module scores and AUC values for fibrosis-related pathways in single cells, AddModuleScore() and AUCell() were used. The fibrosis-related pathway genes were collected from the Biological Process category of Gene Ontology, the KEGG pathway database, and the Reactome pathway database.

### Statistics.

The data are expressed as the mean ± SD and were analyzed via GraphPad Prism 7.0 software. Two-tailed, unpaired *t* tests were performed for comparisons between 2 groups. One-way ANOVA with Tukey’s post hoc test was used for multiple comparisons. The nonparametric Mann-Whitney *U* test was used to compare 2 samples. A *P* value of less than 0.05 was considered to indicate statistical significance. NS, not significant. All the experiments were performed at least 3 times.

### Study approval.

The investigation involving clinical samples was approved by the Ethics Committee of West China Hospital, Sichuan University (approval no. 2022794). Prior to tissue collection, written informed consent for tissue harvesting was obtained from each patient or their family members. All experiments involving mice were approved by the Animal Ethics Committee of West China Hospital, Sichuan University (approval no. 2021848A) and were performed according to the Animal Ethics Committee’s guidelines.

### Data availability.

All data associated with this study are available in the main text or in the supplemental Supporting Data Values file.

## Author contributions

YHJ and YQG designed the research. YHJ, MZ, MDC, and SC conducted the experiments. YHJ, MZ, and MDC were involved in the analysis and interpretation of the data. SC and YQG provided overall study supervision. YHJ, MZ, MDC, and YQG wrote the manuscript.

## Supplementary Material

Supplemental data

Supplemental table 3

Supplemental video 1

Supplemental video 2

Supplemental video 3

Supplemental video 4

Supporting data values

## Version 1 07/08/2025

In-Press Preview

## Version 2 08/08/2025

Electronic publication
