# Supplementary material for: CAR-engineered cytolytic Tregs reverse pulmonary fibrosis and remodel the fibrotic niche with limited CRS
Source: JCI Insight. 2025 Jul 8;10(15):e182050. doi: 10.1172/jci.insight.182050 (PMC12333945; doi:10.1172/jci.insight.182050)
Supplement: Supplemental data [file jciinsight-10-182050-s174.pdf]

Supplemental material

Table S1. Characteristics of healthy donors and patients with pulmonary fibrosis

No.	Sample ID	Original sample ID	Diagnosis	Age (y)	Sex	Pathological assessment of adjacent lung tissue
**1**	Donor1	SC249		N/A	N/A	Normal healthy lung
**2**	Donor2	SC228		N/A	N/A	Normal healthy lung
**3**	Donor3	SC156		23	F	Normal healthy lung
**4**	Donor4	SC155		23	F	Normal healthy lung
**5**	Donor5	SC59		18	M	Healthy lung tissue; a bit of bronchitis probably due to ventilator. Some of the small air ways are prominent and more muscular than expected, which may indicate that this patient had allergies or some asthma
**6**	Donor6	SC56		57	F	Normal healthy lung
**7**	Donor7	SC45		55	M	Normal healthy lung
**8**	Donor8	SC31D		56	M	Normal healthy lung
**9**	Donor9	SC31		56	M	Normal healthy lung
**10**	Donor10	SC14		76	M	Normal healthy lung
**11**	IPF1	SC154	IPF	69	F	Nearly complete replacement of the lung architecture by scar tissue with honeycomb change with end-stage lung disease
**12**	IPF2	SC153	IPF	69	F	Complete replacement of the lung architecture by scar tissue with honeycomb change and end-stage lung disease
**13**	IPF3	SC95	IPF	69	M	Classic end-stage lung: honeycomb cysts, bronchial metaplastic cells, some smooth muscle metaplasia
**14**	IPF4	SC94	IPF	69	M	Scarring around the airways, smoker, much better than the lower lobe. Some of the scarring may be from previous acute exacerbations. No UIP or fibrosis, maybe some COPD
**15**	IPF5	SC93	IPF	69	M	Classic end-stage lung: honeycomb cysts, bronchial metaplastic cells, some smooth muscle metaplasia
**16**	IPF6	SC89	IPF	70	F	UIP: acute exacerbation of UIP, diffuse alveolar damage and inflammation
**17**	IPF7	SC88	IPF	70	F	Mostly UIP; more diffuse than lower lobe. Some mixed fibrotic and cellular NSIP (acute) Some honeycomb cysts
**18**	IPF8	SC87	IPF	70	F	UIP: acute exacerbation of UIP, diffuse alveolar damage and inflammation

IPF, idiopathic pulmonary fibrosis; UIP: usual interstitial pneumonia; NSIP: nonspecific interstitial pneumonia; COPD: chronic obstructive pulmonary disease; F, female; M, male; y, year; N/A, not available.

Table S2 Fibrosis-related pathway from the BP category in the GO, KEGG PATHWAY and Reactome Pathway

Identifier ID	Term
**BP category in GO**	
GO:0085029	Extracellular matrix assembly
GO:1901201	Regulation of extracellular matrix assembly
GO:1901203	Positive regulation of extracellular matrix assembly
GO:0030198	Extracellular matrix organization
GO:1903053	Regulation of extracellular matrix organization
GO:1903055	Positive regulation of extracellular matrix organization
**KEGG PATHWAY**	
N01814	Extracellular matrix - Basal lamina
**Reactome Pathway**	
R-HSA-2214320	Anchoring fibril formation
R-HSA-1650814	Collagen biosynthesis and modifying enzymes
R-HSA-8948216	Collagen chain trimerization
R-HSA-2243919	Crosslinking of collagen fibrils
R-HSA-2022090	Assembly of collagen fibrils and other multimeric structures
R-HSA-1474290	Collagen formation
R-HSA-2129379	Molecules associated with elastic fibres
R-HSA-1566948	Elastic fibre formation
R-HSA-3000178	ECM proteoglycans
R-HSA-1566977	Fibronectin matrix formation

BP, Biological process; GO, Gene Ontology; KEGG, Kyoto Encyclopedia of Genes and Genomes.

Table S3 Functional enrichment analysis of DEGs in AF1 cells and AF2 cells

Table S4 The sequences of the siRNAs for FAP

	Sequence
**siNC**	5'-UUCUCCGAACGUGUCACGUTT-3'
	5'-ACGUGACACGUUCGGAGAATT-3'
**siFAP**	5'-CCUUAGCAAUGGAGAAUUUTT-3'
	5'-AAAUUCUCCAUUGCUAAGGTT-3'

Movie S1-S3 CAR-cTrs (shown in red) induced apoptosis in GFP-expressing aFibs (arrow) through a contact-dependent mechanism, as shown by imaging for 6 h.

Movie S4 cTrs (shown in red) did not inhibit the proliferation of GFP-expressing aFibs after imaging for 6 h.

Figures

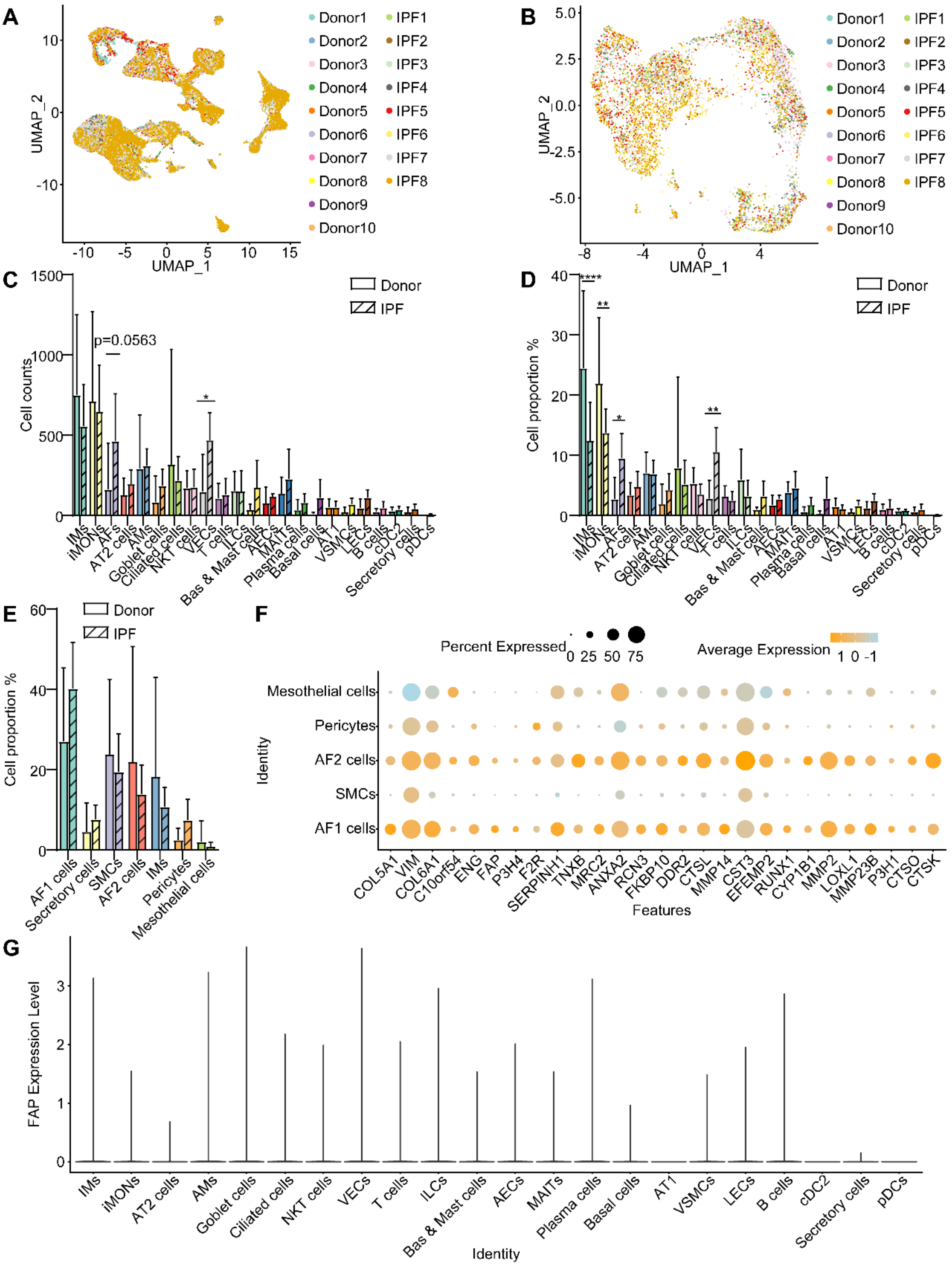

Figure S1. Changes in cell composition in IPF. A. The UMAP of single-cell RNA sequencing in healthy donors (HDs, n=10) and patients with IPF patients (n=8). B. The UMAP analysis of the mesenchymal cells in HDs (n=10) and IPF patients (n=8). C. The absolute count of the 23 subsets in HDs (n=10) and IPF patients (n=8). D. The ratio of the 23 subsets in HDs (n=10) and IPF patients (n=8). E. The ratio of the 5 stromal subsets in HDs (n=10) and IPF patients (n=8). F. The expression of 27 selected genes

is shown in alveolar fibroblasts (AF1 and AF2), mesothelial cells, pericytes, and smooth muscle cells (SMCs). G. Expression of FAP1 is shown in the 23 subsets. P values were determined using (C, D and E) 2-way ANOVA (*$P < 0.05$) with Tukey's post-hoc test. The data are presented as (C, D and E) the means ± SDs.

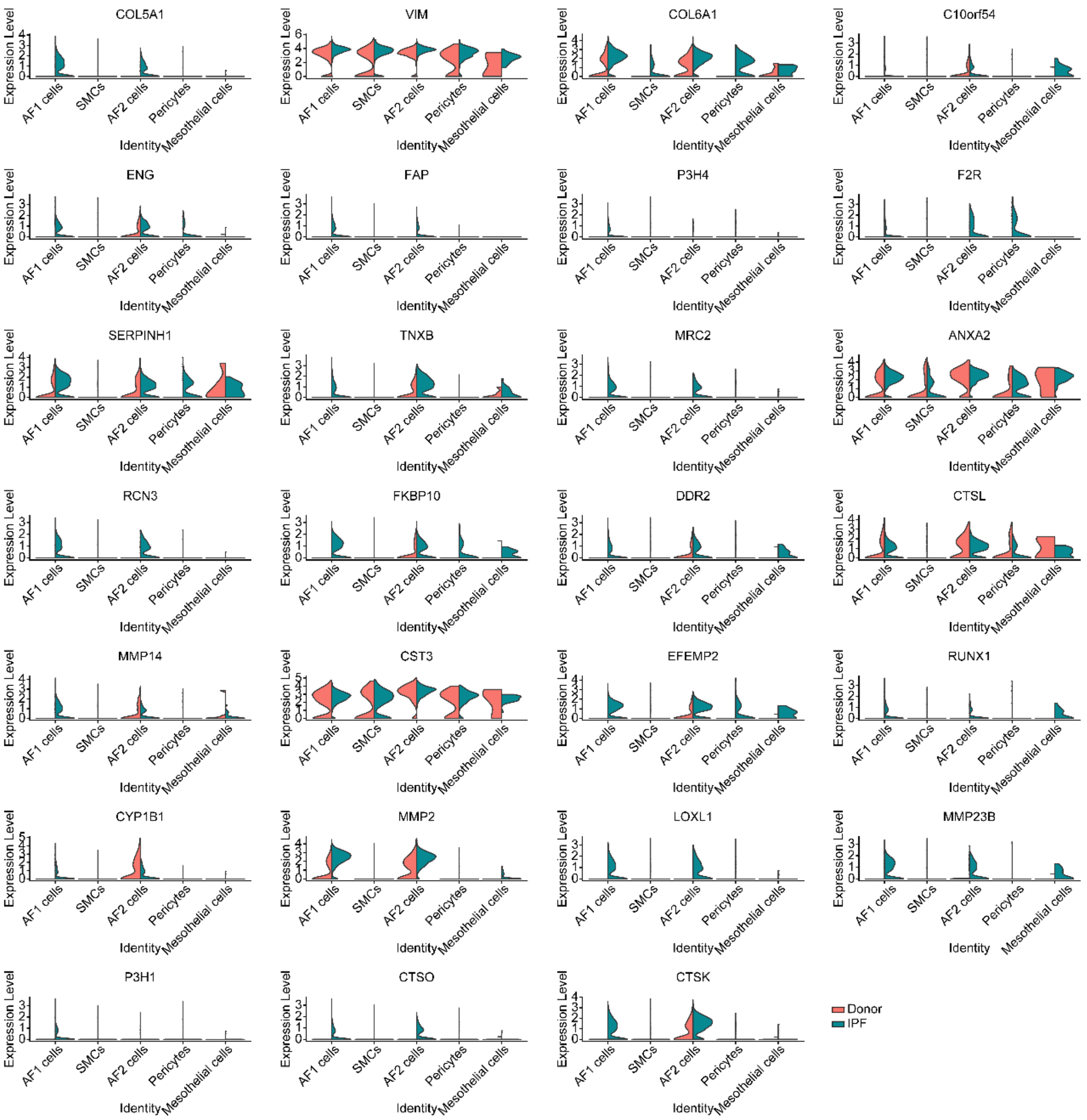

Figure S2. The expression of the 27 eligible genes in AF1, AF2, Mesothelial cells, CAP1, and SMCs.

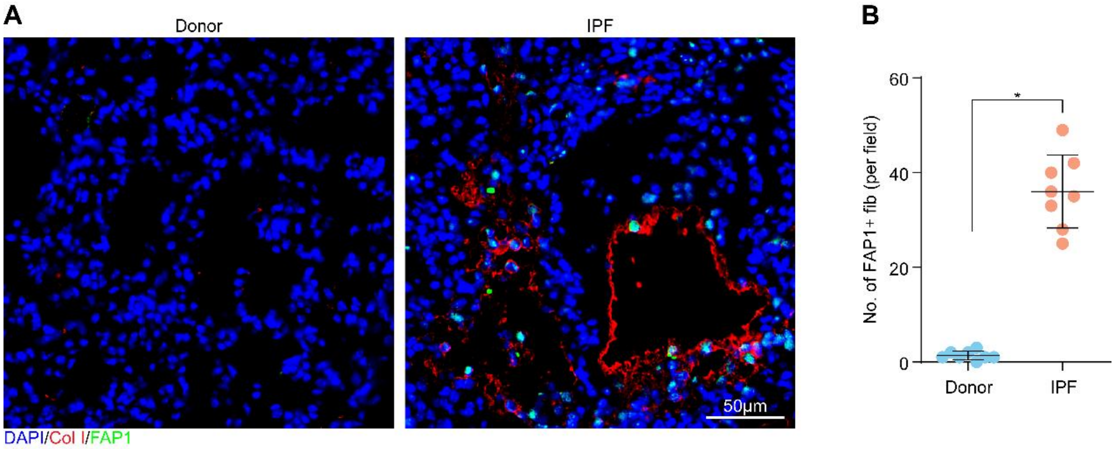

Figrue S3. The FAP1+ AF significantly increased in IPF. (A) Representative fluorescence staining of Col I+FAP1+ cells in IPF. (B) Quantification of Col I+FAP1+ cells were presented as a bar chart. P values were determined using (B) 2-tailed Student's t test (*$P < 0.05$, n.s., not significant). Data are presented as (B) the mean ± SD. $n = 8$ (B).

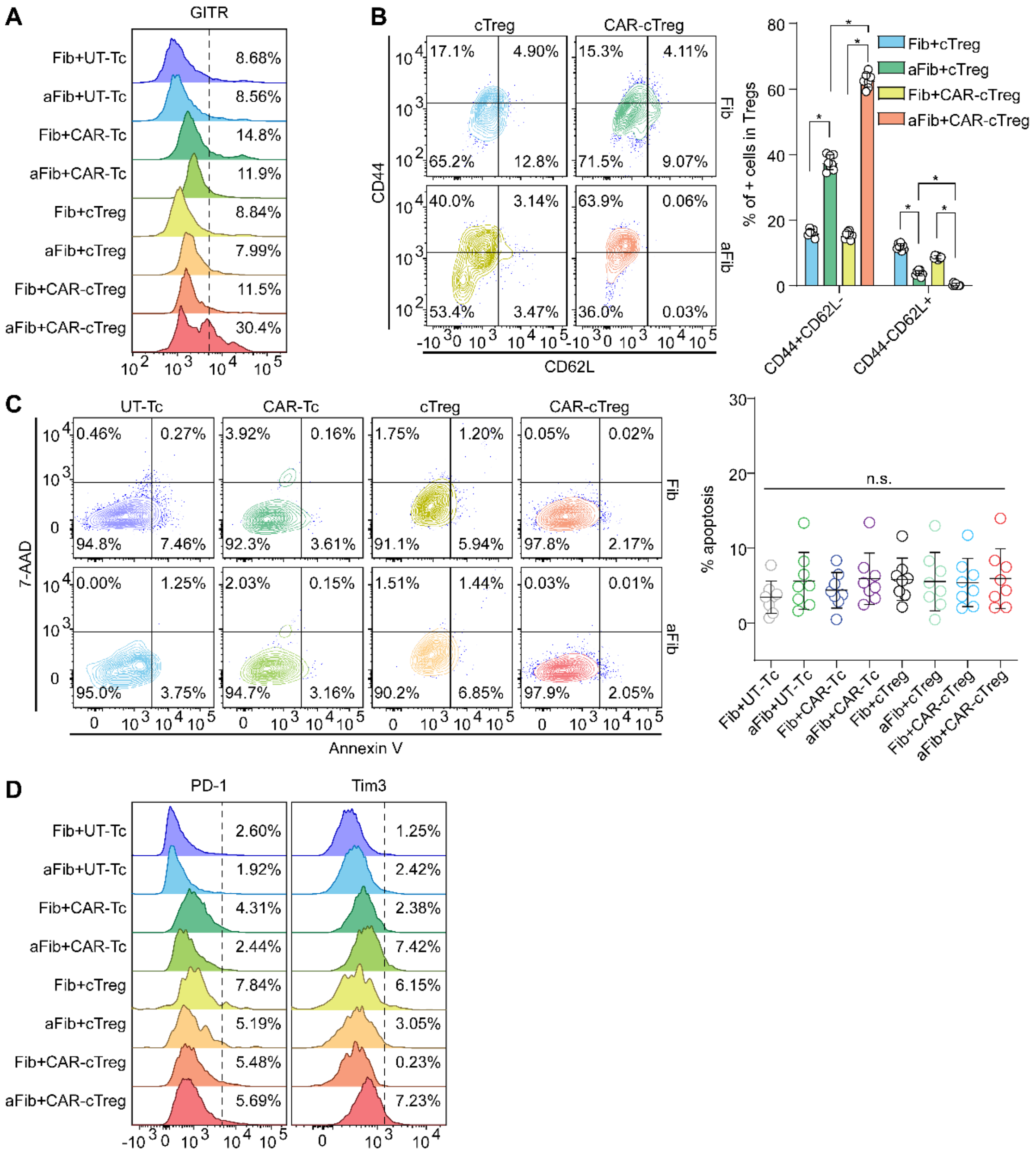

Figure S4. Characterization of FAP1-specific CAR-cTreg cells. (A) The expression of Treg activation-associated markers (GITR) was measured by flow cytometry after T cells were cocultured with lung fibroblasts (Fibs). (B) The ratio of CD44+CD62L- and CD44-CD62L+ Treg were evaluated as representative density plots and a bar chart. (C) Apoptotic ratio of T cells after co-cultured with T cells. (D) The exhaustion of T cells was measured by the surface markers PD-1 and Tim3. P values were determined using (B) 2-way ANOVA with Tukey's post-hoc test or (C) 1-way ANOVA with Tukey's post-hoc test (*P < 0.05, n.s., not significant). Data are presented as (B and C) the mean ± SD. *n* = 8 (B), *n* = 8 (C). CAR-cTreg, chimeric antigen receptor cytotoxic effector regulatory T cell; cTreg, cytotoxic effector regulatory T cell; CAR-Tc, chimeric antigen receptor cytotoxic T cell; UT-Tc, untransduced cytotoxic T cell.

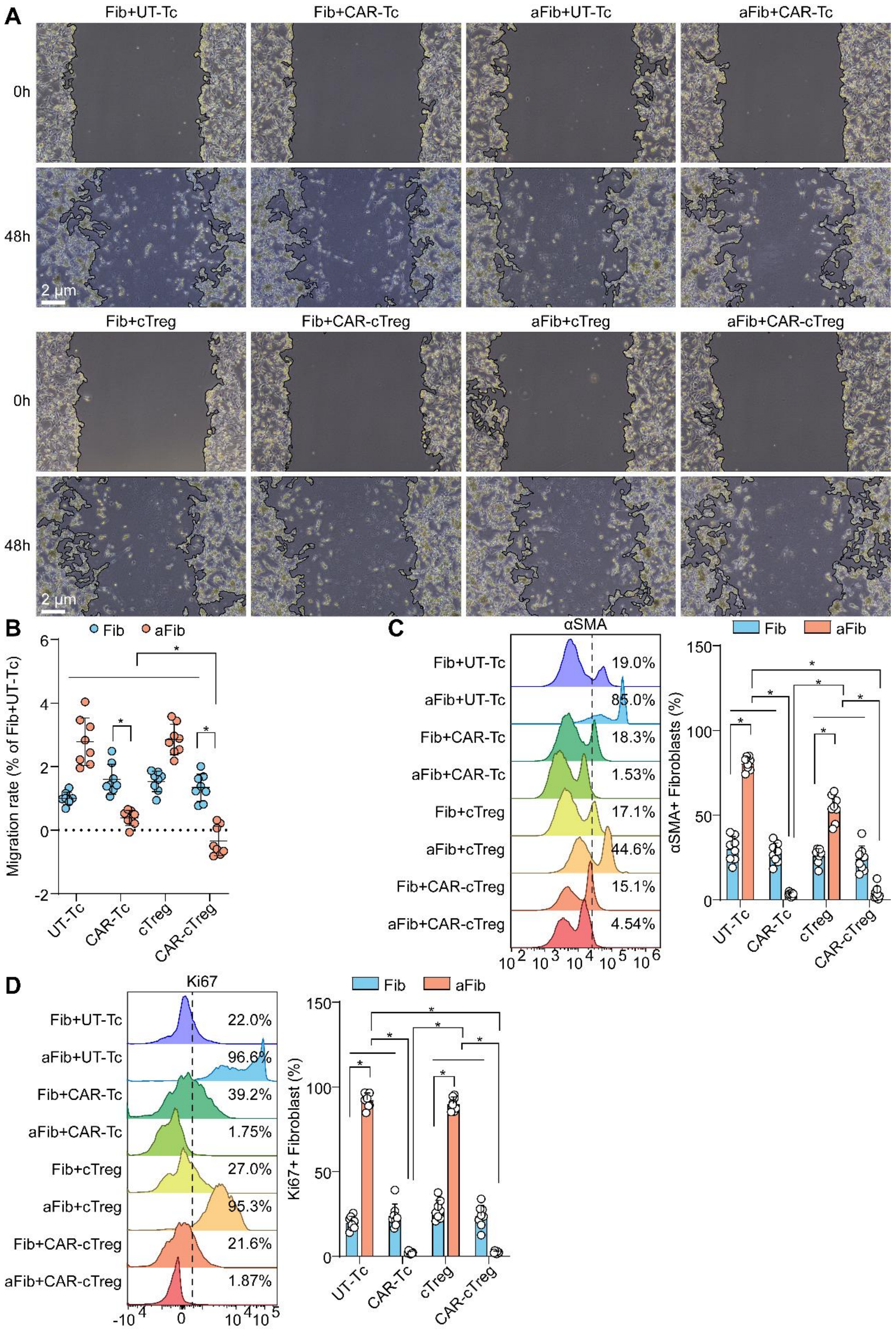

Figure S5. The in vitro function evaluation of lung fibroblasts following co-cultured with Tregs. (A) The wound healing assay was presented ass the presentative microscope images. (B) the bar chart of the wound healing assay. (C) The activation levels (αSMA) of lung fibroblasts was evaluated by flow cytometry. (D) The proliferation marker

(Ki67) of lung fibroblasts was evaluated by flow cytometry. P values were determined using (B, C and D) 2-way ANOVA with Tukey's post-hoc test (*P < 0.05, n.s., not significant). Data are presented as (B, C and D) the mean ± SD. *n* = 8 (B), *n* = 8 (C), *n* = 8 (D). CAR-cTreg, chimeric antigen receptor cytotoxic effector regulatory T cell; cTreg, cytotoxic effector regulatory T cell; CAR-Tc, chimeric antigen receptor cytotoxic T cell; UT-Tc, untransduced cytotoxic T cell.

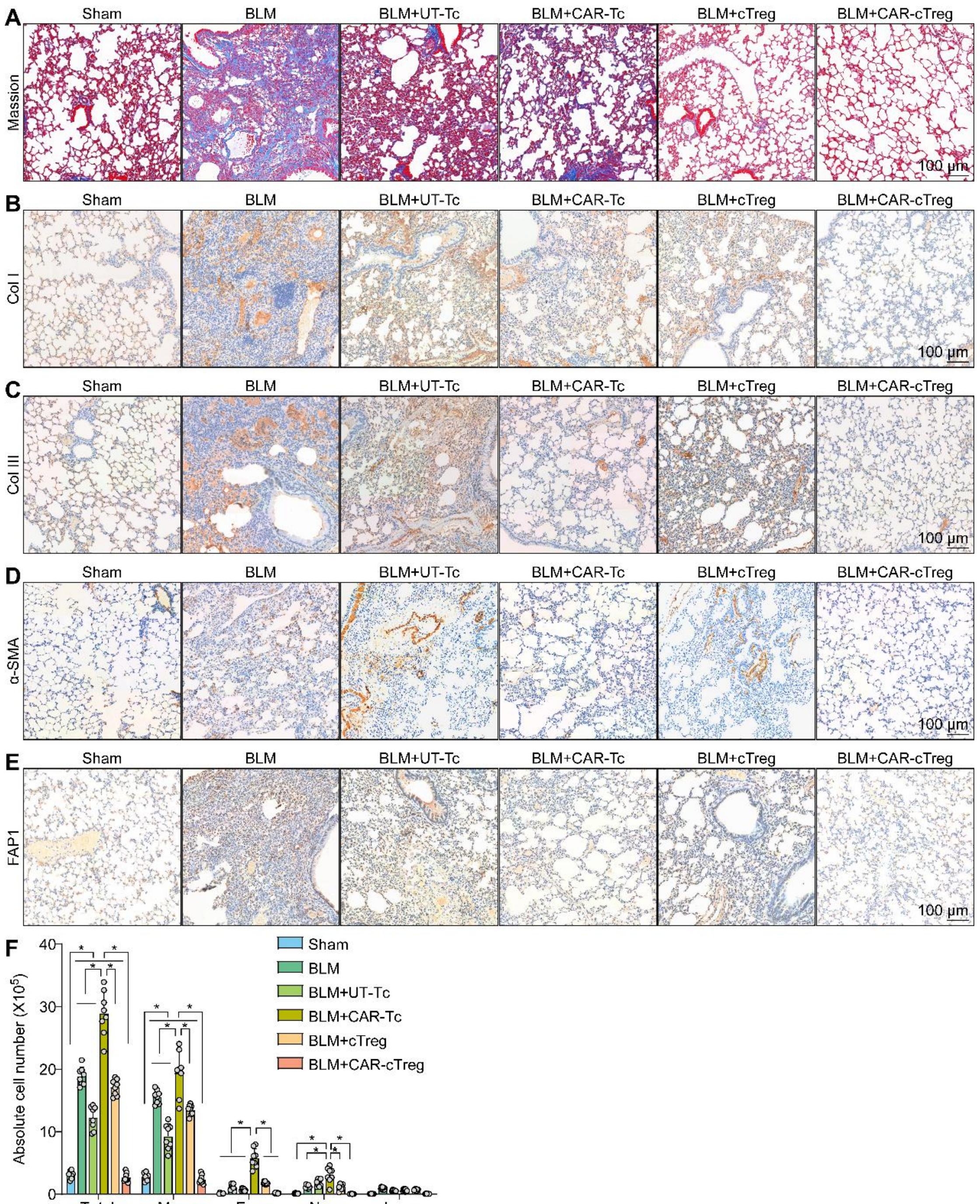

Figure S6. The expression of the fibrotic signature proteins and inflammation were evaluated after CAR-cTreg infusion. (A) Presentative stains of trichrome Masson. (B) Presentative stains of collagen type 1 alpha 1 (Col I). (C) Presentative stains of collagen type 3 alpha 1 (Col III). (D) Presentative stains of α smooth muscle actin (αSMA). (E) Presentative stains of fibroblast activation protein 1 (FAP1). (F) Quantification of bronchoalveolar lavage fluid (BALF) cells after CAR-cTreg treatment (n=8 each group). P values were determined using (F) 2-way ANOVA with Tukey's post-hoc test (*$P < 0.05$, n.s., not significant). Data are presented as (F) the mean ± SD. n = 16 (F). CAR-cTreg, chimeric antigen receptor cytotoxic effector regulatory T cell; cTreg, cytotoxic effector regulatory T cell; CAR-Tc, chimeric antigen receptor cytotoxic T cell;

UT-Tc, untransduced cytotoxic T cell.

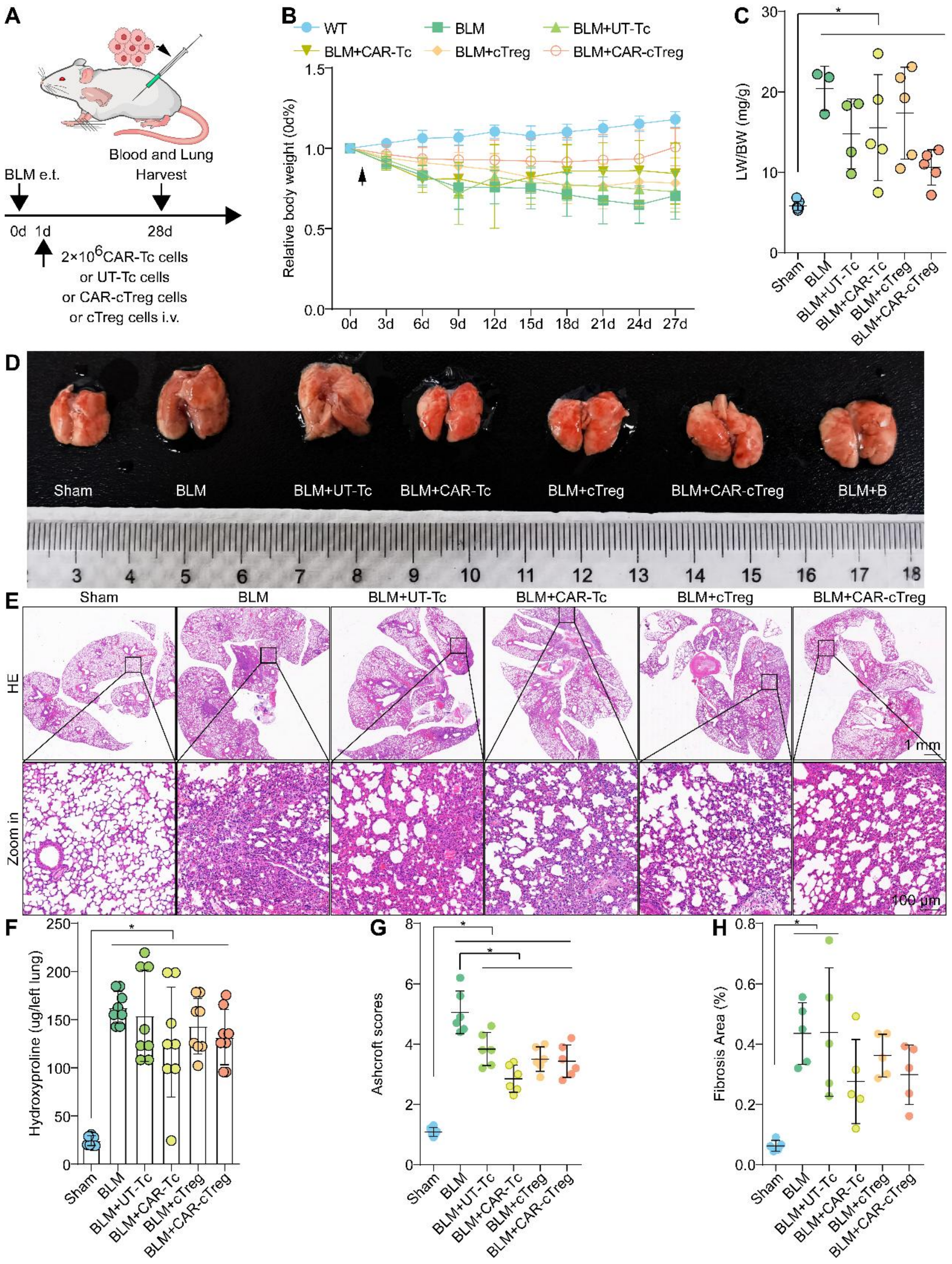

Figure S7. Antifibrotic effect of FAP1 CAR-cTregs against BLM-induced lung fibrosis at day 1 after BLM infusion. (A) Groups of 6–8 weeks C57BL/6J mice were intratracheal injected with 2.5 U/kg BLM, and then adoptively intravenously (IV) with either un-transduced (UT-Tc), or CAR-Tc or un-transduced (cTreg), or CAR-cTreg cells (2 × 106 total T cells) on days 1 post BLM infusion. (B) The relative body weight (BW) of mice changed after T cells infusion (n=7 each group). (C) The ratio of lung weight (LW) to BW was also determined following T cells treatment in sham (n=7), BLM (n=3), BLM+UT-Tc (n=4), BLM+CAR-Tc (n=5), BLM+cTreg (n=5), and BLM+CAR-

cTreg (n=5). (D) The images of lung from all the groups were presented. (E) HE stains of Lung tissue after a regime of T cells infusion. (F) Hydroxyproline concentrations measured in the presence or absence of CAR-cTreg infusion (n=4 each group). (G) Inflammation was quantified by Ashcroft scores (n=6 each group). (H) Fibrosis area was analyzed as a bar chart (n=5). P values were determined using (B) 2-way ANOVA with Tukey's post-hoc test or (C, F, G and H) 1-way ANOVA with Tukey's post-hoc test (*$P < 0.05$, n.s., not significant). Data are presented as (B, C, F, G and H) the mean ± SD. $n = 7$ (B), $n = 3$-7 (C), $n = 8$ (F), $n = 6$ (G), $n = 5$ (H). CAR-cTreg, chimeric antigen receptor cytotoxic effector regulatory T cell; cTreg, cytotoxic effector regulatory T cell; CAR-Tc, chimeric antigen receptor cytotoxic T cell; UT-Tc, untransduced cytotoxic T cell.

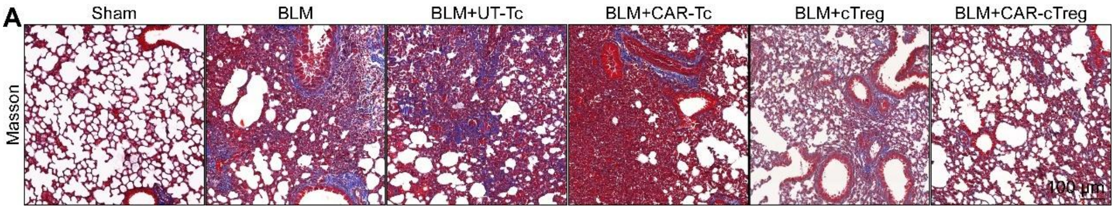

Figure S8. Presentative stains of trichrome Masson after CAR-cTreg treatment at day 1. (A) Presentative stains of trichrome Masson. CAR-cTreg, chimeric antigen receptor cytotoxic effector regulatory T cell; cTreg, cytotoxic effector regulatory T cell; CAR-Tc, chimeric antigen receptor cytotoxic T cell; UT-Tc, untransduced cytotoxic T cell.

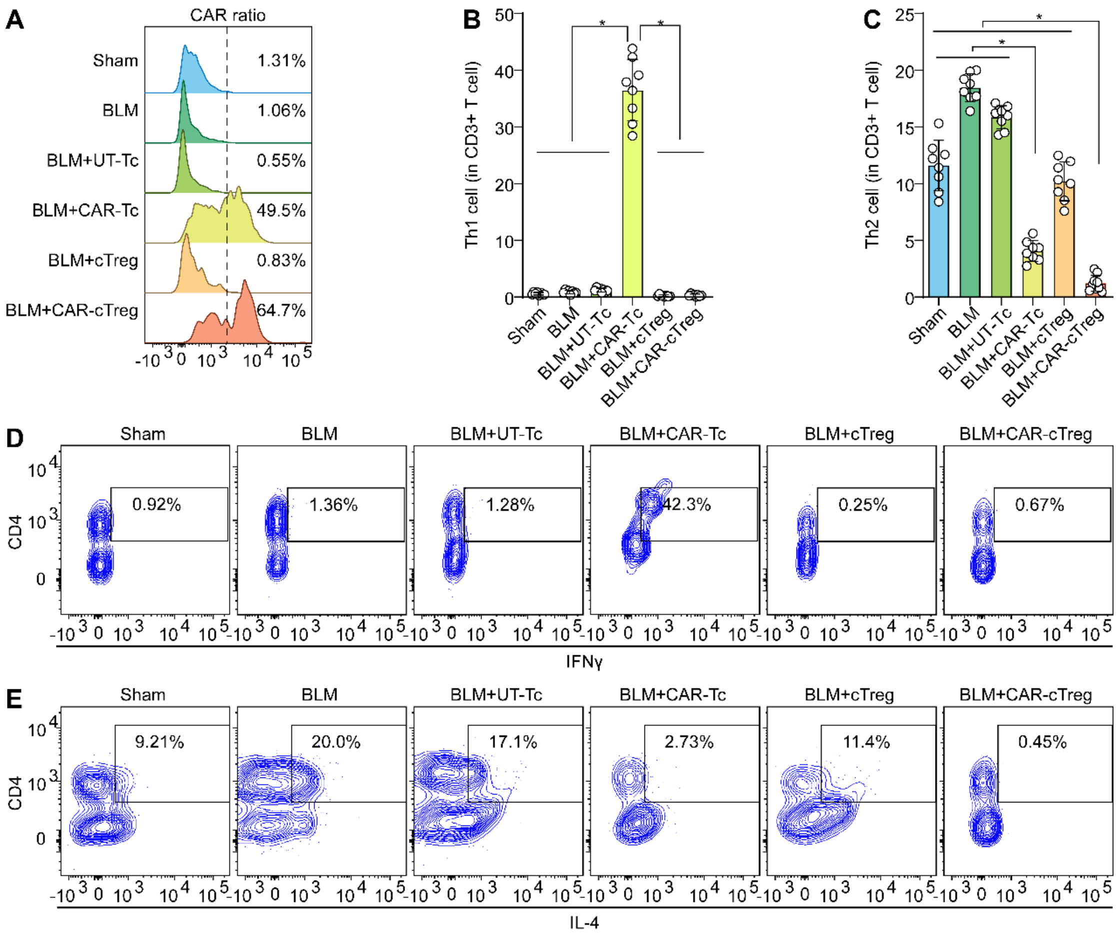

Figrue S9. Changes in the differentiation of T cell from lung tissue on cell infusion 14d post BLM administration. (A) The CAR-cTreg ratio in the infiltrative CD3+ T cells after T cell infusion. (B) Th1 subsets was quantified by a bar chart in lung tissue 2 weeks post T cell infusion (n=3 each group). (C) Th2 subsets was quantified by a bar chart in lung tissue 2 weeks post T cell infusion in sham (n=3), BLM (n=3), BLM+UT-Tc (n=3), BLM+CAR-Tc (n=3), BLM+cTreg (n=3), and BLM+CAR-cTreg (n=4). (D) Th1 subsets are shown as representative density plots in lung tissue 2 weeks post T cell infusion. (E) Th2 subsets are shown as representative density plots in lung tissue 2 weeks post T cell infusion. P values were determined using (B and C) 1-way ANOVA with Tukey's post-hoc test (*P < 0.05, n.s., not significant). Data are presented as (B and C) the mean ± SD. n = 8 (B), n = 8-9 (C). CAR-cTreg, chimeric antigen receptor cytotoxic effector regulatory T cell; cTreg, cytotoxic effector regulatory T cell; CAR-Tc, chimeric antigen receptor cytotoxic T cell; UT-Tc, untransduced cytotoxic T cell.

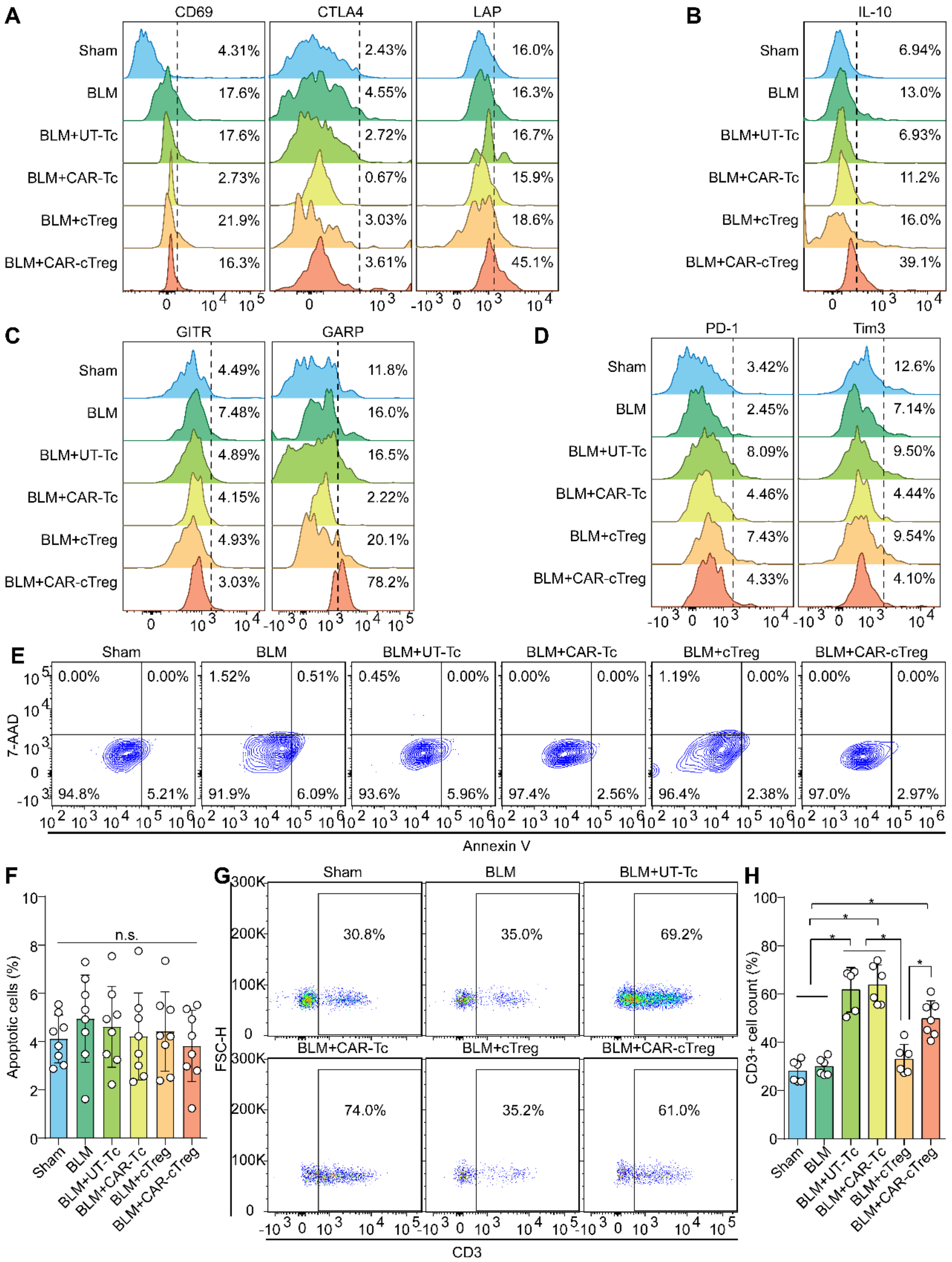

Figure S10. Changes in the pharmacokinetics of CAR-cTregs in peripheral blood. (A) The expression of activation markers (CD69, CTLA4, and LAP) was measured by flow cytometry after T cell infusion. (B) The secretion of IL-10 was evaluated by intracellular flow cytometry following T cell infusion. (C) The expression of Treg activation associated marker (GITR and GARP) were measured by flow cytometry after T cell infusion. (D) The expression of exhaustion markers (PD-1 and Tim3) was measured by flow cytometry after T cell infusion. (E) The apoptosis of T cell was present as representative density plots in lung 2 weeks post T cell infusion. (F) The bar

chart of the apoptosis of T cell in lung 2 weeks post T cell infusion (n=3 each group). (G) The infiltration of CD3+ T cells was evaluated as representative density plots. (H) The bar chart of the infiltration of CD3+ T cells in sham (n=6), BLM (n=6), BLM+UT-Tc (n=5), BLM+CAR-Tc (n=6), BLM+cTreg (n=6), and BLM+CAR-cTreg (n=7). P values were determined using (F and H) 1-way ANOVA with Tukey's post-hoc test (*P < 0.05, n.s., not significant). Data are presented as (F and H) the mean ± SD. n = 8 (F), n = 5-7 (H). CAR-cTreg, chimeric antigen receptor cytotoxic effector regulatory T cell; cTreg, cytotoxic effector regulatory T cell; CAR-Tc, chimeric antigen receptor cytotoxic T cell; UT-Tc, untransduced cytotoxic T cell.

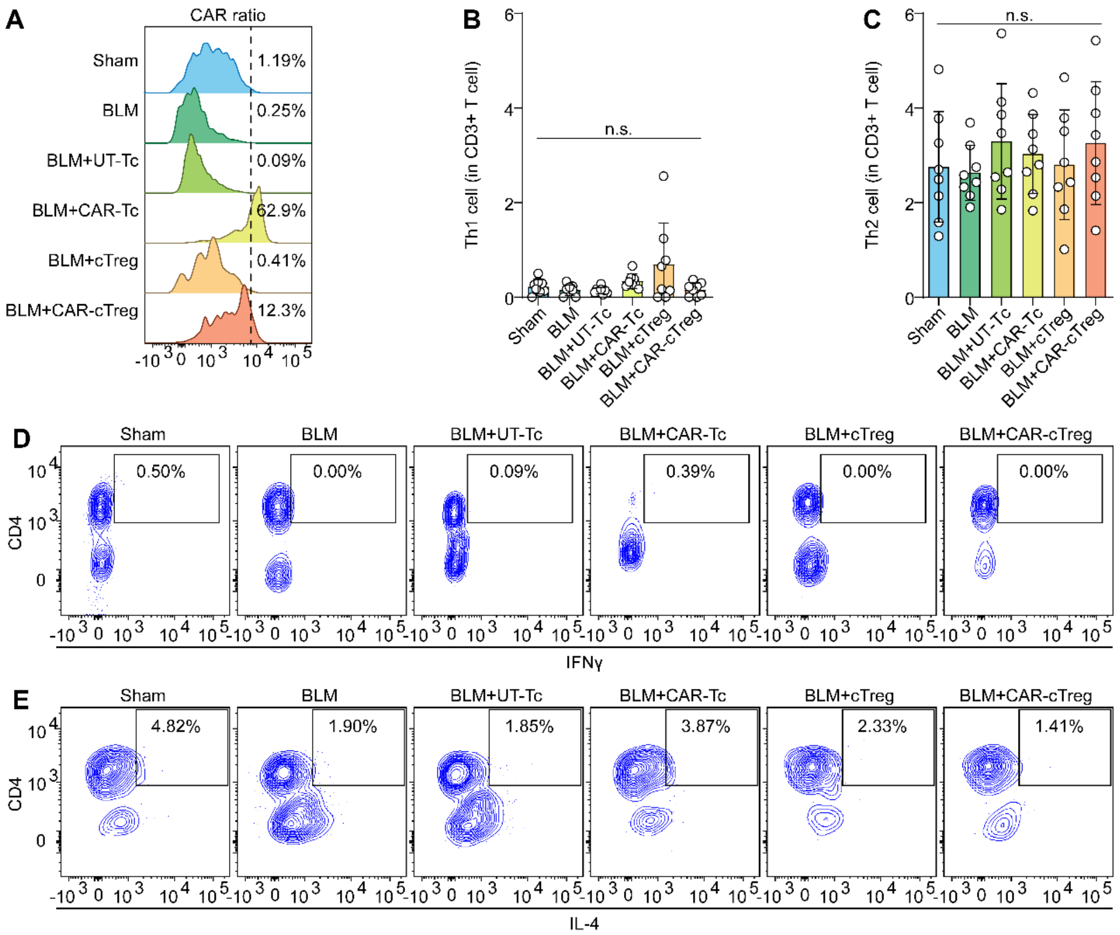

Figure S11. Changes in the differentiation of T cell from lung tissue on cell infusion 1d post BLM administration. (A) The CAR-cTreg ratio in the infiltrative CD3+ T cells after T cell infusion. (B) Th1 subsets was quantified by a bar chart in lung tissue 2 weeks post T cell infusion (n=3 each group). (C) Th2 subsets was quantified by a bar chart in lung tissue 2 weeks post T cell infusion (n=3 each group). (D) Th1 subsets are shown as representative density plots in lung tissue 2 weeks post T cell infusion. (E) Th2 subsets are shown as representative density plots in lung tissue 2 weeks post T cell infusion. P values were determined using (B and C) 1-way ANOVA with Tukey's post-hoc test (*$P < 0.05$, n.s., not significant). Data are presented as (B and C) the mean ± SD. n = 8 (B), n = 8 (C). CAR-cTreg, chimeric antigen receptor cytotoxic effector regulatory T cell; cTreg, cytotoxic effector regulatory T cell; CAR-Tc, chimeric antigen receptor cytotoxic T cell; UT-Tc, untransduced cytotoxic T cell.

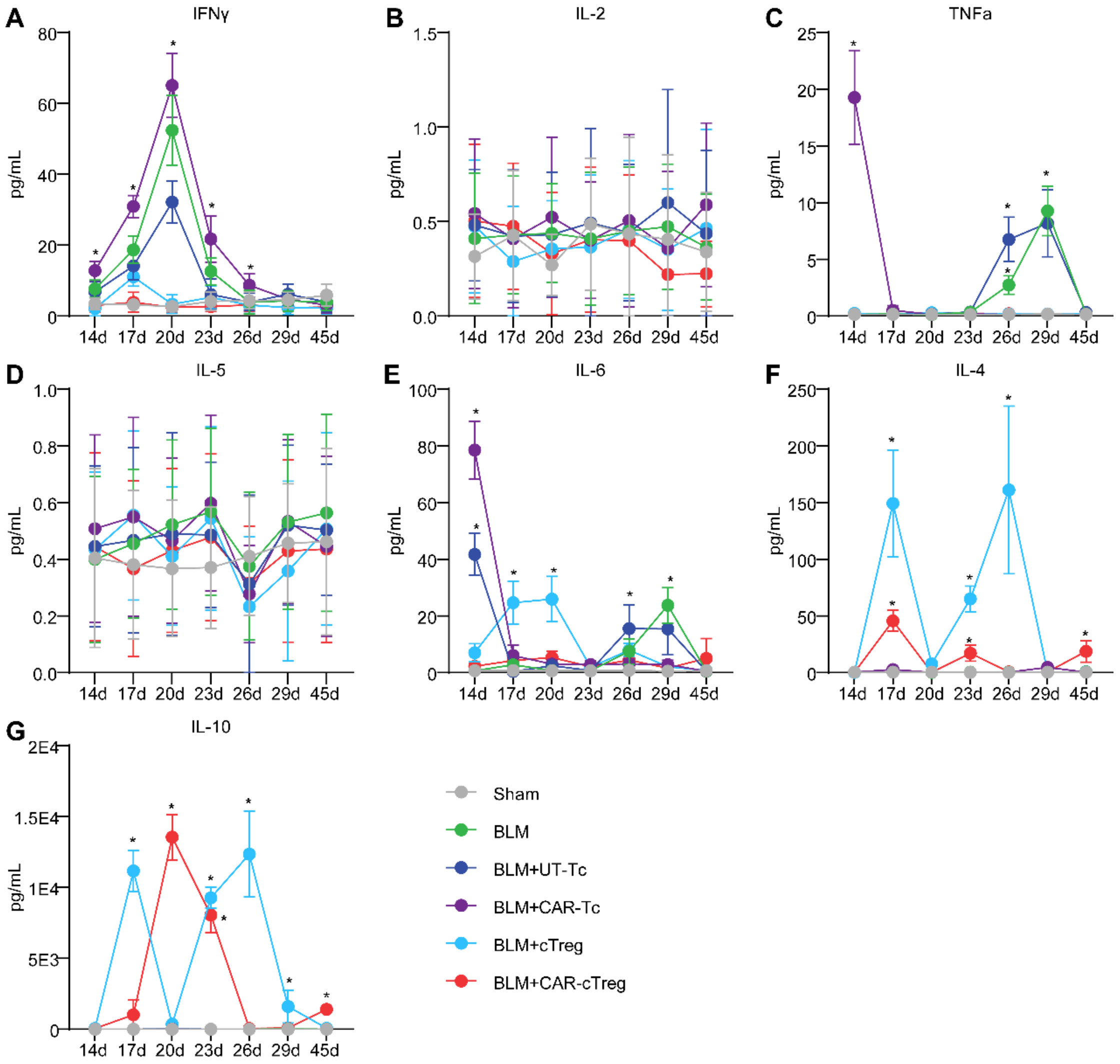

Figure S12. The production of multiple cytokines was evaluated by cytometric bead assay in peripheral blood following CAR-cTreg treatment. A-G. Cytometric bead assay was applied to evaluate the secretion of IFNγ (A, n=3 each group), TNFα (B, n=4 each group), IL-2 (C, n=4 each group), IL-5 (D, n=4 each group), IL-6 (E, n=4 each group), IL-4 (F, n=4 each group), and IL-10 (G, n=4 each group) after CAR-cTreg treatment. P values were determined using (A, B, C, D, E, F and G) 2-way ANOVA with Tukey's post-hoc test (*P < 0.05, n.s., not significant). Data are presented as (A, B, C, D, E, F and G) the mean ± SD. *n* = 11 (A), *n* = 12 (B), *n* = 12 (C), *n* = 12 (D), *n* = 12 (E), *n* = 12 (F), *n* = 12 (G). CAR-cTreg, chimeric antigen receptor cytotoxic effector regulatory T cell; cTreg, cytotoxic effector regulatory T cell; CAR-Tc, chimeric antigen receptor cytotoxic T cell; UT-Tc, untransduced cytotoxic T cell.

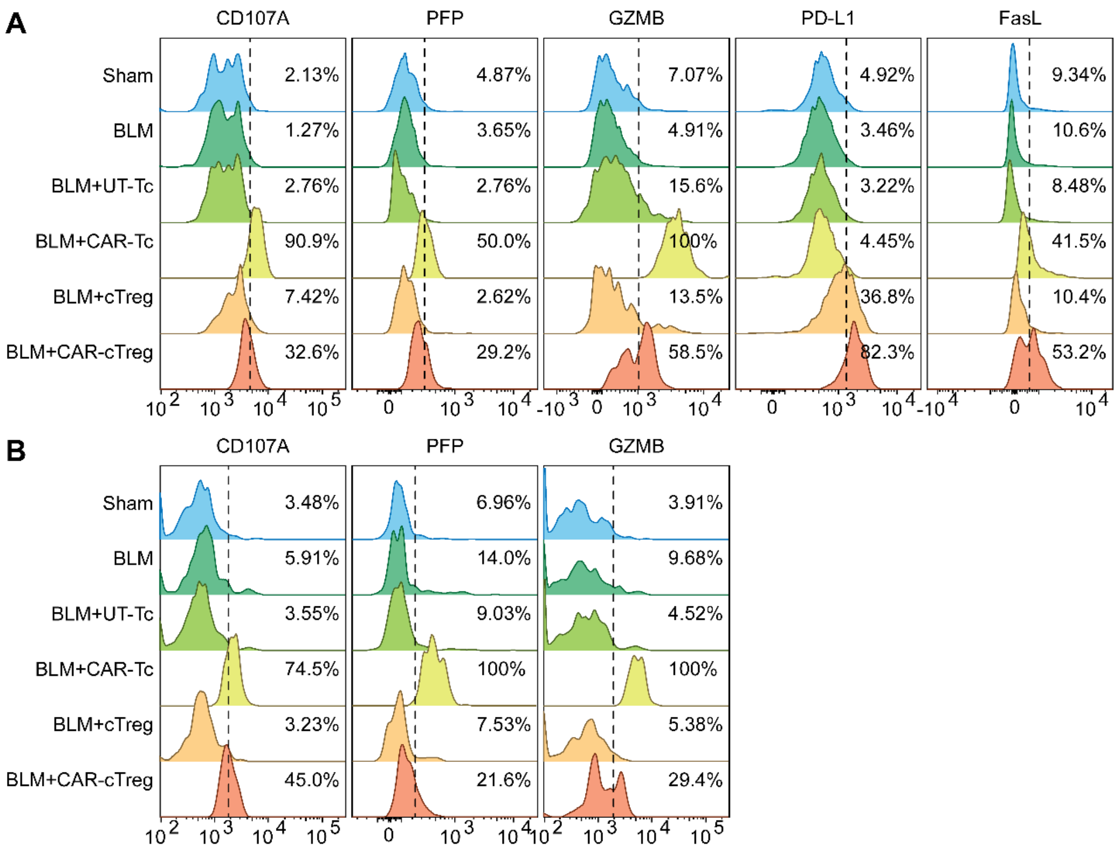

Figure S13. The expression of CD107A, PFP, GZMB, PD-L1 and FasL in T cells following CAR-cTreg infusion. (A) The expression of CD107A, PFP, GZMB, PD-L1 and FasL in T cells from lung following CAR-cTreg infusion. (B) The expression of CD107A, PFP, and GZMB in T cells from blood following CAR-cTreg infusion. CAR-cTreg, chimeric antigen receptor cytotoxic effector regulatory T cell; cTreg, cytotoxic effector regulatory T cell; CAR-Tc, chimeric antigen receptor cytotoxic T cell; UT-Tc, untransduced cytotoxic T cell.

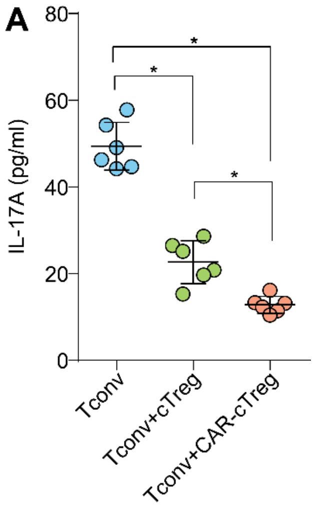

Figure S14. CAR-cTreg cells inhibit the secretion of IL-17a. (A) Normalized in vitro suppression of IL-17a production when cocultured with cTreg, CAR-cTreg or FAP1-stimulated CAR-cTreg. P values were determined using (A) 1-way ANOVA with Tukey's post-hoc test (*P < 0.05, n.s., not significant). Data are presented as (A) the mean ± SD. n = 6 (A). CAR-cTreg, chimeric antigen receptor cytotoxic effector regulatory T cell; cTreg, cytotoxic effector regulatory T cell; CAR-Tc, chimeric antigen receptor cytotoxic T cell; UT-Tc, untransduced cytotoxic T cell.

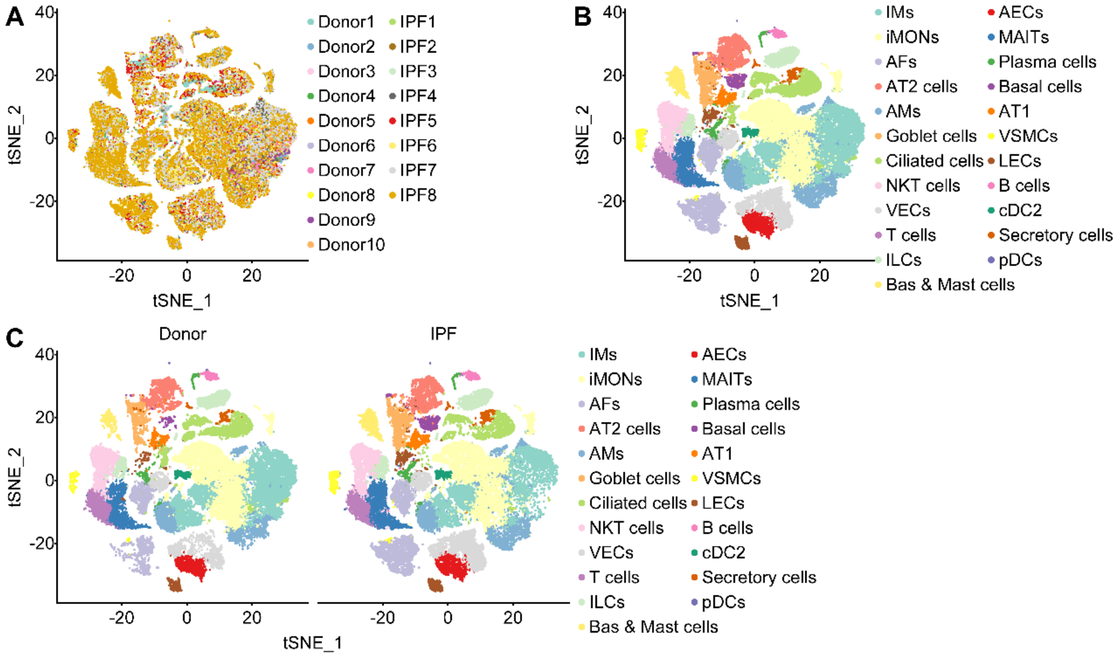

Figure S15. The t-SNE of cell composition in IPF. A. The t-SNE of single-cell RNA sequencing in HDs (n=10) and patients with IPF patients (n=8). B. The t-SNE analysis of cell composition in single-cell RNA sequencing data. C. The t-SNE analysis of cell composition in HDs (n=10) and IPF patients (n=8).

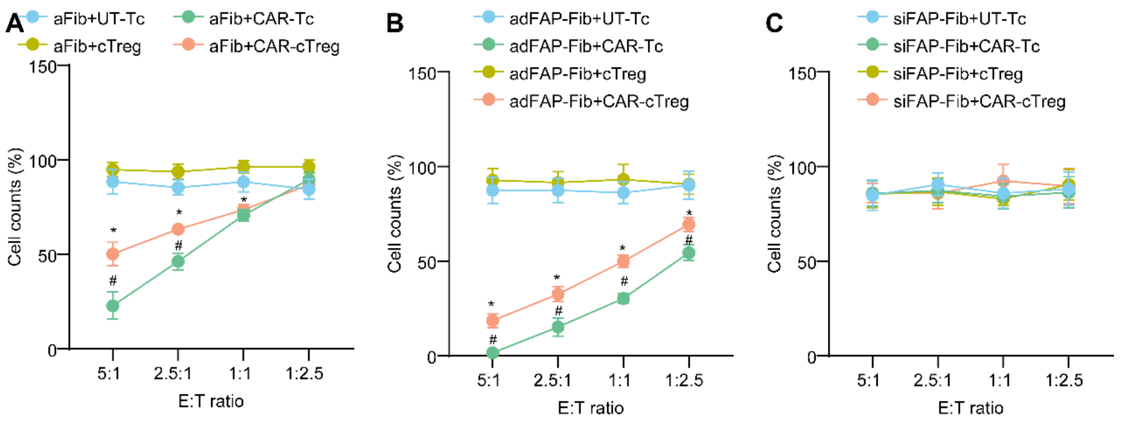

Figure S16. The specific antifibrotic effect of CAR-Tcs and CAR-cTregs determined by cell counter. The specific antifibrotic effect on aFibs (A), Fib with FAP overexpression (adFAP-Fib, B), Fib with FAP knockdown (siFAP-Fib, C) was measured by cell counter in culture media at the indicated E:T ratios. P values were determined using (A, B and C) 2-way ANOVA with Tukey's post-hoc test (*P < 0.05). (A, B) *, P < 0.05 for the comparison to aFib+UT-Tc and aFib+cTreg. #, P < 0.05 for the comparison between aFib+CAR-Tc and aFib+CAR-cTreg. Data are presented as (A, B and C) the mean ± SD. *n* = 10 (A), *n* = 5 (B), *n* = 5 (C). CAR-cTreg, chimeric antigen receptor cytotoxic effector regulatory T cell; cTreg, cytotoxic effector regulatory T cell; CAR-Tc, chimeric antigen receptor cytotoxic T cell; UT-Tc, untransduced cytotoxic T cell.

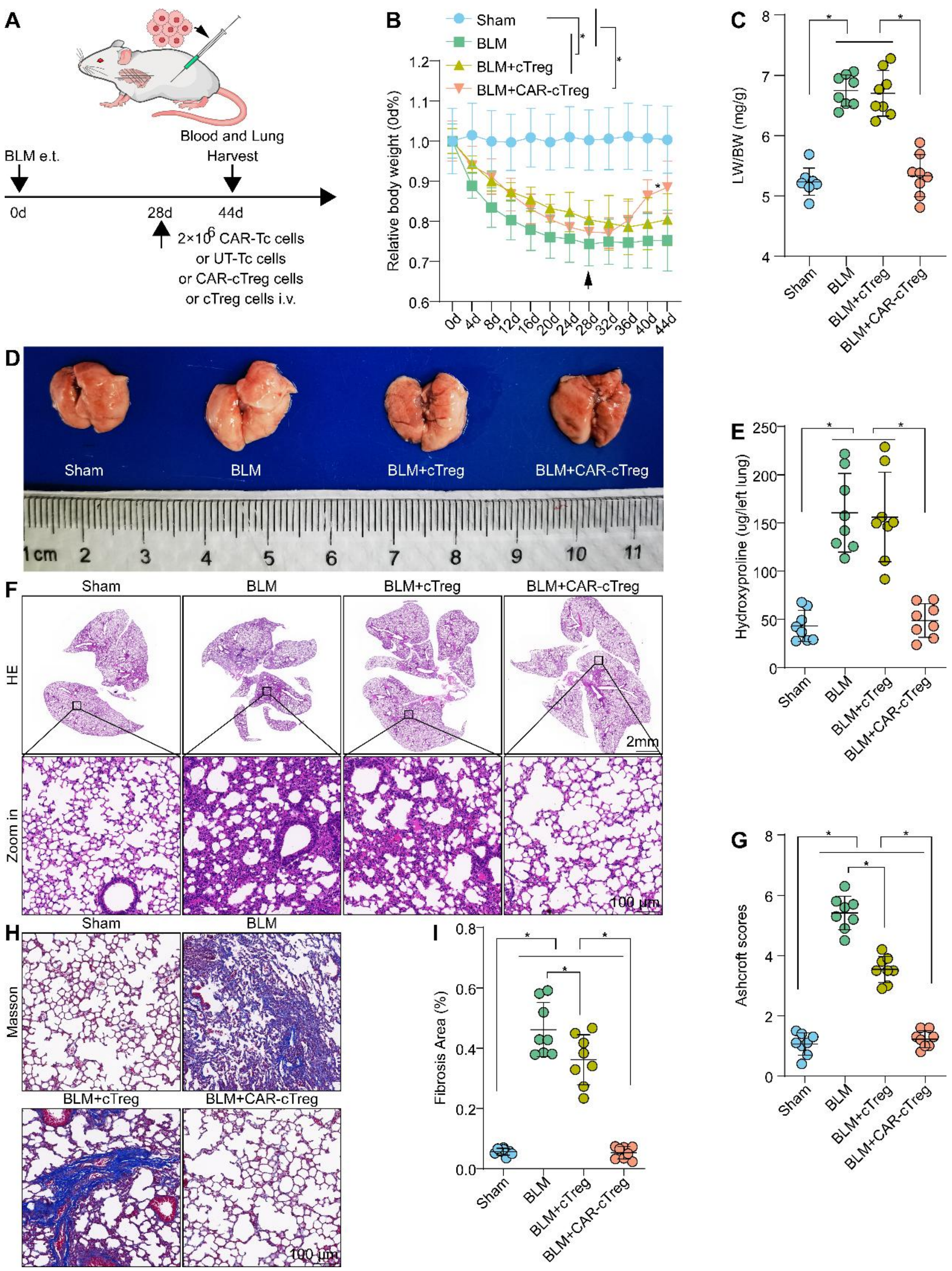

Figure S17. Antifibrotic effect of FAP1 CAR-cTregs against BLM-induced lung fibrosis at day 28 after BLM infusion. (A) Groups of 6–8-week-old C57BL/6J mice (n = 8) were intratracheally injected with 2.5 U/kg BLM and then adoptively intravenously (IV) injected with untransduced Tregs (cTreg), or CAR-cTreg (2 × 106 total T cells) on day 28 post BLM infusion. (B) Changes in the relative body weight (BW) of mice after BLM infusion (n=8 each group). (C) The ratio of lung weight (LW) to BW was also determined following CAR-cTreg treatment (n=8 each group). (D) Images of lungs from all the groups are presented. (E) Hydroxyproline (HYP) concentrations measured

in the presence or absence of CAR-Treg infusion (n=8 each group). (F) H&E staining of lung tissue after T-cell infusion. (G) Inflammation was quantified by Ashcroft scores (n=8 each group). (H) Presentative stains of trichrome Masson. (I) The fibrotic area was analyzed as shown in the bar chart (n=8 each group). P values were determined using (B) 2-way ANOVA with Tukey's post-hoc test or (C, E, G and I) 1-way ANOVA with Tukey's post-hoc test (*$P < 0.05$). (B) *, $P < 0.05$ for the comparison between BLM+CAR-cTreg and all other groups. The data are presented as (B, C, E, G and I) the means ± SDs. $n = 8$ (B), $n = 8$ (C), $n = 8$ (E), $n = 8$ (G), $n = 8$ (I). CAR-cTreg, chimeric antigen receptor cytotoxic effector regulatory T cell; cTreg, cytotoxic effector regulatory T cell.

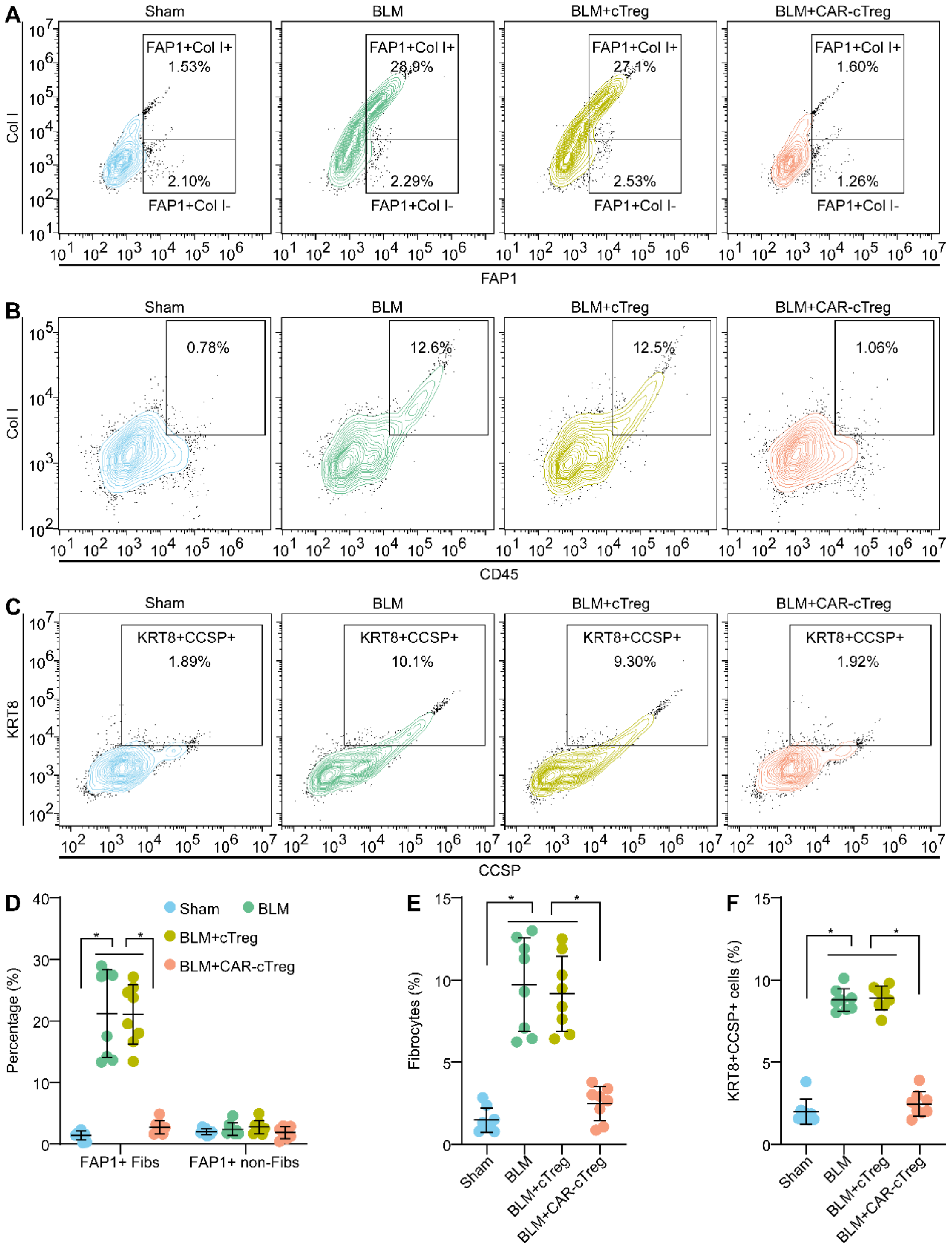

Figure S18. After CAR-cTreg treatment, FAP+ fibroblasts were significantly reduced without a decrease in other FAP+ cell populations, accompanied by a reduction in fibrocytes and KRT8+CCSP+ cells. Representative density plots of Col I+FAP1+ cells (A), CD45+Col I+ cells (fibrocytes, B) and KRT8+CCSP+ cells (C) in lung tissue after T-cell infusion. Quantification of Col I+FAP1+ cells (D, n=8 each group), fibrocytes (E, n=8 each group) and KRT8+CCSP+ cells (F, n=8 each group) is presented as a dot plot. P values were determined using (E and F) 1-way ANOVA and (D) 2-way ANOVA (*$P < 0.05$) with Tukey's post-hoc test. The data are presented as (D, E and F) the means

± SDs. *n* = 8 (D), *n* = 8 (E), *n* = 8 (F). CAR-cTreg, chimeric antigen receptor cytotoxic effector regulatory T cell; cTreg, cytotoxic effector regulatory T cell; CAR-Tc, chimeric antigen receptor cytotoxic T cell; UT-Tc, untransduced cytotoxic T cell.

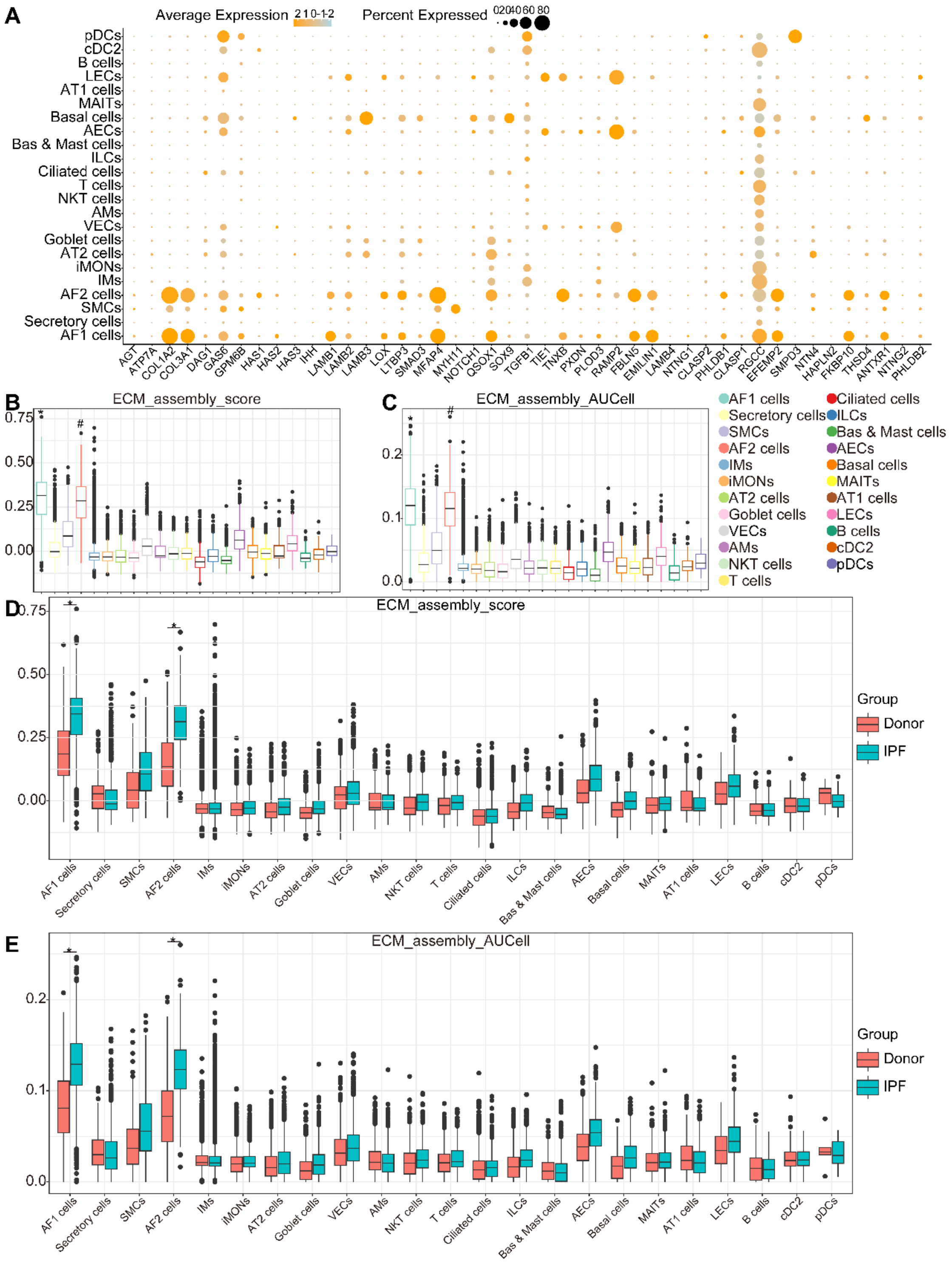

Figure S19. AF1 and AF2 cells presented significantly elevated extracellular matrix assembly activity. (A) The expression levels of genes involved in "GO: 0085029 extracellular matrix assembly" are shown across 23 subsets. The activity score of the extracellular matrix assembly is shown for each subset, which was calculated by the AddModuleScore function (B) and the AUCell function (C). The extracellular matrix assembly score of the 23 subsets is shown for HDs (n=10) and IPF patients (n=8) and was calculated by the AddModuleScore function (D) and the AUCell function (E). (B, C) *, P < 0.05 for comparisons of AF1 cells with all other subgroups except AF2 cells;

#, P < 0.05 for comparisons of AF2 cells with all other subgroups except AF1 cells. P values were determined using (B and C) 1-way ANOVA with Tukey's post-hoc test or (D and E) 2-way ANOVA (*P < 0.05) with Tukey's post-hoc test. The data are presented as (B, C, D and E) the means ± SDs.

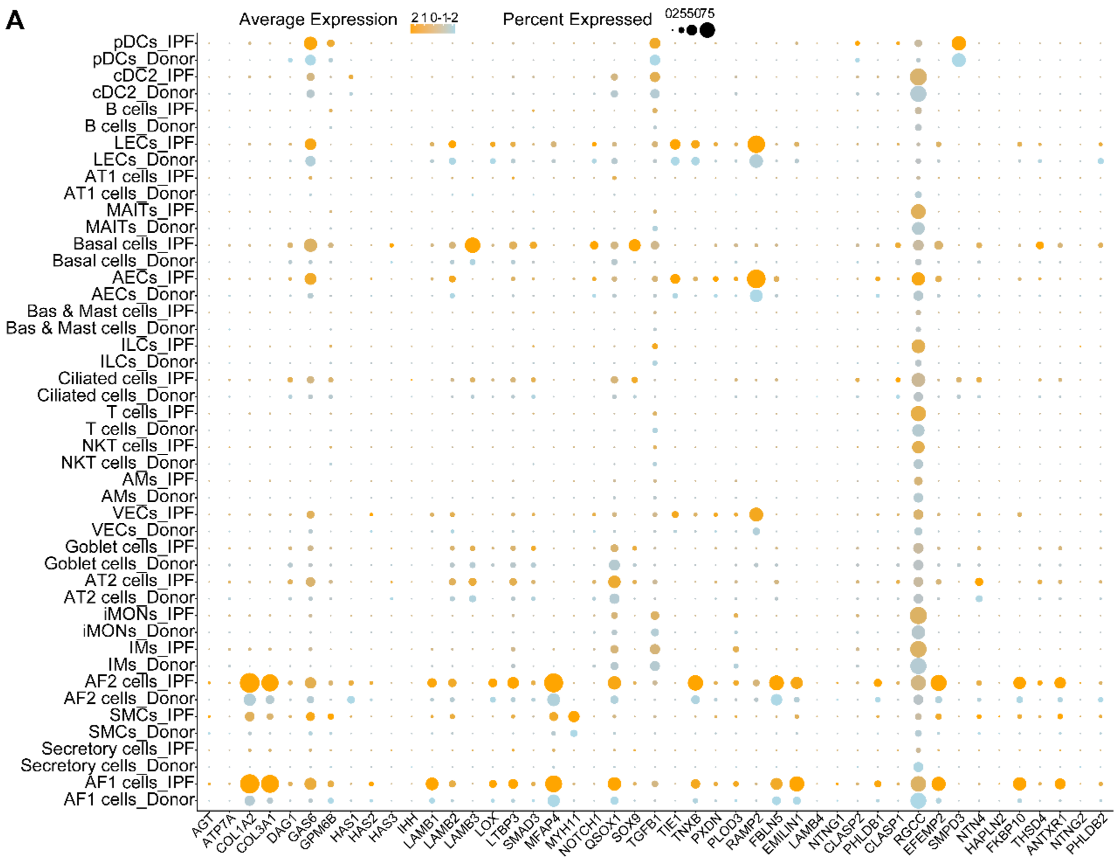

Figure S20. The expression levels of genes involved in extracellular matrix assembly are shown across 23 subsets in both HDs (n=10) and IPF patients (n=8).

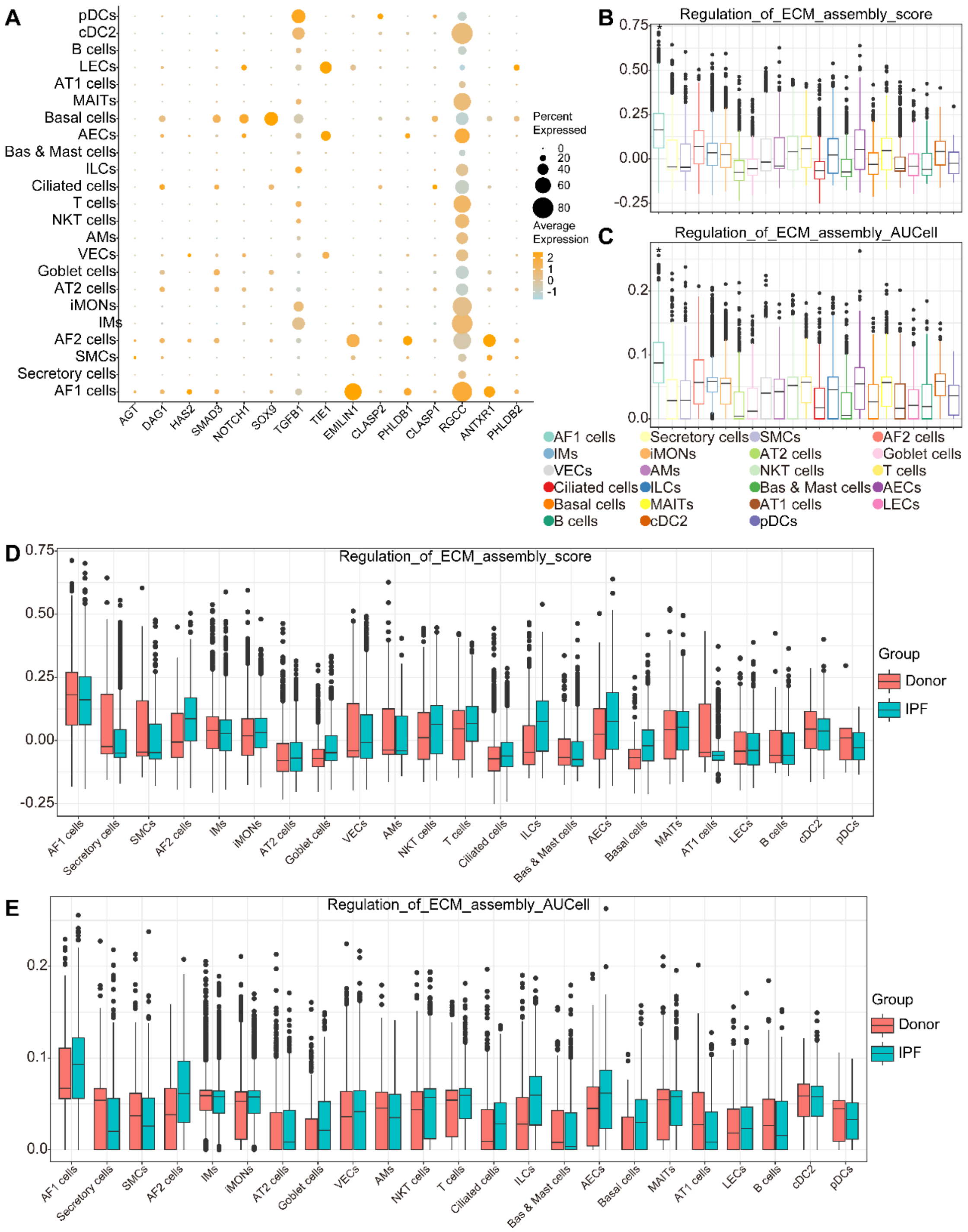

Figure S21. AF1 and AF2 cells exhibited significantly elevated regulatory activity of extracellular matrix assembly. (A) The expression levels of genes involved in "GO: 1901201 regulation of extracellular matrix assembly" are shown across 23 subsets. The regulatory activity score of the extracellular matrix assembly is shown for each subset, which was calculated by the AddModuleScore function (B) and the AUCell function (C). The regulatory activity score of the extracellular matrix assembly is shown across 23 subsets in HDs (n=10) and IPF patients (n=8) and was calculated via the AddModuleScore function (D) and the AUCell function (E). (B, C) *, P < 0.05 for comparisons of AF1 cells with all other subgroups except AF2 cells; #, P < 0.05 for

comparisons of AF2 cells with all other subgroups except AF1 cells. P values were determined using (B and C) 1-way ANOVA with Tukey's post-hoc test or (D and E) 2-way ANOVA (*$P < 0.05$) with Tukey's post-hoc test. The data are presented as (B, C, D and E) the means ± SDs.

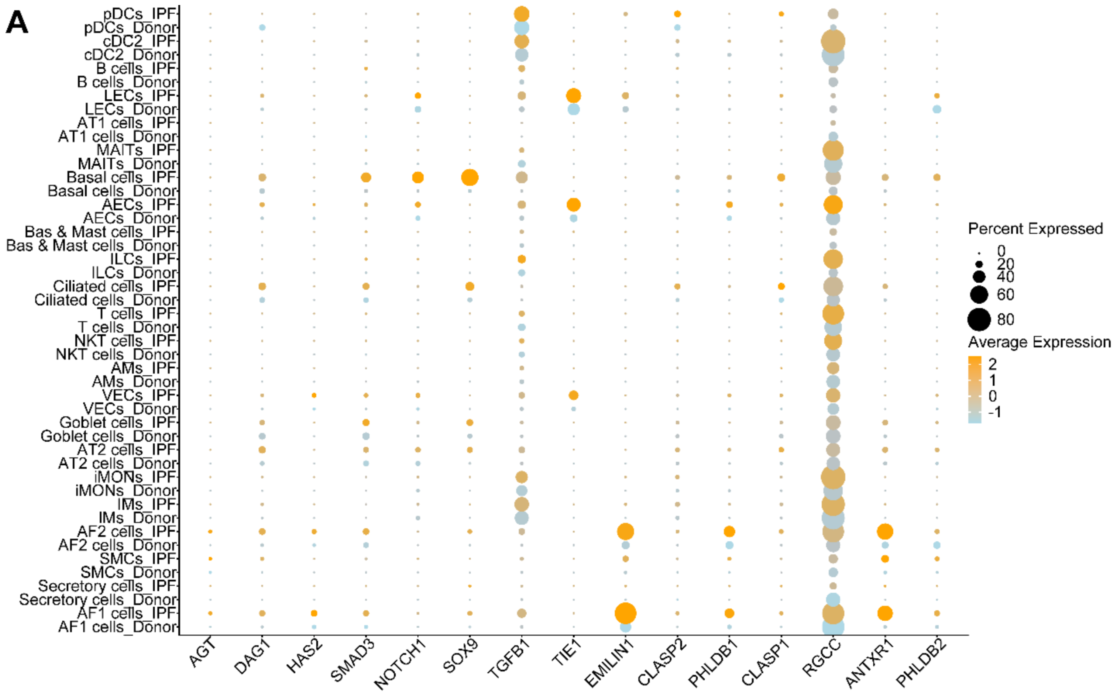

Figure S22. The expression levels of genes involved in the regulation of extracellular matrix assembly are shown across 23 subsets in both HDs (n=10) and IPF patients (n=8).

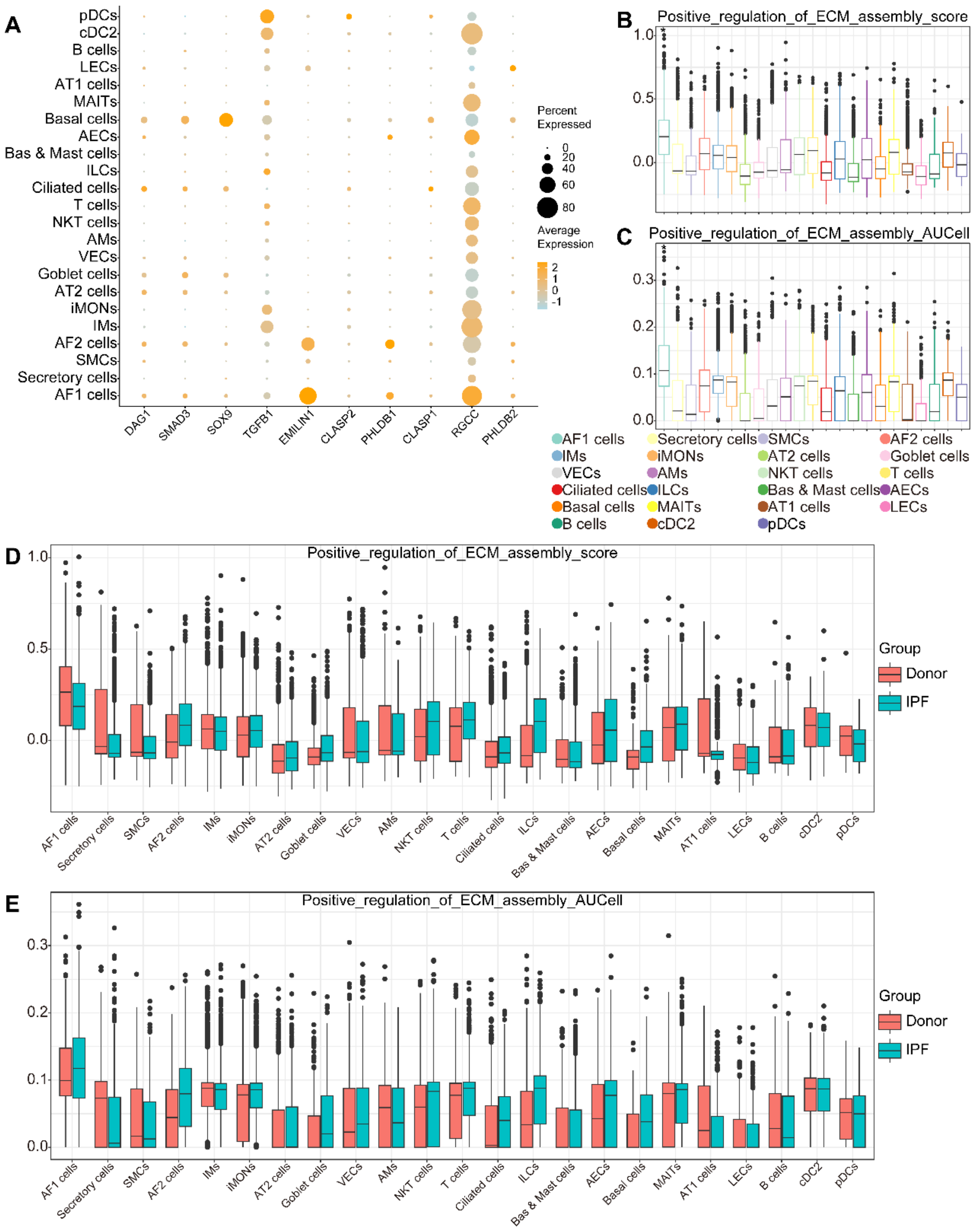

Figure S23. AF1 and AF2 cells presented significantly elevated positive regulatory activity of extracellular matrix assembly. (A) The expression levels of genes involved in " GO: 1901203 positive regulation of extracellular matrix assembly" are shown across 23 subsets. The positive regulatory activity score of the extracellular matrix assembly is shown for each subset, which was calculated via the AddModuleScore function (B) and the AUCell function (C). The positive regulatory activity score of extracellular matrix assembly is shown across 23 subsets in HDs (n=10) and IPF patients (n=8) and was calculated via the AddModuleScore function (D) and the AUCell function (E). (B, C) *, P < 0.05 for comparisons of AF1 cells with all other subgroups except AF2 cells; #, P < 0.05 for comparisons of AF2 cells with all other

subgroups except AF1 cells. P values were determined using (B and C) 1-way ANOVA with Tukey's post-hoc test or (D and E) 2-way ANOVA (*$P < 0.05$) with Tukey's post-hoc test. The data are presented as (B, C, D and E) the means ± SDs.

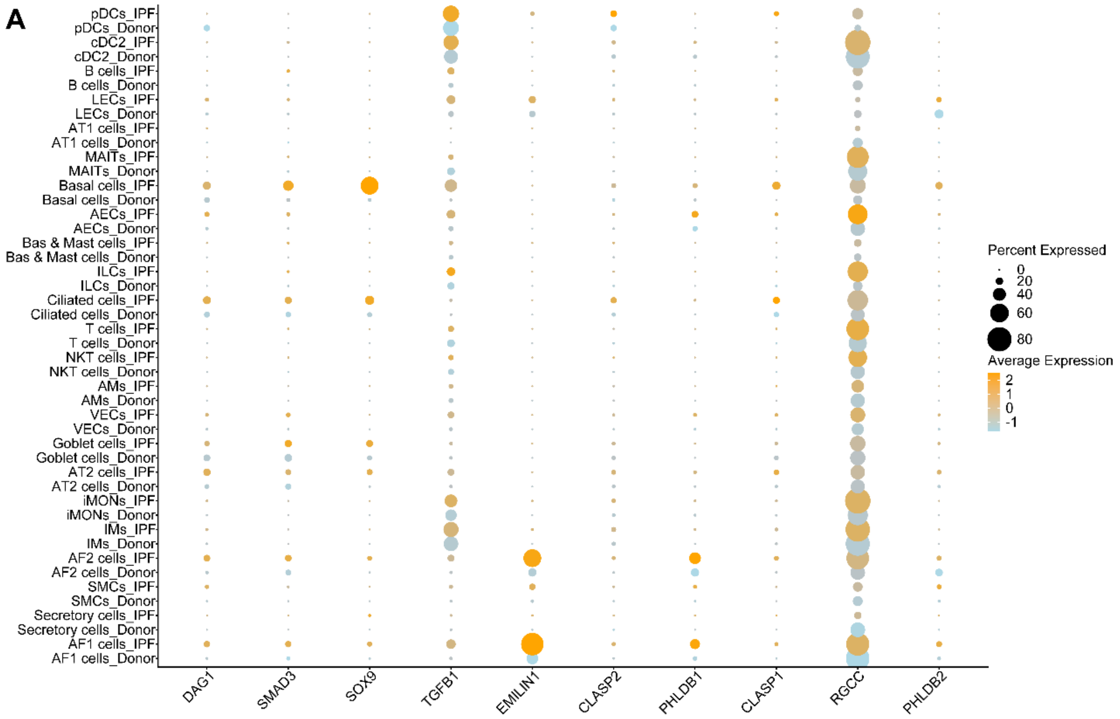

Figure S24. The expression levels of genes involved in the positive regulation of extracellular matrix assembly are shown across 23 subsets in both HDs (n=10) and IPF patients (n=8).

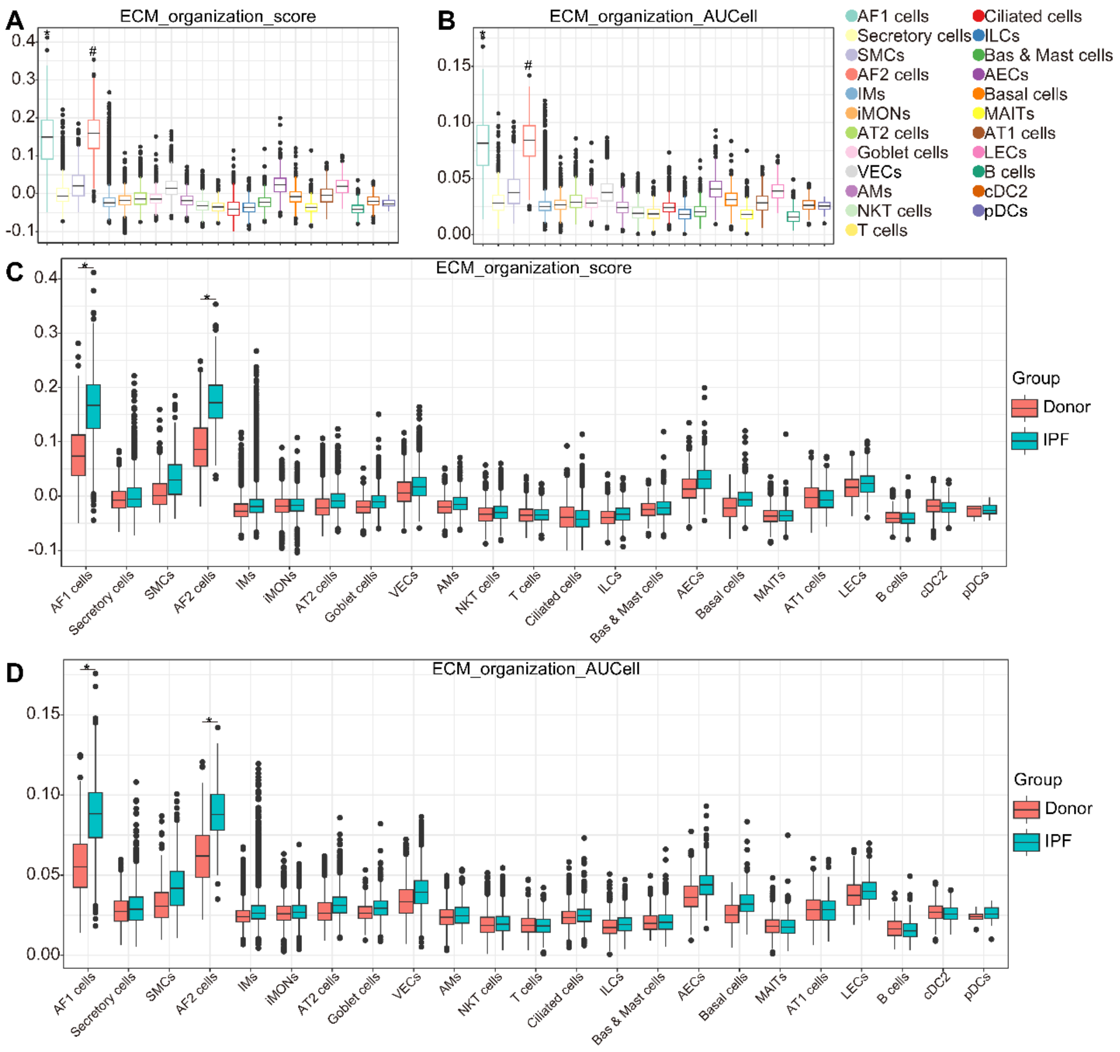

Figure S25. AF1 and AF2 cells presented significantly elevated extracellular matrix organization activity. The activity score of "GO: 0030198 extracellular matrix organization" is shown in each subset calculated by the AddModuleScore function (A) and the AUCell function (B). The extracellular matrix organization score is shown across 23 subsets in HDs (n=10) and IPF patients (n=8) and was calculated via the AddModuleScore function (C) and the AUCell function (D). (A, B) *, $P < 0.05$ for comparisons of AF1 cells with all other subgroups except AF2 cells; #, $P < 0.05$ for comparisons of AF2 cells with all other subgroups except AF1 cells. P values were determined using (A and B) 1-way ANOVA with Tukey's post-hoc test or (C and D) 2-way ANOVA (*$P < 0.05$) with Tukey's post-hoc test. The data are presented as (A, B, C and D) the means ± SDs.

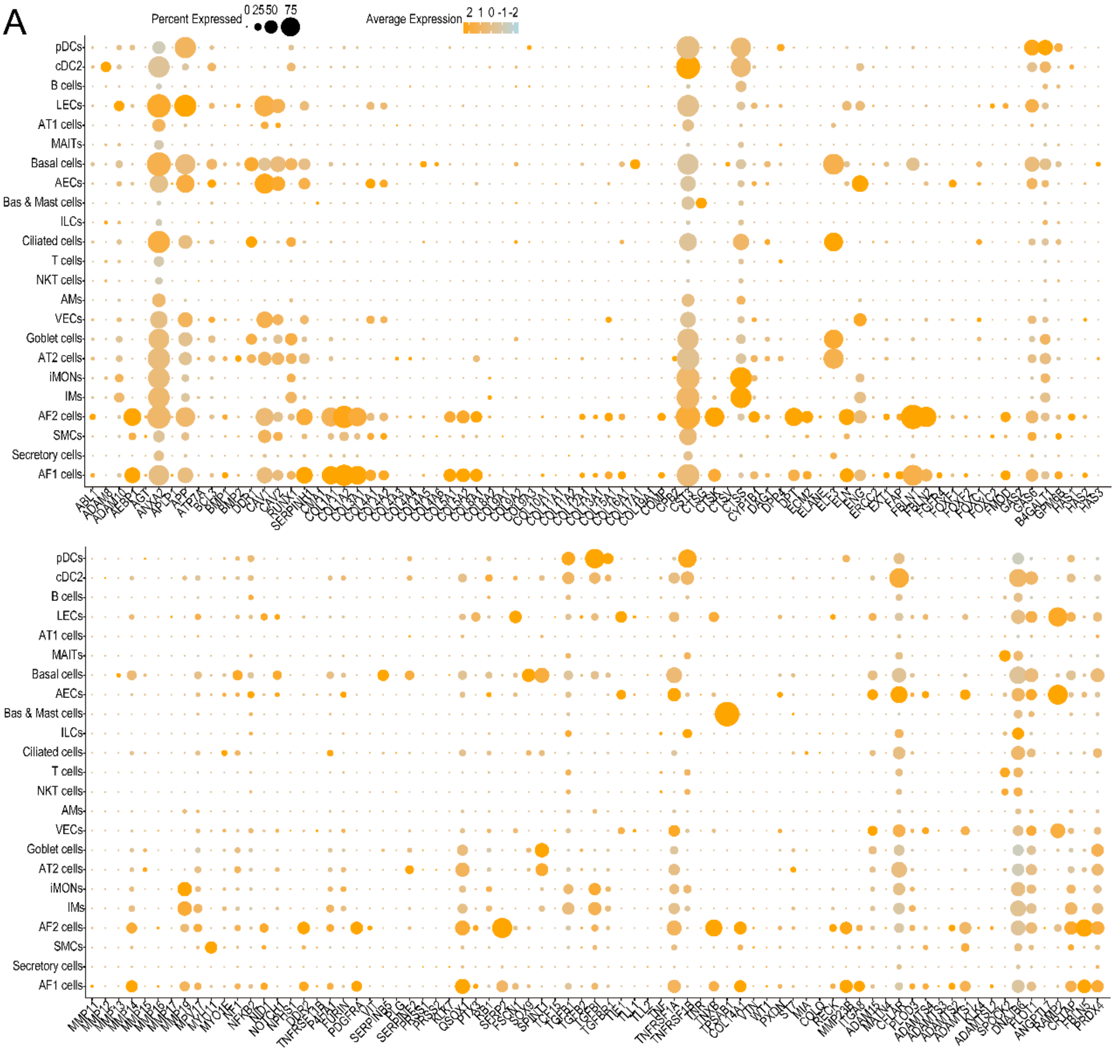

Figure S26. The expression levels of genes involved in extracellular matrix organization are shown in 23 subsets.

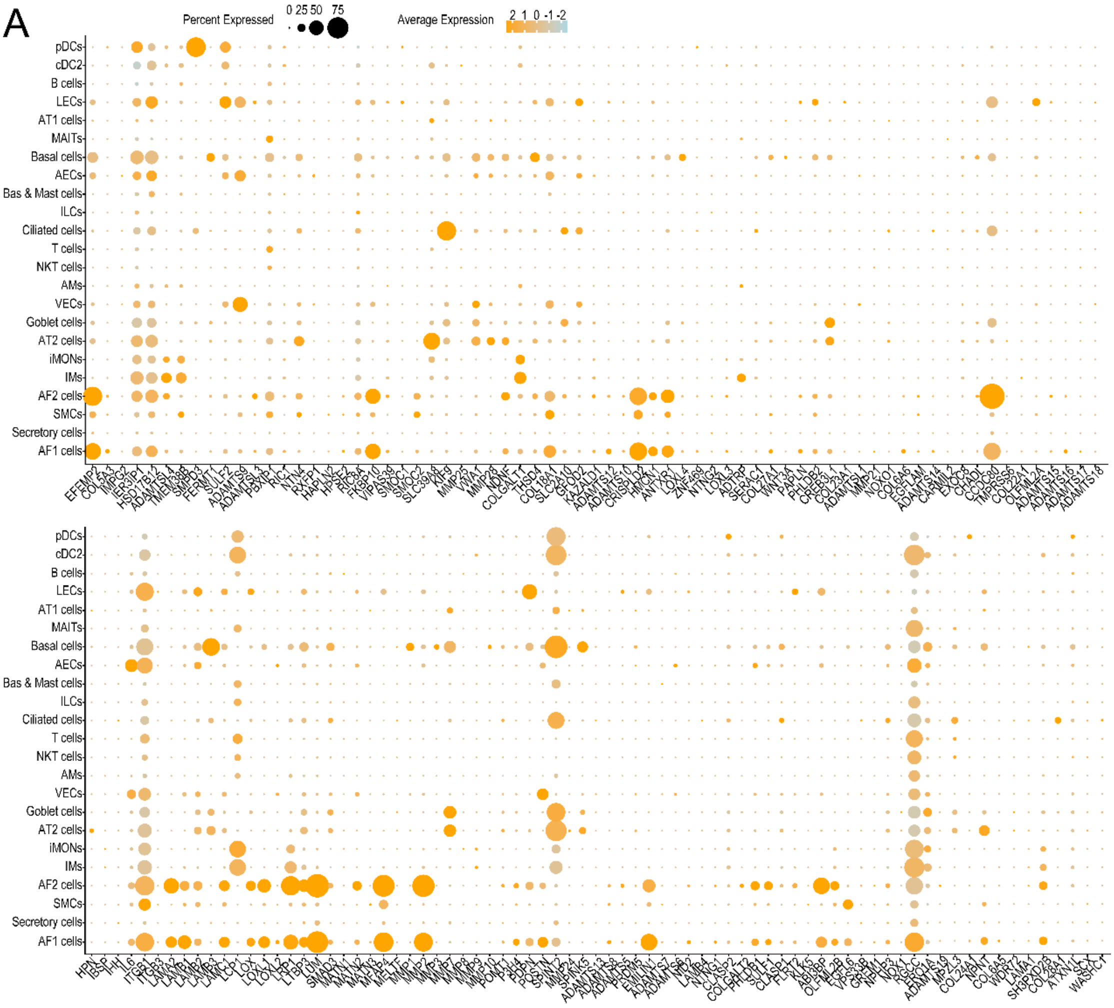

Figure S27. The expression levels of several genes involved in extracellular matrix organization are shown in 23 subsets.

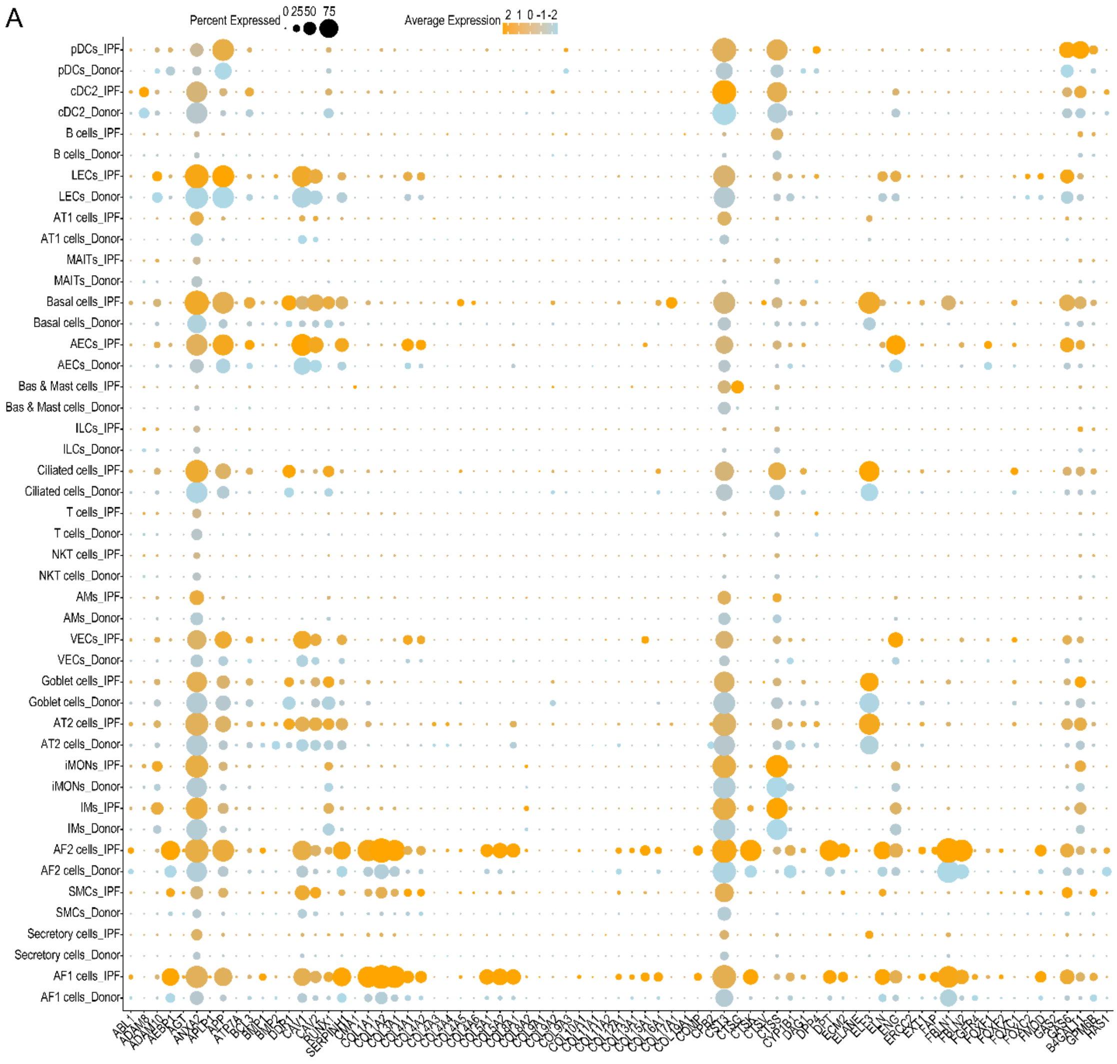

Figure S28. The expression levels of several genes involved in extracellular matrix organization are shown across 23 subsets in both HDs (n=10) and IPF patients (n=8).

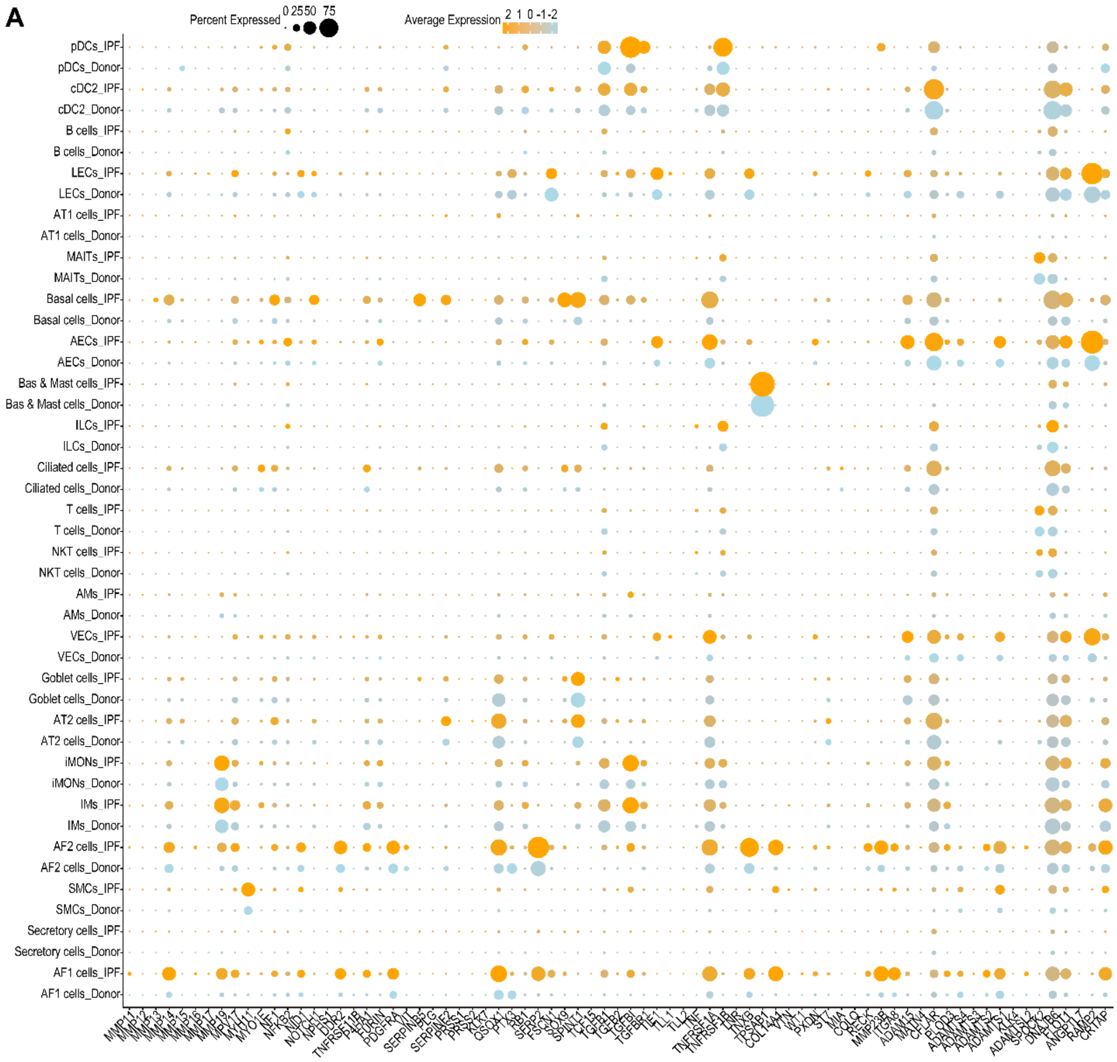

Figure S29. The expression levels of several genes involved in extracellular matrix organization are shown across 23 subsets in both HDs (n=10) and IPF patients (n=8).

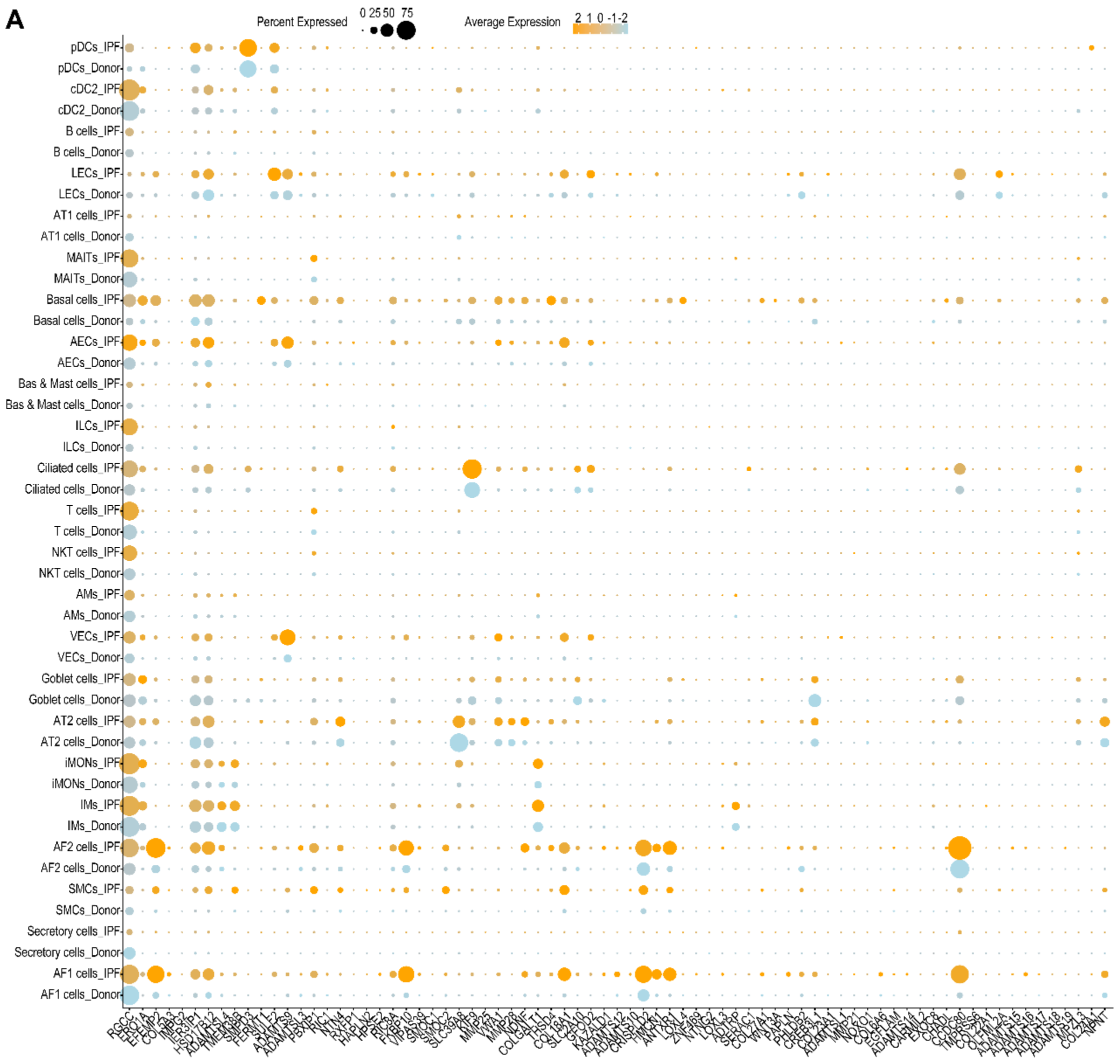

Figure S30. The expression levels of several genes involved in extracellular matrix organization are shown across 23 subsets in both HDs (n=10) and IPF patients (n=8).

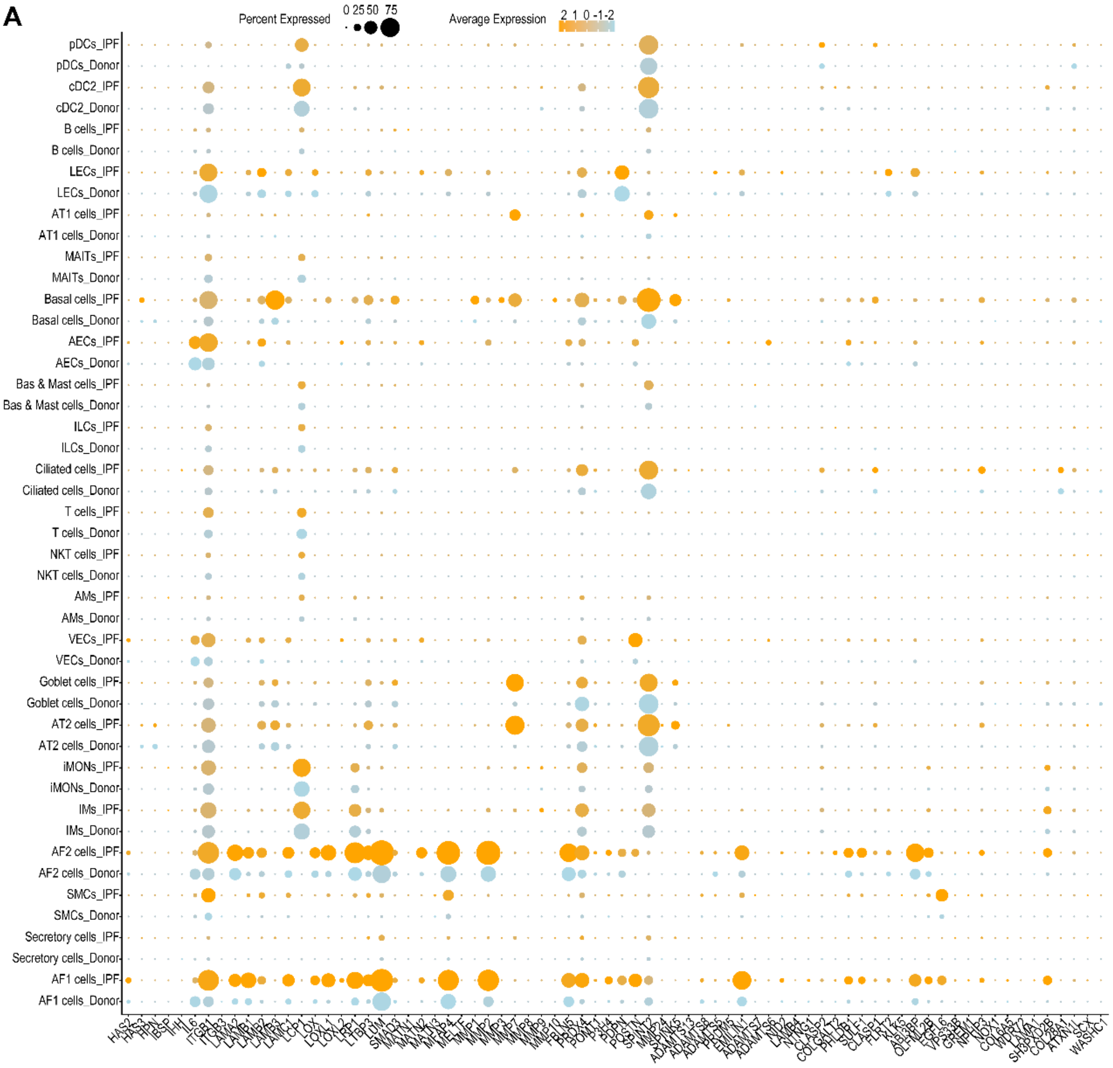

Figure S31. The expression levels of several genes involved in extracellular matrix organization are shown across 23 subsets in both HDs (n=10) and IPF patients (n=8).

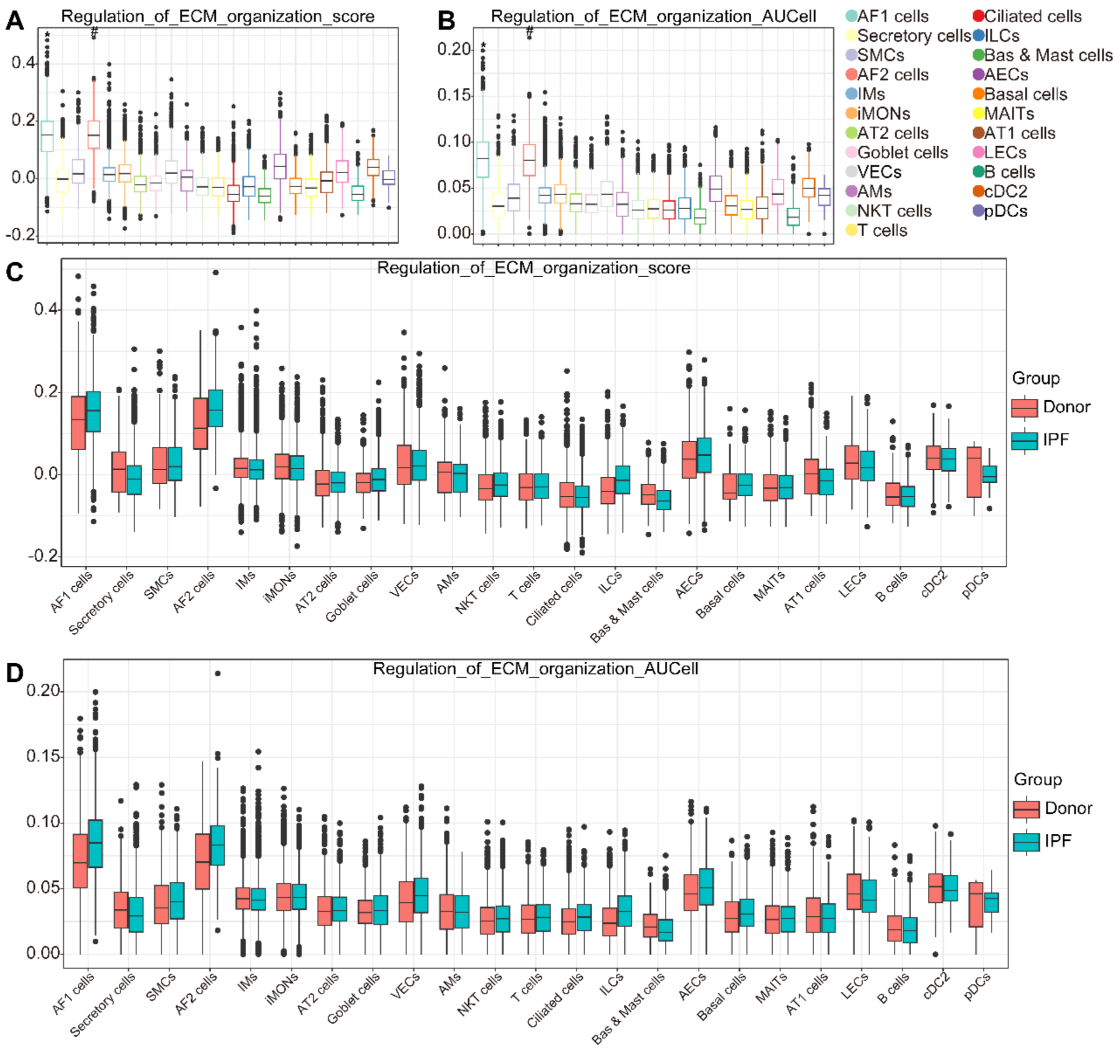

Figure S32. AF1 and AF2 cells exhibited significantly elevated regulatory activity of extracellular matrix organization. The regulatory activity score of "GO: 1903053 regulation of extracellular matrix organization" is shown for each subset and was calculated via the AddModuleScore function (A) and the AUCell function (B). The regulatory activity score of extracellular matrix organization is shown across 23 subsets in HDs (n=10) and IPF patients (n=8) and was calculated via the AddModuleScore function (C) and the AUCell function (D). (A, B) *, $P < 0.05$ for comparisons of AF1 cells with all other subgroups except AF2 cells; #, $P < 0.05$ for comparisons of AF2 cells with all other subgroups except AF1 cells. P values were determined using (A and B) 1-way ANOVA with Tukey's post-hoc test or (C and D) 2-way ANOVA (*$P < 0.05$) with Tukey's post-hoc test. The data are presented as (A, B, C and D) the means ± SDs.

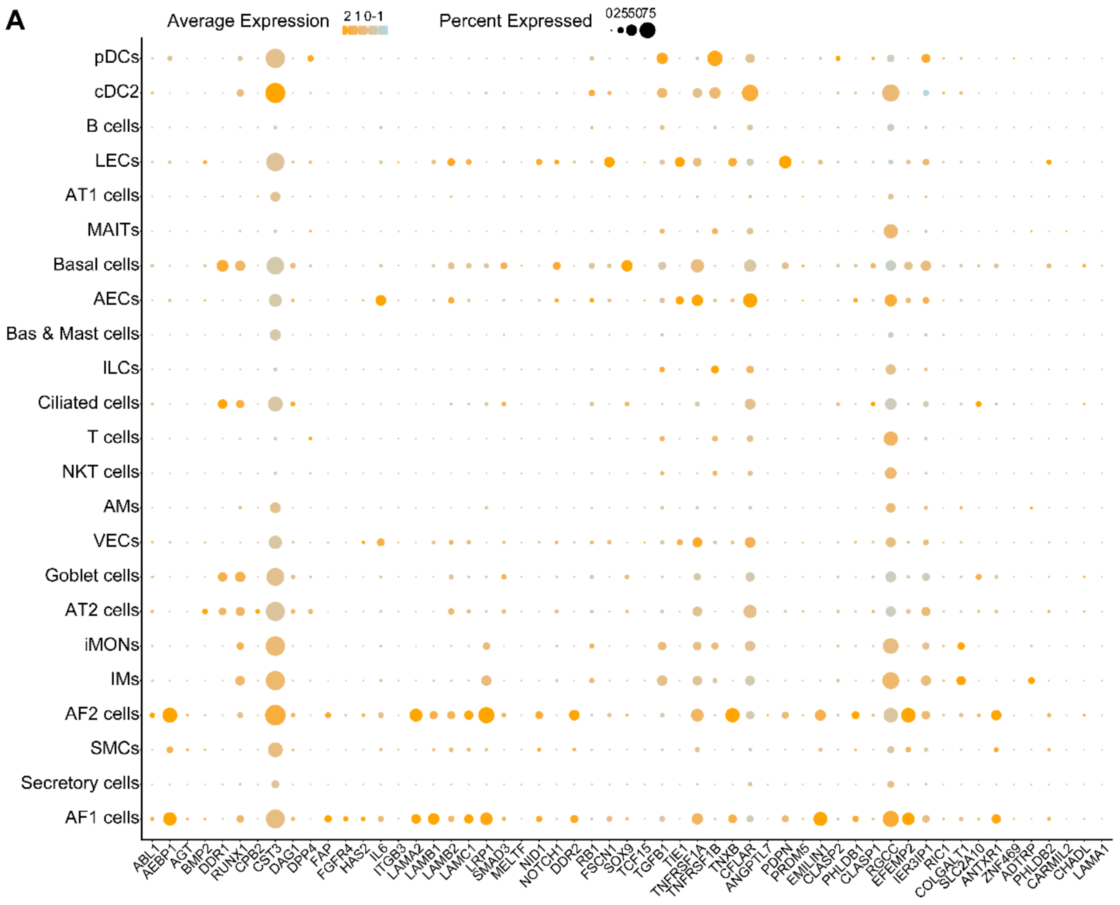

Figure S33. The expression levels of genes involved in the regulation of extracellular matrix organization are shown in 23 subsets.

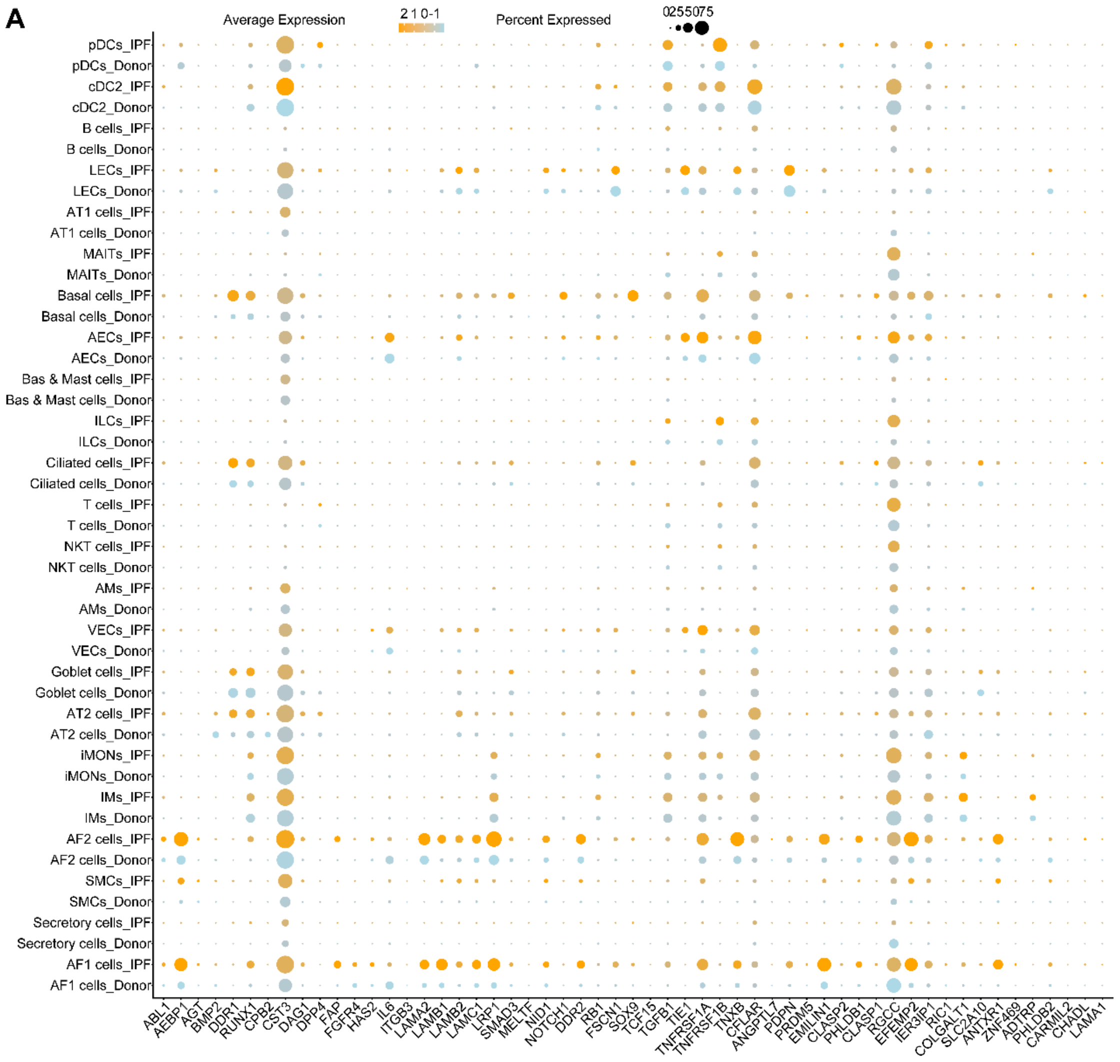

Figure S34. The expression levels of genes involved in the regulation of extracellular matrix organization are shown across 23 subsets in both HDs (n=10) and IPF patients (n=8).

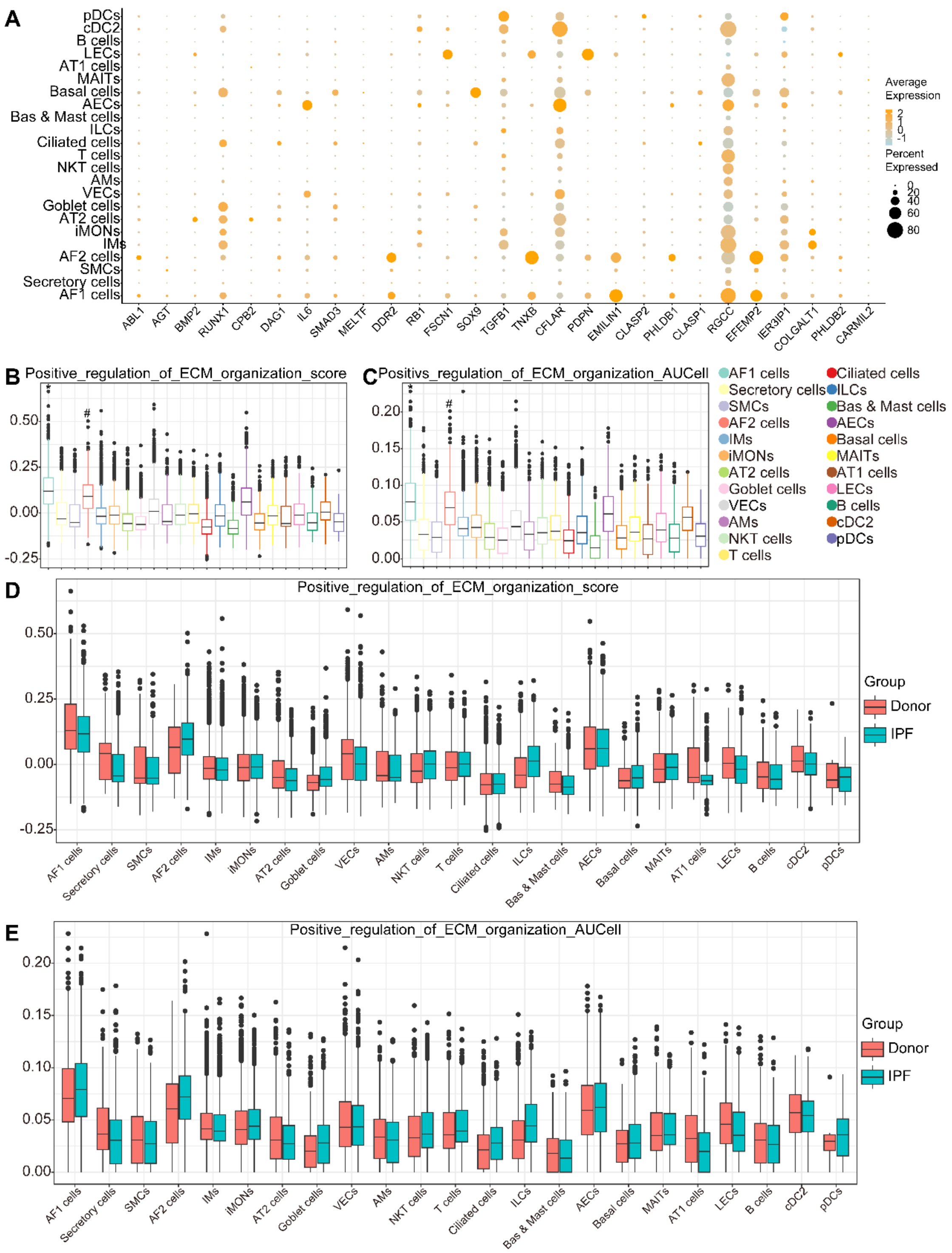

Figure S35. AF1 and AF2 cells presented significantly elevated positive regulatory activity of extracellular matrix organization. (A) The expression levels of genes involved in "GO: 1901203 positive regulation of extracellular matrix organization" are shown across 23 subsets. The positive regulatory activity score of extracellular matrix organization is shown for each subset and was calculated via the AddModuleScore function (B) and the AUCell function (C). The positive regulatory activity score of extracellular matrix organization is shown across 23 subsets in HDs (n=10) and IPF patients (n=8) calculated by the AddModuleScore function (D) and the AUCell function

(E). (B, C) *, $P < 0.05$ for comparisons of AF1 cells with all other subgroups except AF2 cells; #, $P < 0.05$ for comparisons of AF2 cells with all other subgroups except AF1 cells. P values were determined using (B and C) 1-way ANOVA with Tukey's post-hoc test or (D and E) 2-way ANOVA (*$P < 0.05$) with Tukey's post-hoc test. The data are presented as (B, C, D and E) the means ± SDs.

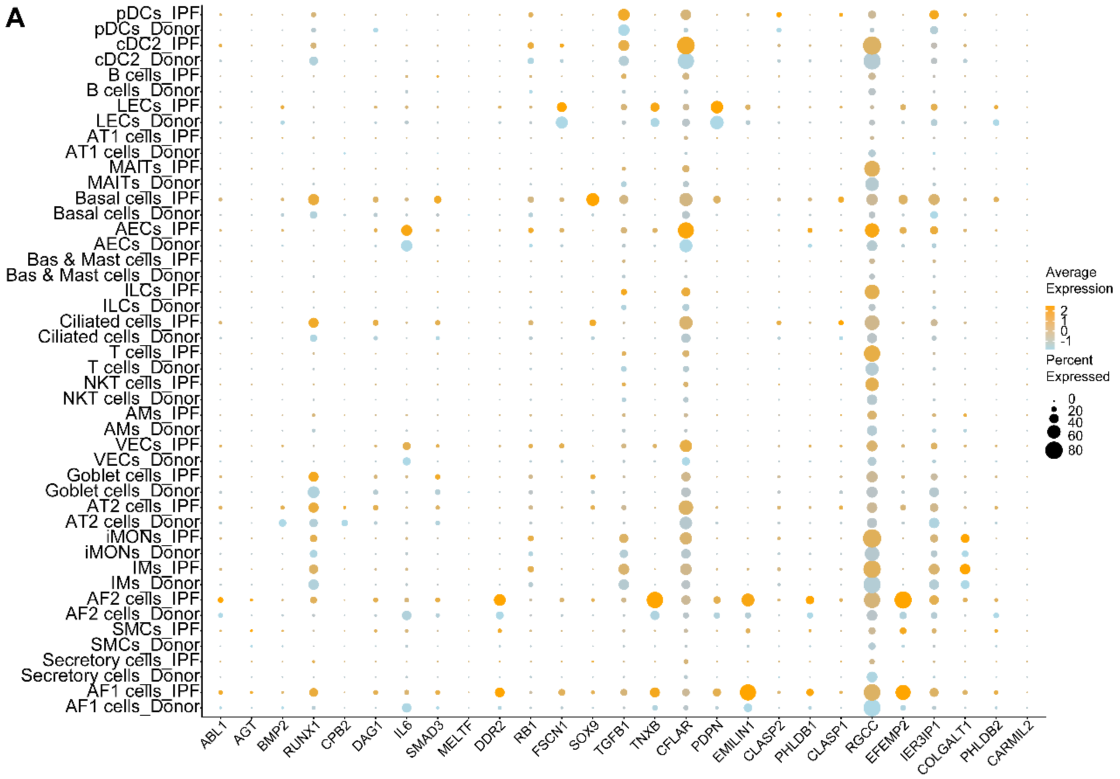

Figure S36. The expression levels of genes involved in the positive regulation of extracellular matrix organization are shown across 23 subsets in both HDs (n=10) and IPF patients (n=8).

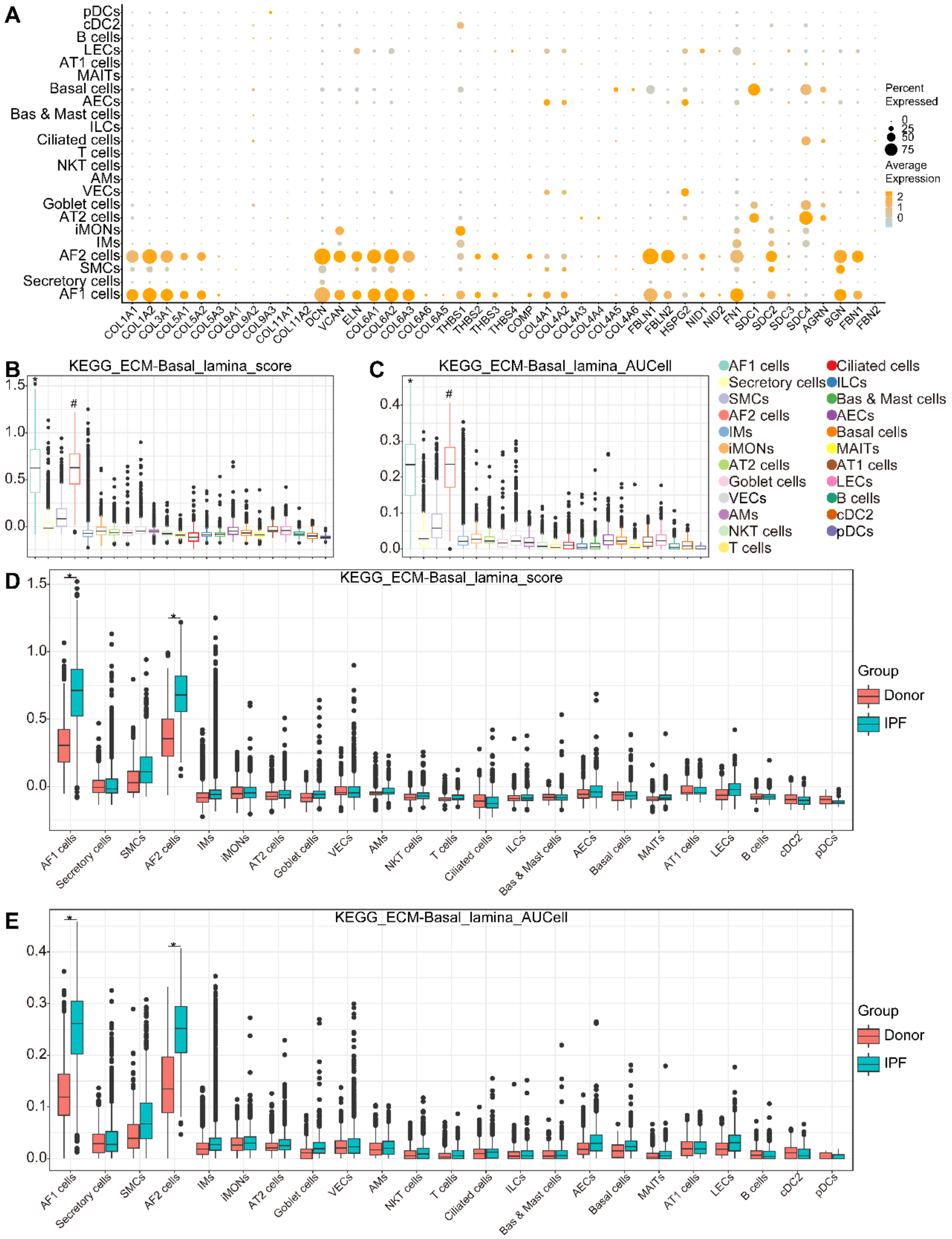

Figure S37. AF1 and AF2 cells presented significantly elevated extracellular matrix - basal lamina activity. (A) The expression levels of genes involved in "KEGG: N01814 extracellular matrix - basal lamina" are shown across 23 subsets. The activity score of the extracellular matrix is shown for each subset calculated via the AddModuleScore function (B) and the AUCell function (C). The extracellular matrix score of the 23 subsets is shown in HDs (n=10) and IPF patients (n=8) and was calculated via the AddModuleScore function (D) and the AUCell function (E). (B, C) *, P < 0.05 for comparisons of AF1 cells with all other subgroups except AF2 cells; #, P < 0.05 for comparisons of AF2 cells with all other subgroups except AF1 cells. P values were

determined using (B and C) 1-way ANOVA with Tukey's post-hoc test or (D and E) 2-way ANOVA (*$P < 0.05$) with Tukey's post-hoc test. The data are presented as (B, C, D and E) the means ± SDs.

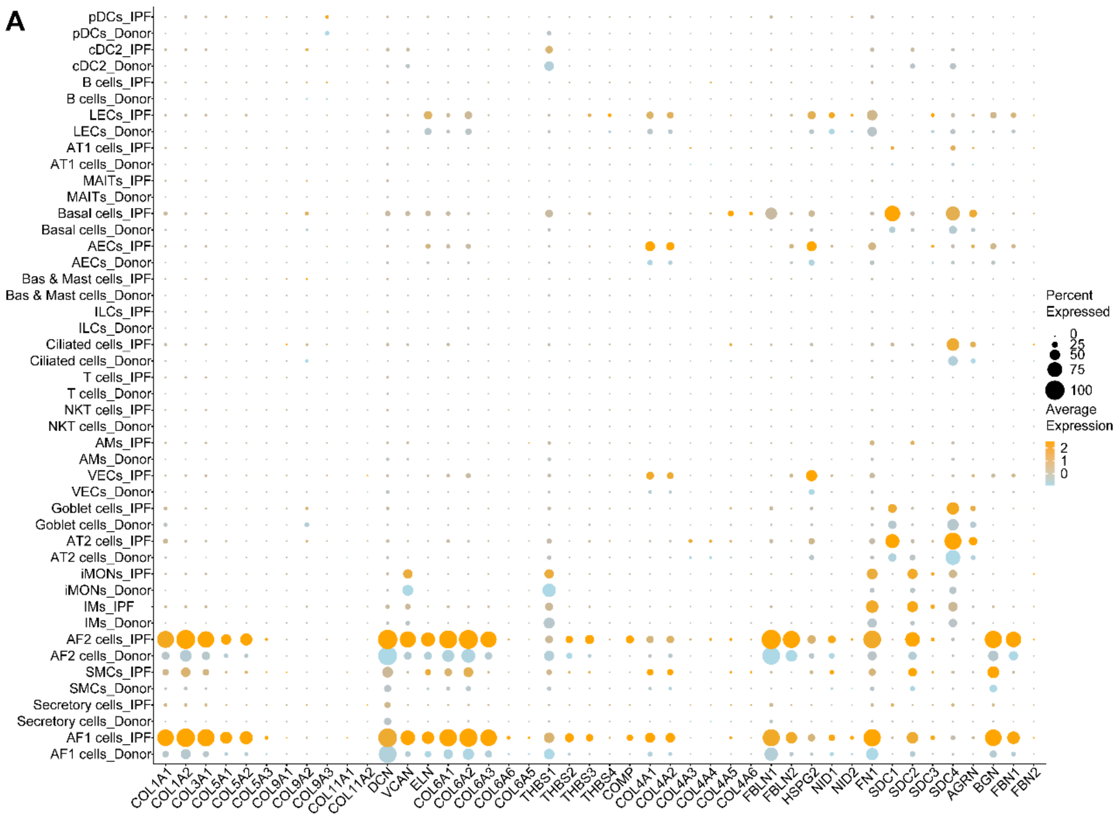

Figure S38. The expression levels of genes involved in the extracellular matrix - basal lamina are shown across 23 subsets in both HDs (n=10) and IPF patients (n=8).

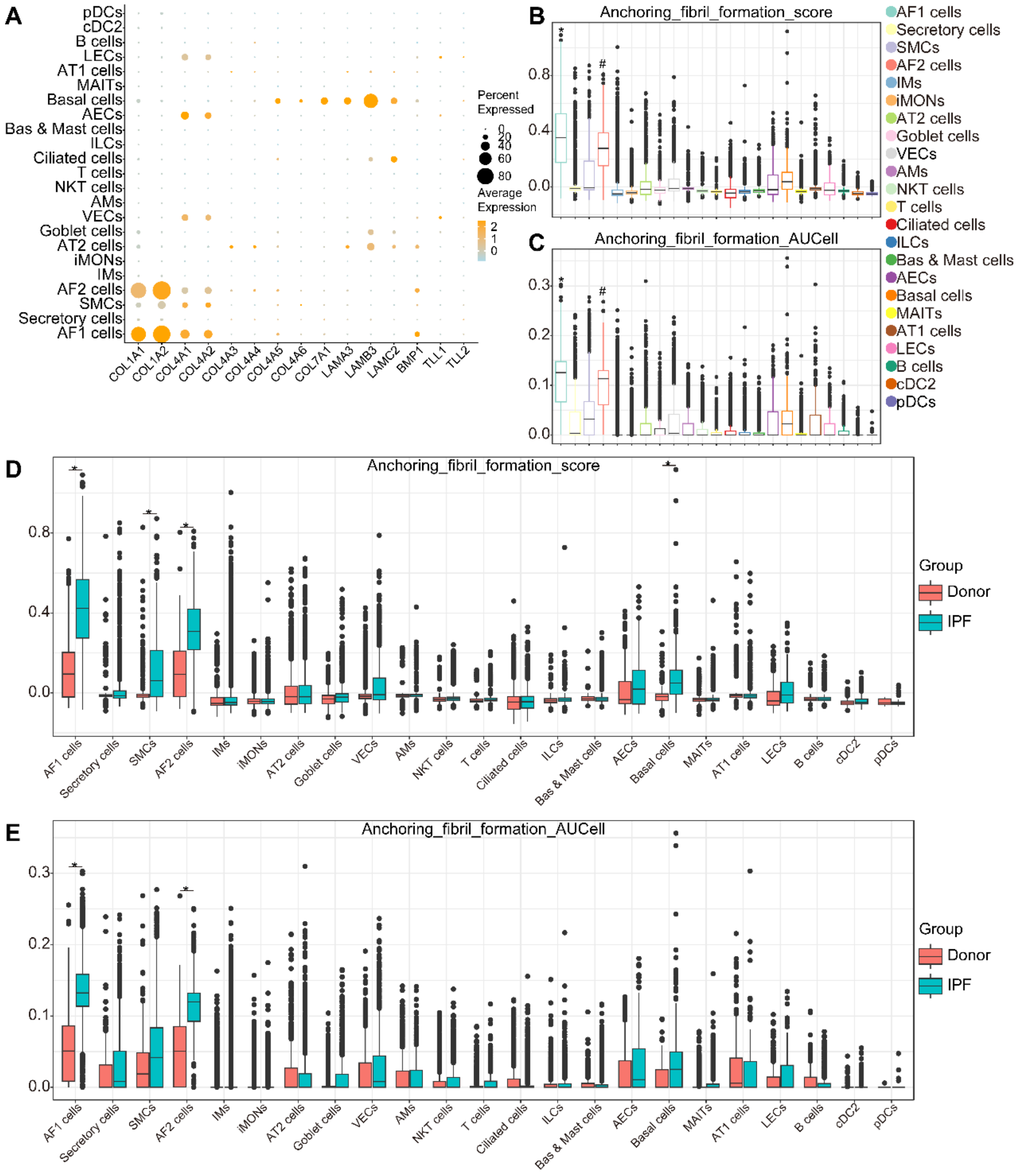

Figure S39. AF1 and AF2 cells presented significantly increased anchoring fibril formation activity. (A) The expression levels of genes involved in "Reactome: R-HSA-2214320 anchoring fibril formation" are shown across 23 subsets. The activity score of anchoring fibril formation is shown for each subset and was calculated via the AddModuleScore function (B) and the AUCell function (C). The anchoring fibril formation score of the 23 subsets is shown for HDs (n=10) and IPF patients (n=8) and was calculated via the AddModuleScore function (D) and the AUCell function (E). (B, C) *, P < 0.05 for comparisons of AF1 cells with all other subgroups except AF2 cells; #, P < 0.05 for comparisons of AF2 cells with all other subgroups except AF1 cells. P values were determined using (B and C) 1-way ANOVA with Tukey's post-hoc test or (D and E) 2-way ANOVA (*P < 0.05) with Tukey's post-hoc test. The data are presented as (B, C, D and E) the means ± SDs.

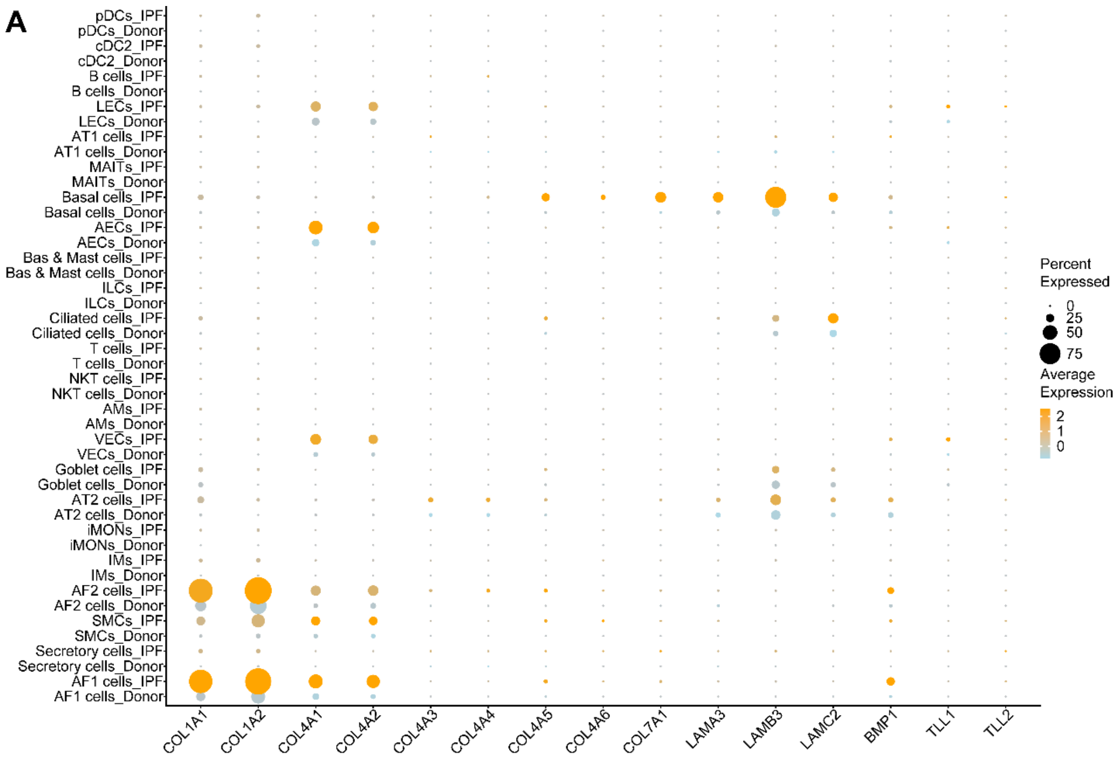

Figure S40. The expression levels of genes involved in anchoring fibril formation are shown across 23 subsets in both HDs (n=10) and IPF patients (n=8).

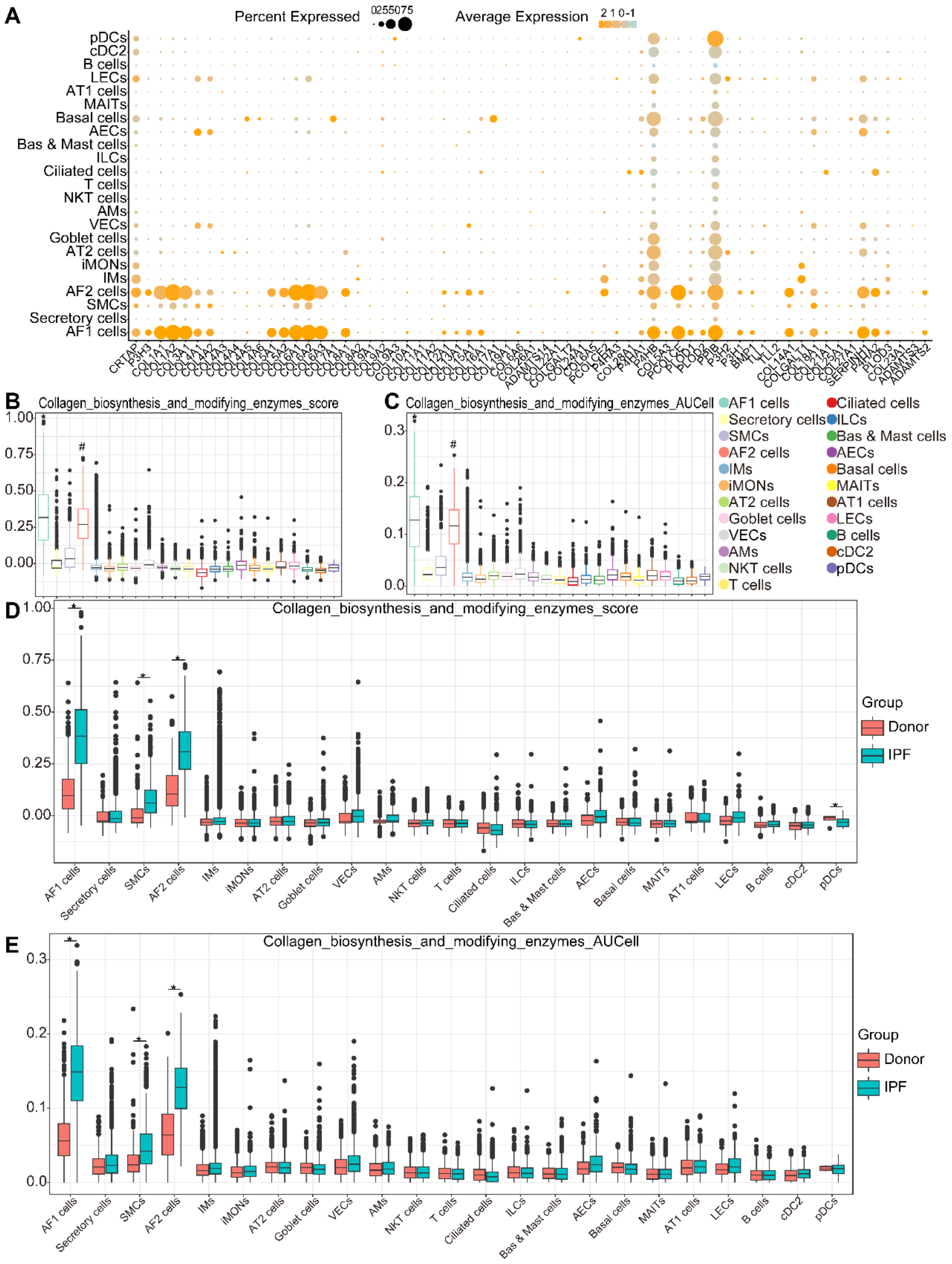

Figure S41. AF1 and AF2 cells presented significantly elevated collagen biosynthesis and modifying enzymes activity. (A) The expression levels of genes involved in "Reactome: R-HSA-1650814 collagen biosynthesis and modifying enzymes" are shown across 23 subsets. The activity score of collagen biosynthesis and modifying enzymes is shown in each subset calculated by the AddModuleScore function (B) and the AUCell function (C). The collagen biosynthesis and modifying enzymes score of the 23 subsets is shown for HDs (n=10) and IPF patients (n=8) and was calculated via the AddModuleScore function (D) and the AUCell function (E). (B, C) *, $P < 0.05$ for

comparisons of AF1 cells with all other subgroups except AF2 cells; #, $P < 0.05$ for comparisons of AF2 cells with all other subgroups except AF1 cells. P values were determined using (B and C) 1-way ANOVA with Tukey's post-hoc test or (D and E) 2-way ANOVA (*$P < 0.05$) with Tukey's post-hoc test. The data are presented as (B, C, D and E) the means ± SDs.

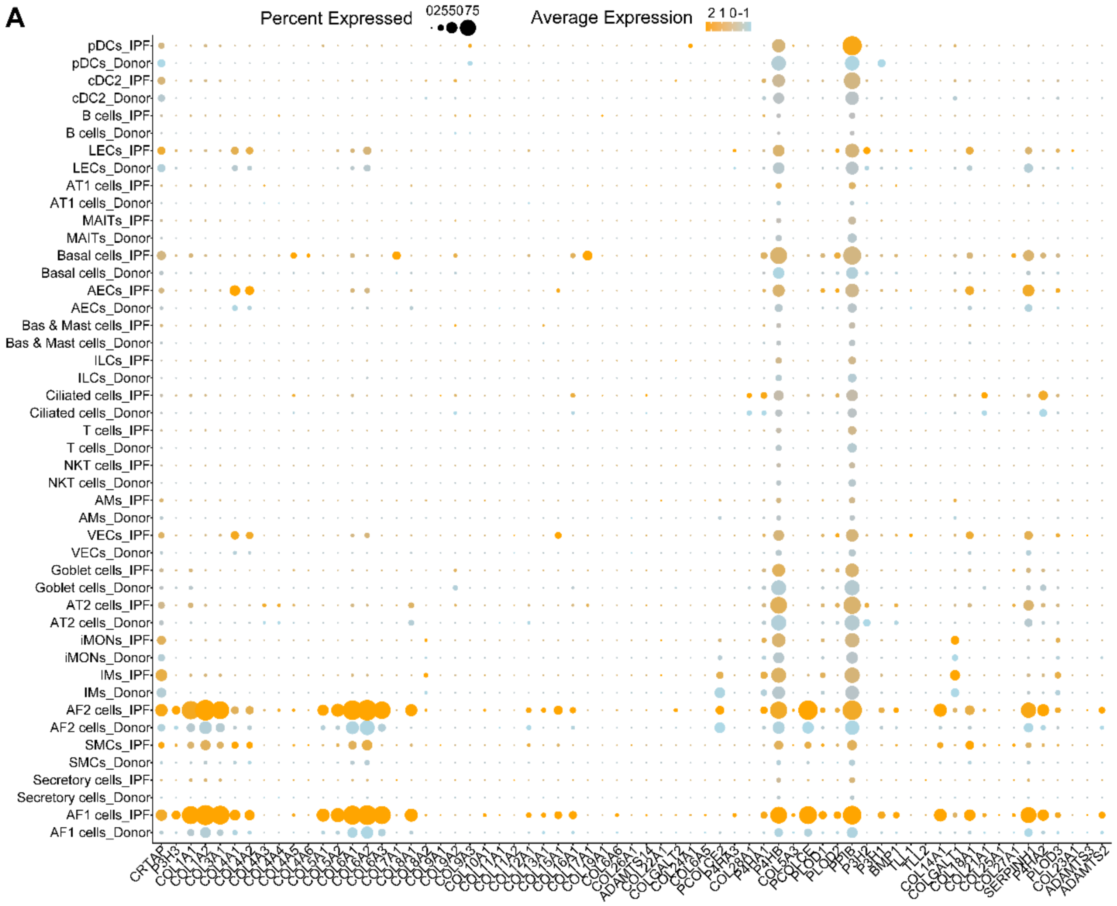

Figure S42. The expression levels of genes involved in collagen biosynthesis and modifying enzymes are shown across 23 subsets in both HDs (n=10) and IPF patients (n=8).

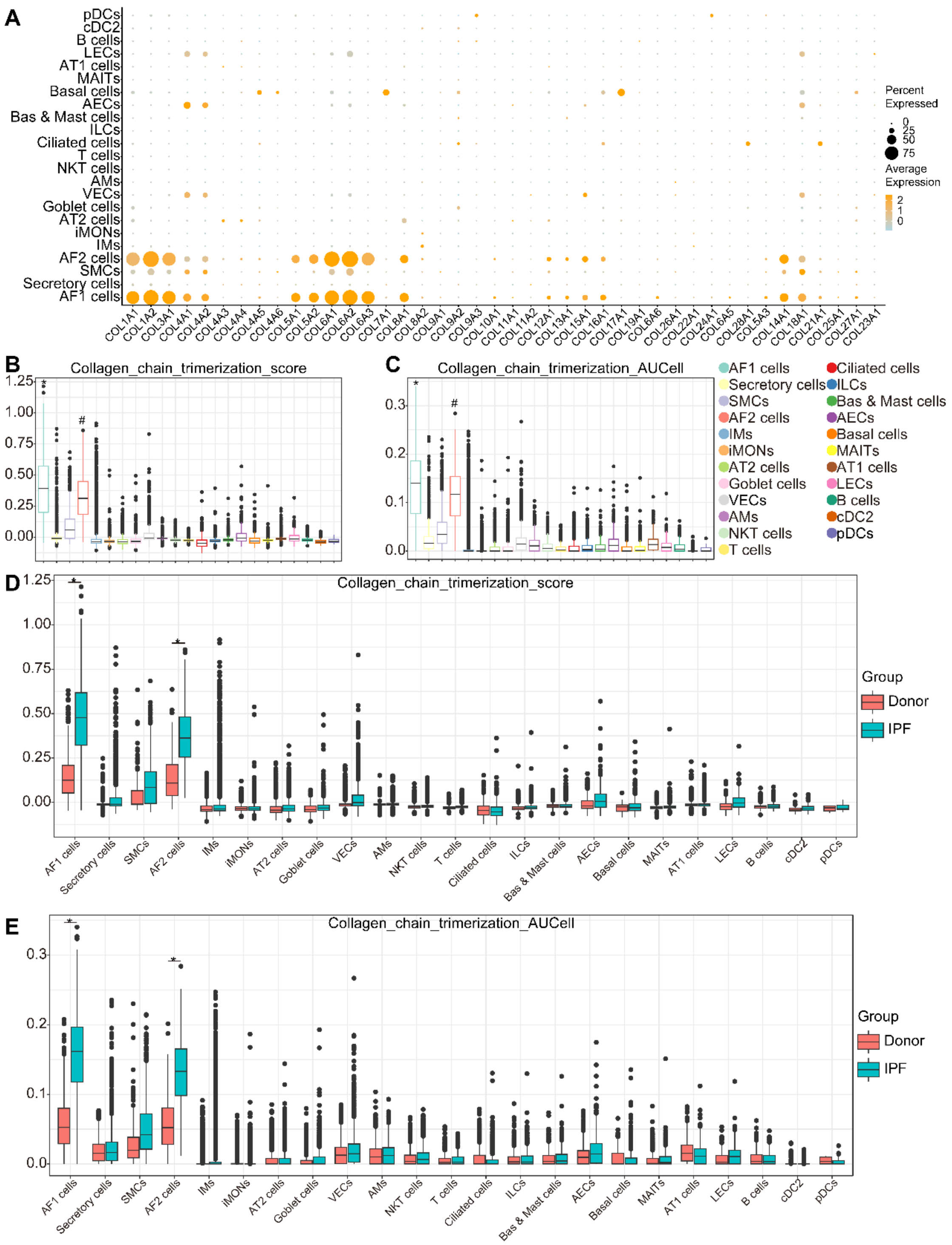

Figure S43. AF1 and AF2 cells exhibited significantly elevated collagen chain trimerization activity. (A) The expression levels of genes involved in "Reactome: R-HSA-8948216 collagen chain trimerization" are shown across 23 subsets. The activity score of collagen chain trimerization is shown for each subset calculated via the AddModuleScore function (B) and the AUCell function (C). The collagen chain trimerization score of the 23 subsets is shown for HDs (n=10) and IPF patients (n=8) and was calculated via the AddModuleScore function (D) and the AUCell function (E). (B, C) *, $P < 0.05$ for comparisons of AF1 cells with all other subgroups except AF2

cells; #, $P < 0.05$ for comparisons of AF2 cells with all other subgroups except AF1 cells. P values were determined using (B and C) 1-way ANOVA with Tukey's post-hoc test or (D and E) 2-way ANOVA (*$P < 0.05$) with Tukey's post-hoc test. The data are presented as (B, C, D and E) the means ± SDs.

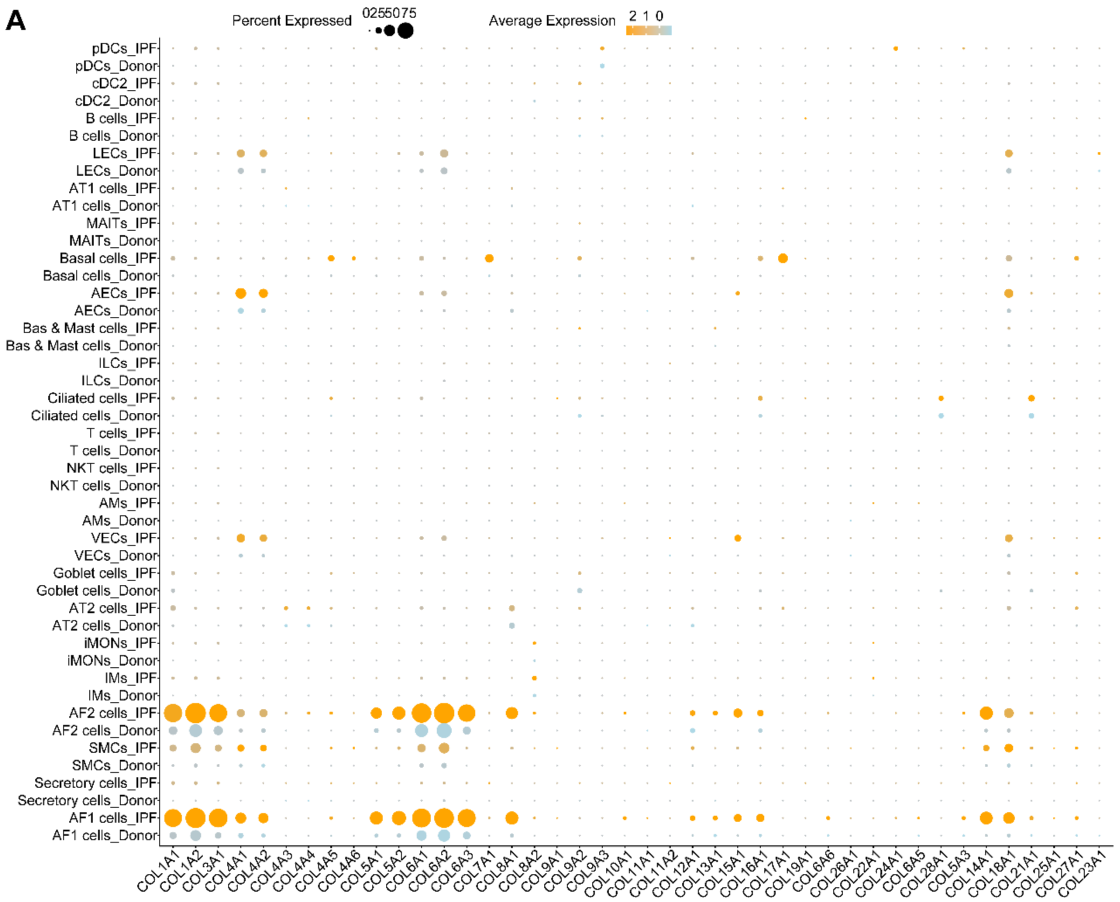

Figure S44. The expression levels of genes involved in collagen chain trimerization are shown across 23 subsets in both HDs (n=10) and IPF patients (n=8).

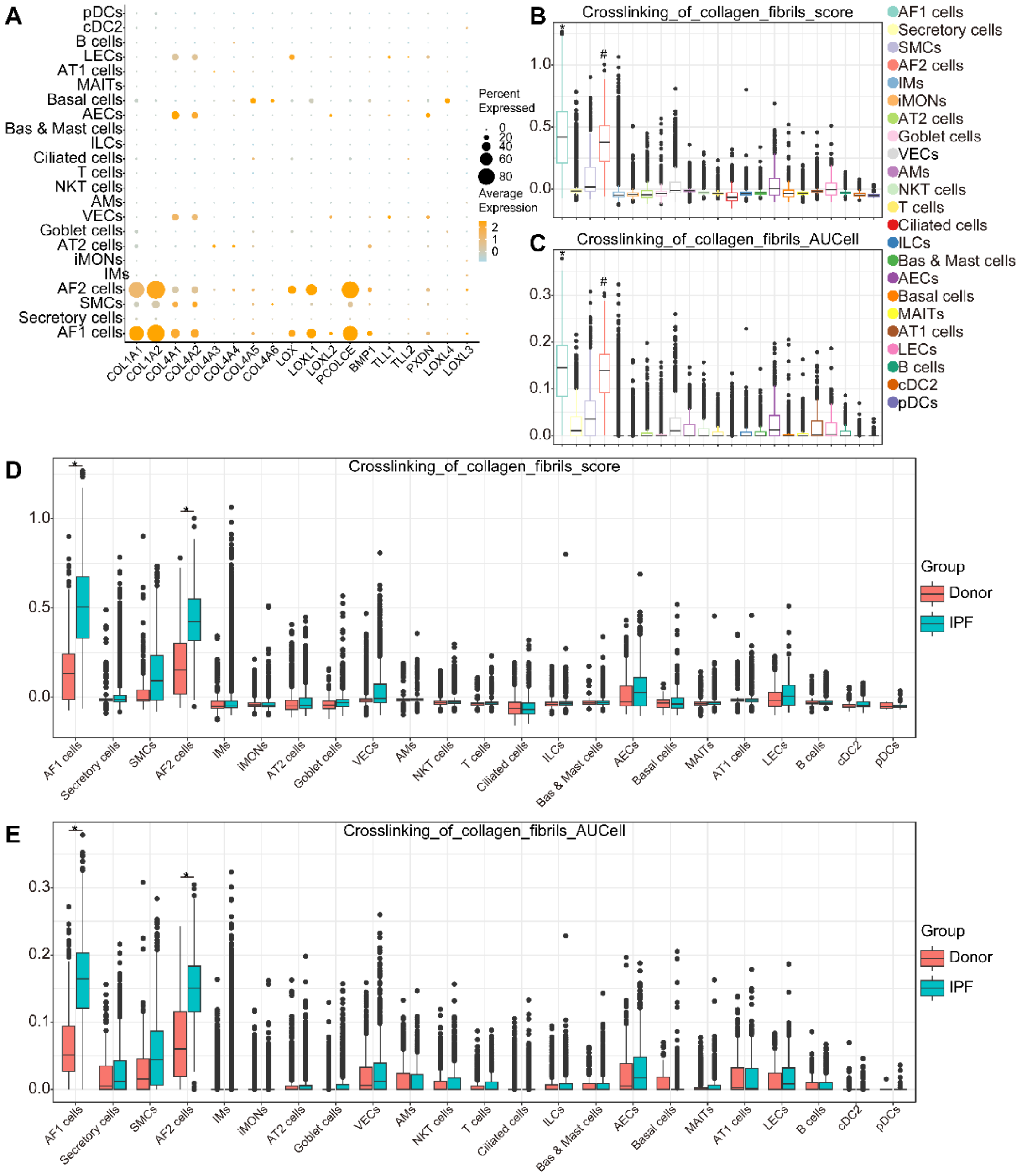

Figure S45. AF1 and AF2 cells exhibited significantly elevated crosslinking activity of collagen fibrils. (A) The expression levels of genes involved in "Reactome: R-HSA-2243919 crosslinking of collagen fibrils" are shown across 23 subsets. The activity score of collagen fibrils crosslinking is shown for each subset and was calculated via the AddModuleScore function (B) and the AUCell function (C). The collagen fibrils crosslinking score of the 23 subsets is shown for HDs (n=10) and IPF patients (n=8) and was calculated via the AddModuleScore function (D) and the AUCell function (E). (B, C) *, P < 0.05 for comparisons of AF1 cells with all other subgroups except AF2 cells; #, P < 0.05 for comparisons of AF2 cells with all other subgroups except AF1 cells. P values were determined using (B and C) 1-way ANOVA with Tukey's post-hoc test or (D and E) 2-way ANOVA (*P < 0.05) with Tukey's post-hoc test. The data are presented as (B, C, D and E) the means ± SDs.

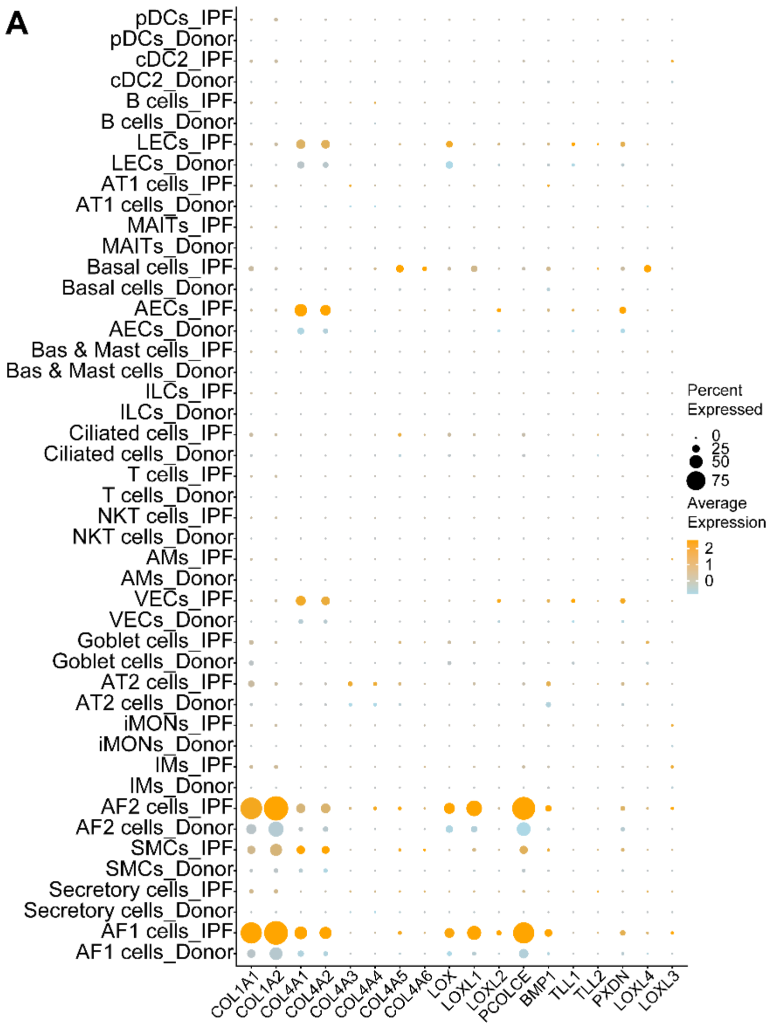

Figure S46. The expression levels of genes involved in the crosslinking of collagen fibrils are shown across 23 subsets in both HDs (n=10) and IPF patients (n=8).

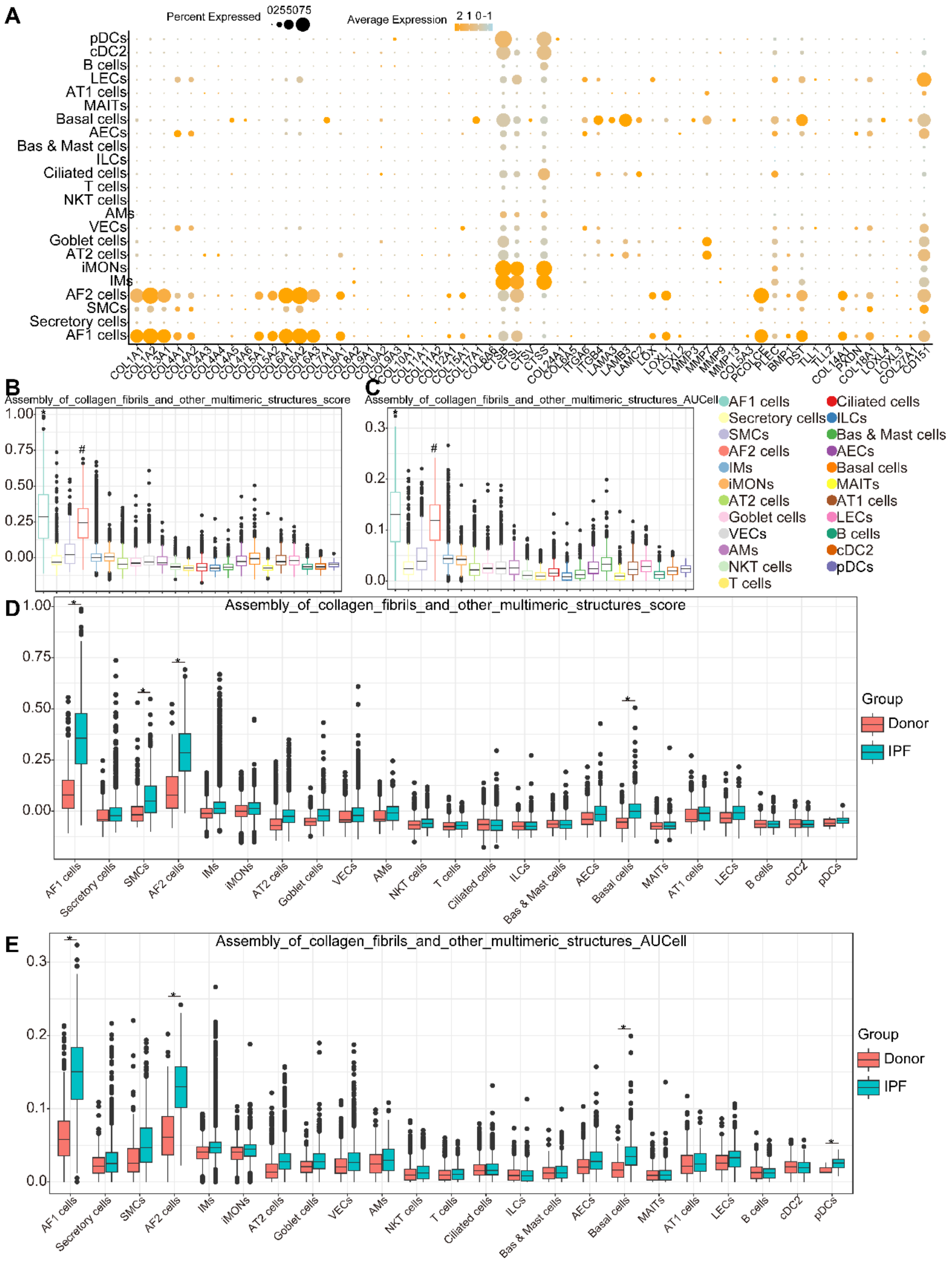

Figure S47. AF1 and AF2 cells exhibited significantly elevated assembly activity of collagen fibrils and other multimeric structures. (A) The expression levels of genes involved in the "Reactome: R-HSA-2022090 assembly of collagen fibrils and other multimeric structures" are shown across 23 subsets. The assembly activity score of the collagen fibrils and other multimeric structures is shown for each subset, which was calculated via the AddModuleScore function (B) and the AUCell function (C). The assembly activity score of collagen fibrils and other multimeric structures is shown across 23 subsets in HDs (n=10) and IPF patients (n=8) and was calculated via the

AddModuleScore function (D) and the AUCell function (E). (B, C) *, $P < 0.05$ for comparisons of AF1 cells with all other subgroups except AF2 cells; #, $P < 0.05$ for comparisons of AF2 cells with all other subgroups except AF1 cells. P values were determined using (B and C) 1-way ANOVA with Tukey's post-hoc test or (D and E) 2-way ANOVA (*$P < 0.05$) with Tukey's post-hoc test. The data are presented as (B, C, D and E) the means ± SDs.

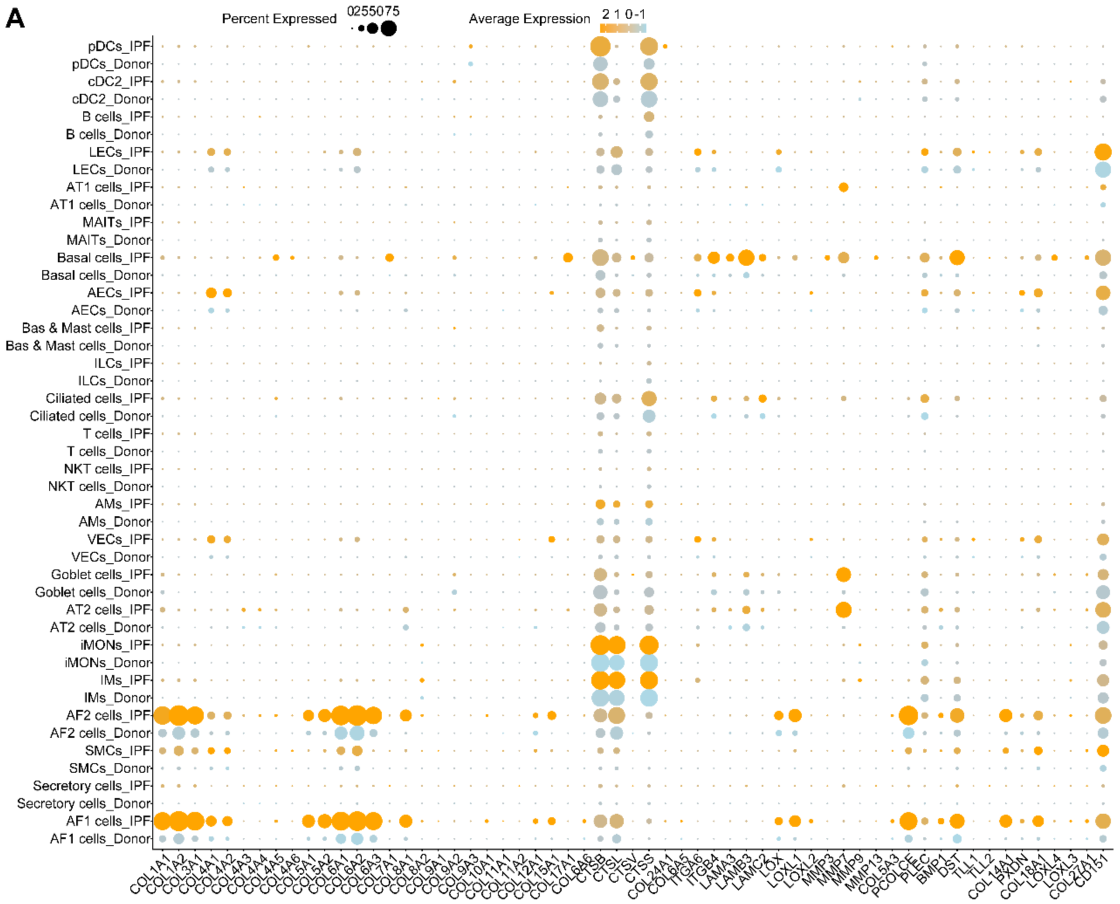

Figure S48. The expression levels of genes involved in the assembly of collagen fibrils and other multimeric structures are shown across 23 subsets in both HDs (n=10) and IPF patients (n=8).

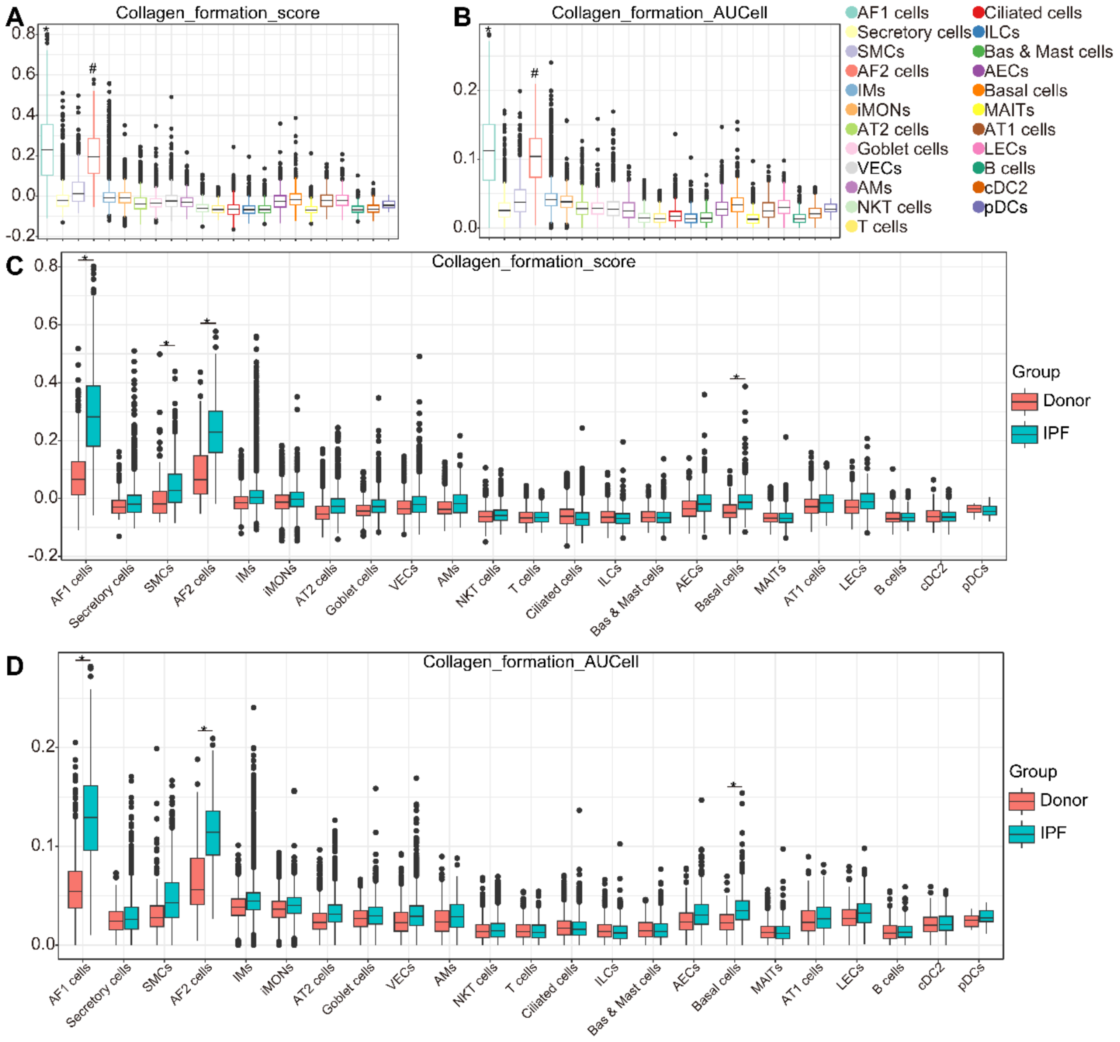

Figure S49. AF1 and AF2 cells exhibited significantly elevated collagen formation activity. The activity score of "Reactome: R-HSA-1474290 collagen formation" is shown for each subset and was calculated via the AddModuleScore function (A) and the AUCell function (B). The collagen formation score is shown across 23 subsets in HDs (n=10) and IPF patients (n=8) and was calculated via the AddModuleScore function (C) and the AUCell function (D). (A, B) *, P < 0.05 for comparisons of AF1 cells with all other subgroups except AF2 cells; #, P < 0.05 for comparisons of AF2 cells with all other subgroups except AF1 cells. P values were determined using (A and B) 1-way ANOVA with Tukey's post-hoc test or (C and D) 2-way ANOVA (*P < 0.05) with Tukey's post-hoc test. The data are presented as (A, B, C and D) the means ± SDs.

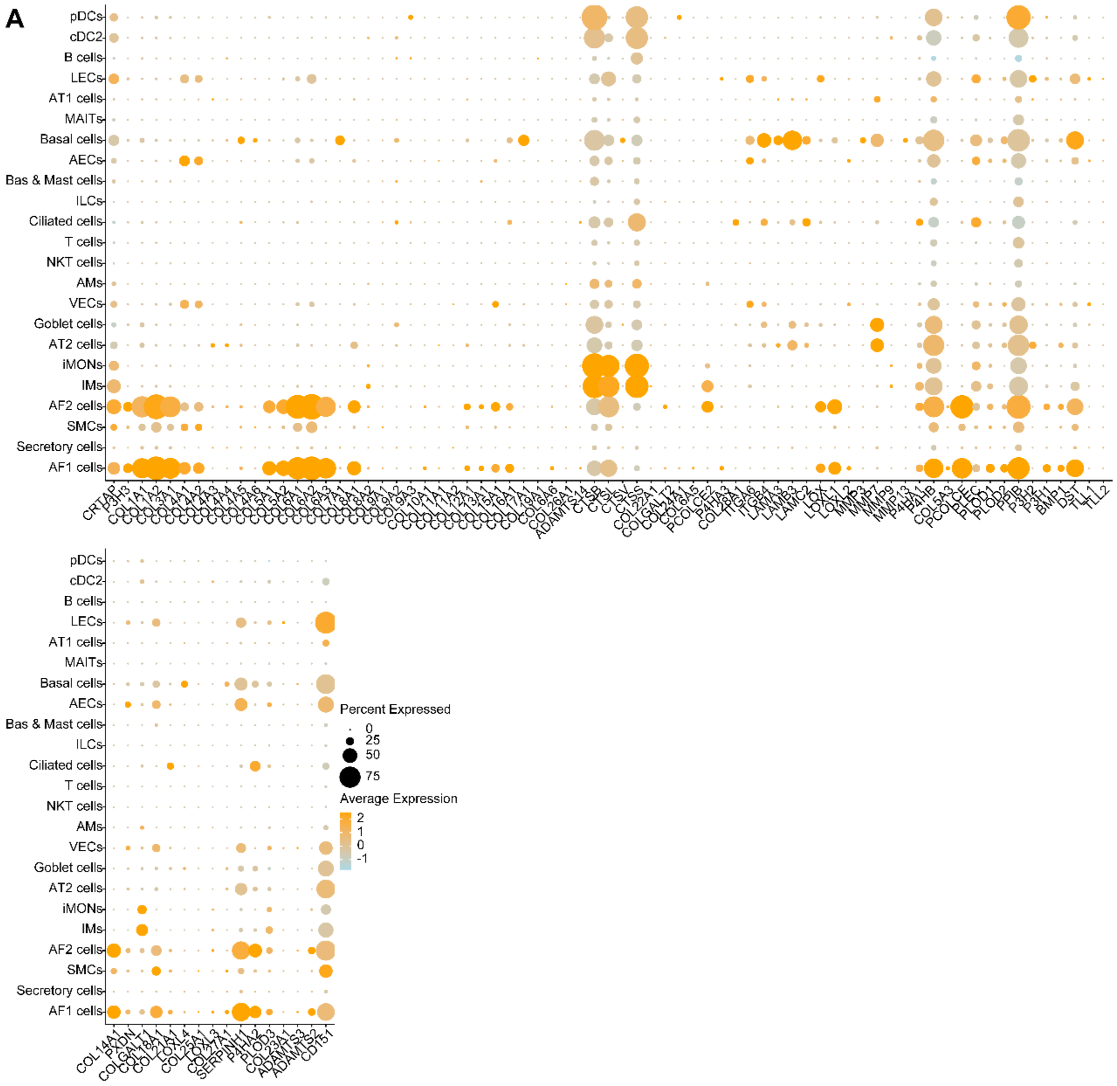

Figure S50. The expression levels of genes involved in collagen formation are shown in 23 subsets.

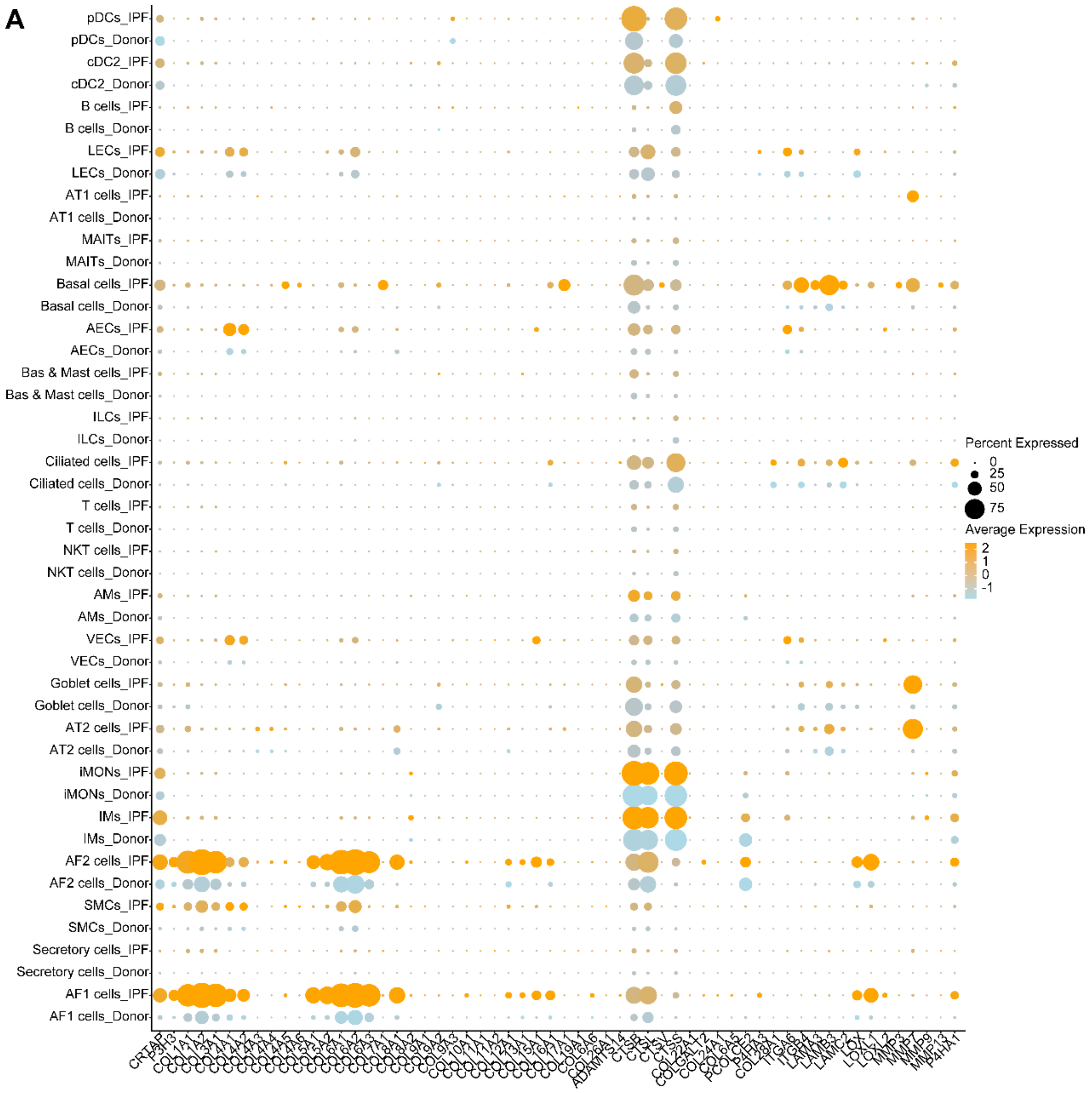

Figure S51. The expression levels of several genes involved in collagen formation are shown across 23 subsets in both HDs (n=10) and IPF patients (n=8).

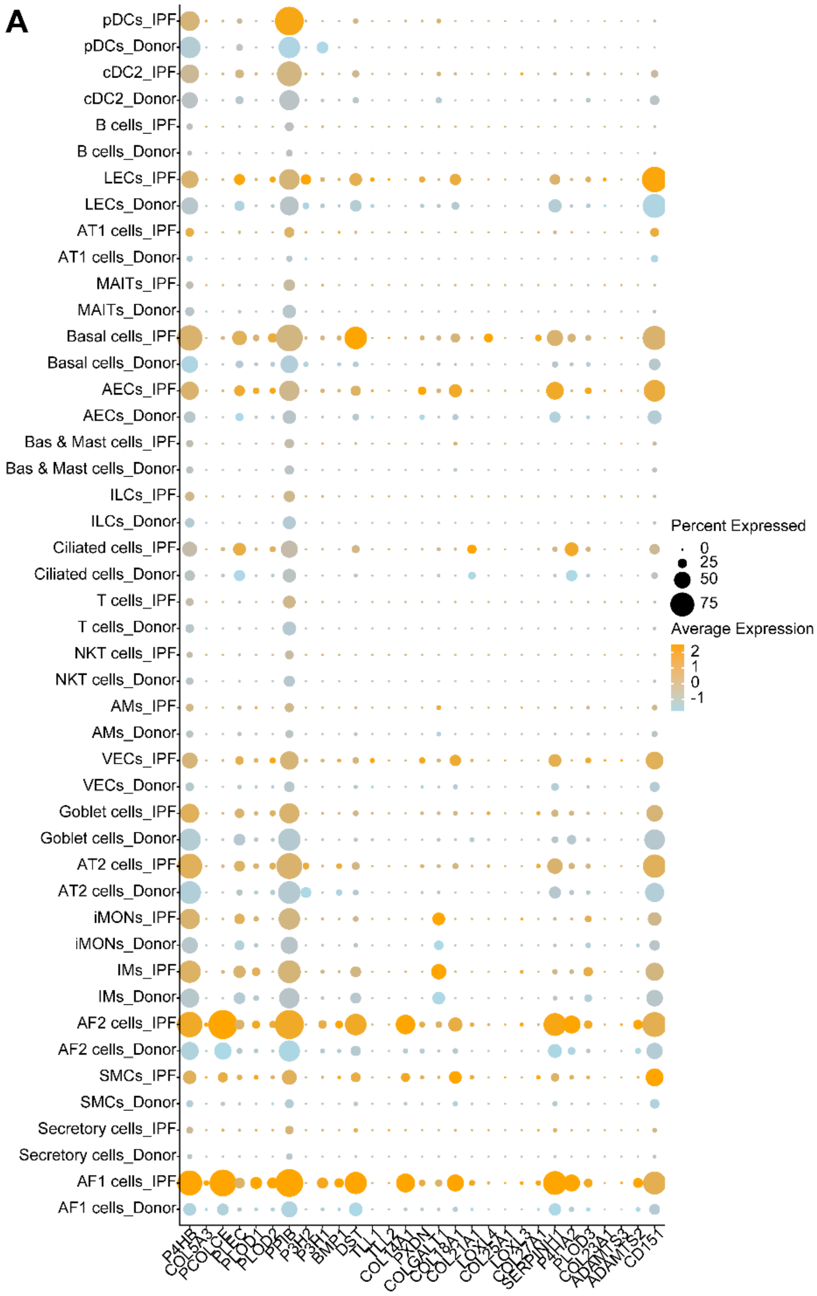

Figure S52. The expression levels of several genes involved in collagen formation are shown across 23 subsets in both HDs (n=10) and IPF patients (n=8).

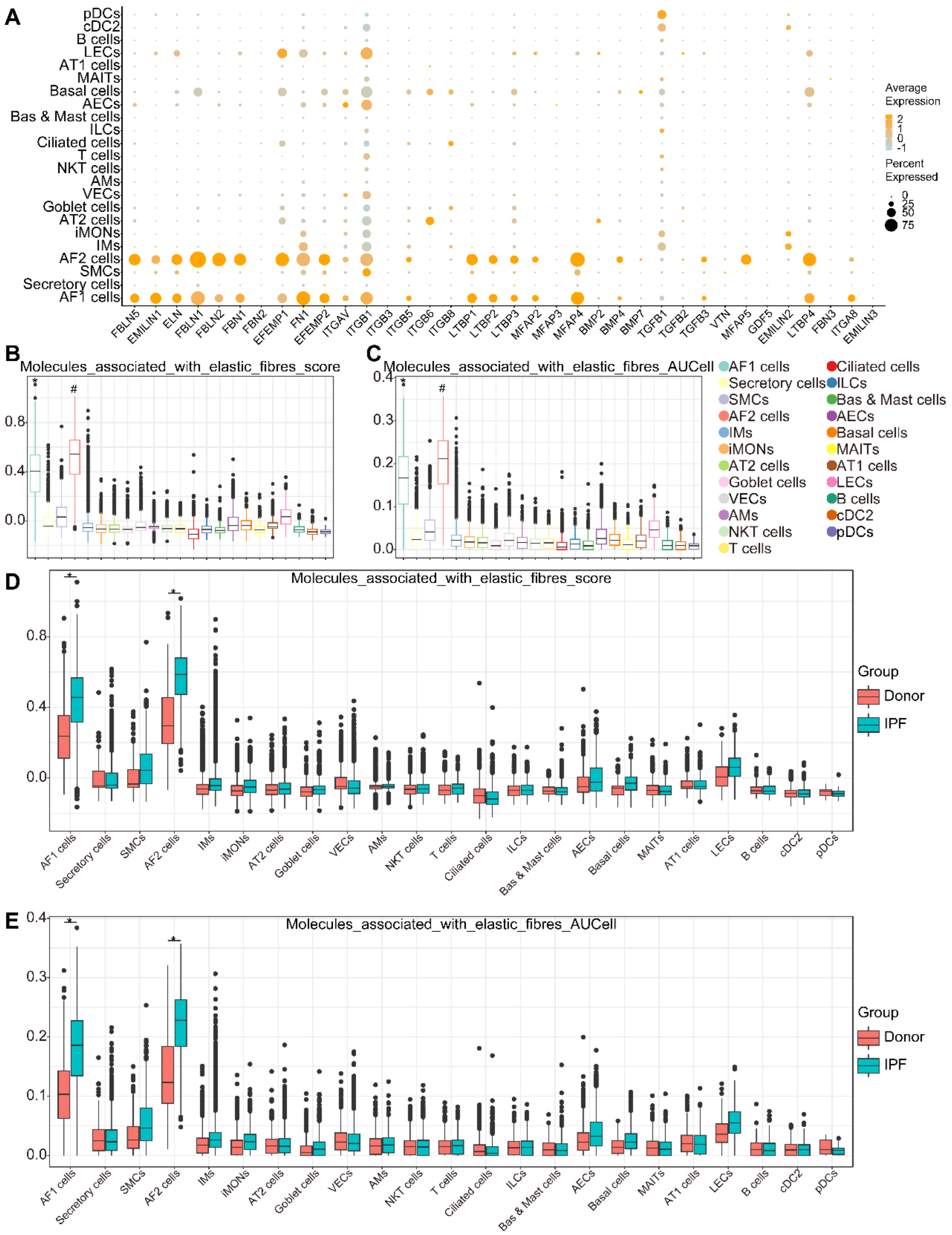

Figure S53. AF1 and AF2 cells exhibited significantly elevated activity of molecules associated with elastic fibres. (A) The expression levels of genes associated with the "Reactome: R-HSA-2129379 molecules associated with elastic fibres" are shown across 23 subsets. The activity score of molecules associated with elastic fibres is shown in each subset and was calculated via the AddModuleScore function (B) and the AUCell function (C). The activity score of molecules associated with elastic fibres is shown across 23 subsets for HDs (n=10) and IPF patients (n=8) and was calculated via the AddModuleScore function (D) and the AUCell function (E). (B, C) *, P < 0.05 for comparisons of AF1 cells with all other subgroups except AF2 cells; #, P < 0.05 for

comparisons of AF2 cells with all other subgroups except AF1 cells. P values were determined using (B and C) 1-way ANOVA with Tukey's post-hoc test or (D and E) 2-way ANOVA (*$P < 0.05$) with Tukey's post-hoc test. The data are presented as (B, C, D and E) the means ± SDs.

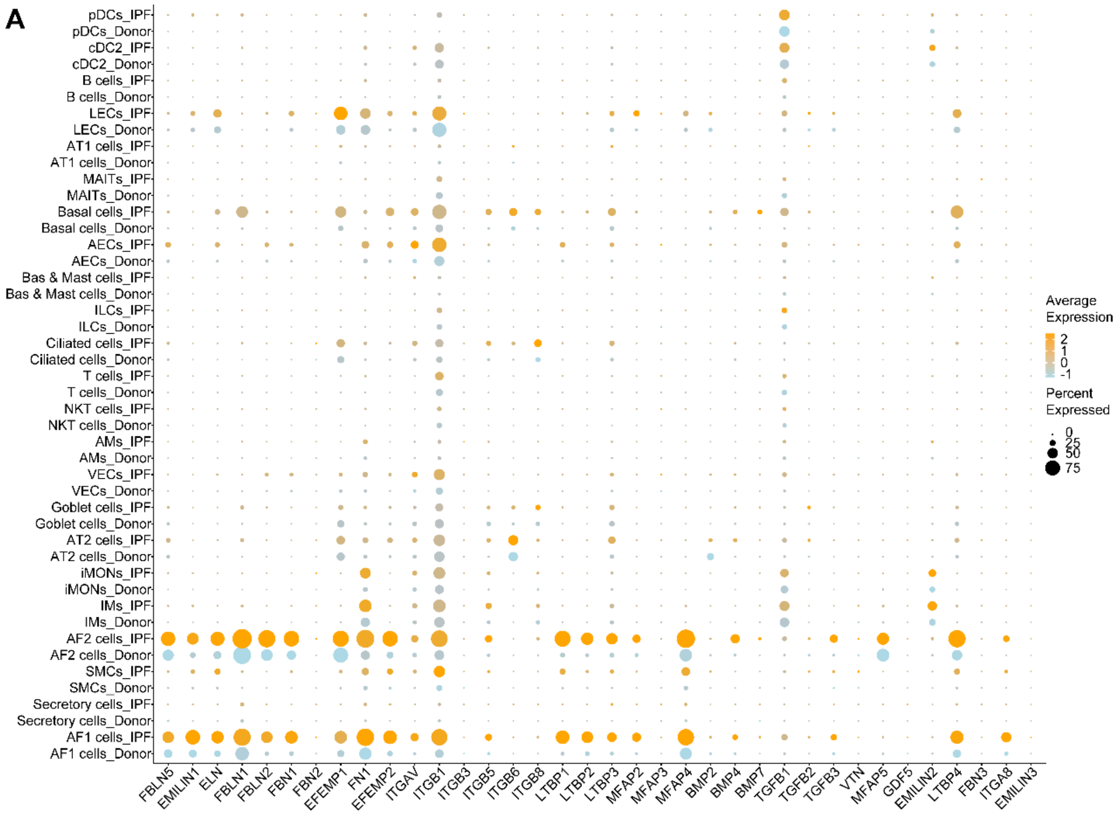

Figure S54. The expression levels of genes associated with elastic fibres are shown across 23 subsets in both HDs (n=10) and IPF patients (n=8).

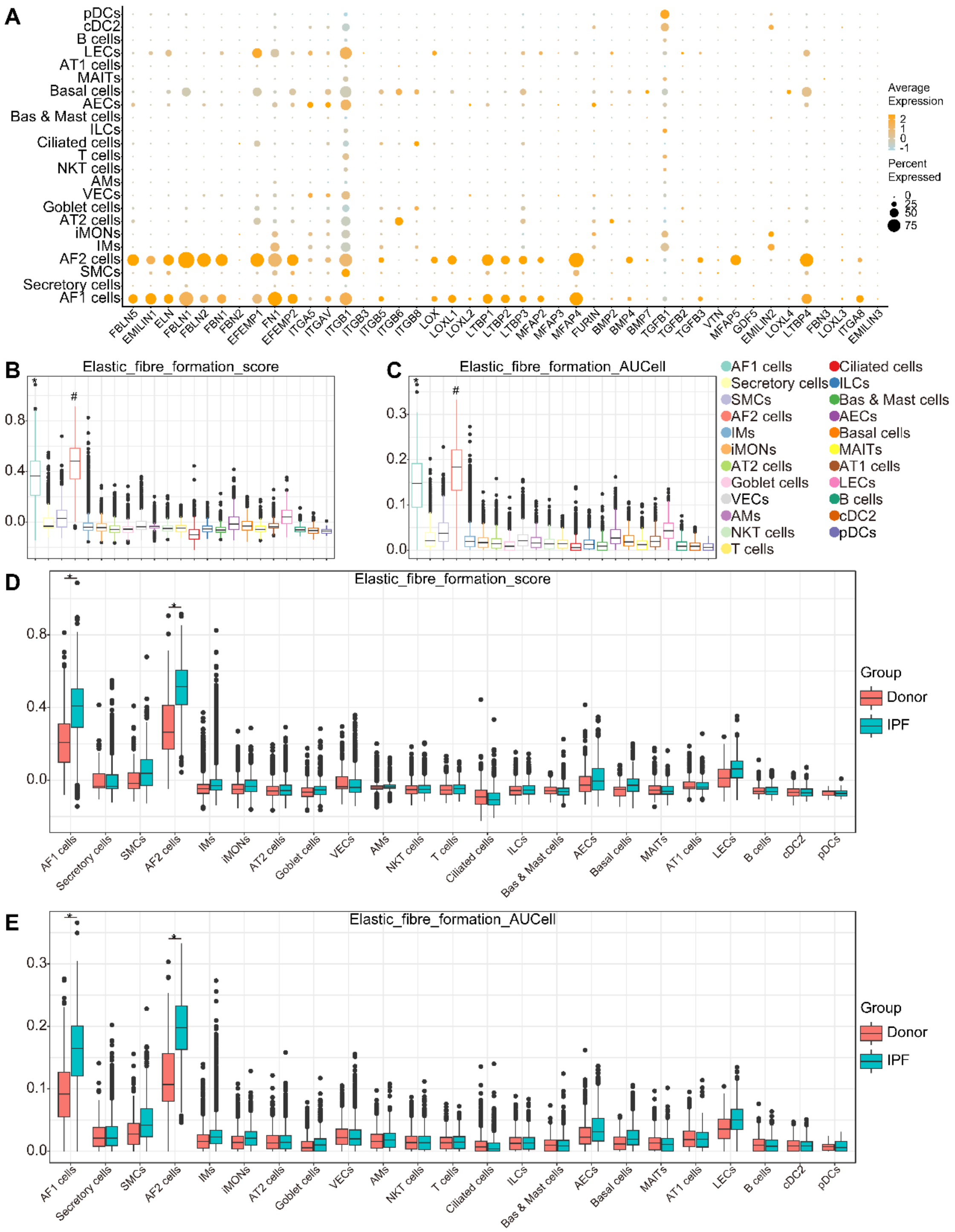

Figure S55. AF1 and AF2 cells exhibited significantly elevated elastic fibre formation activity. (A) The expression levels of genes involved in "Reactome: R-HSA-1566948 elastic fibre formation" are shown across 23 subsets. The activity score of elastic fibre formation is shown in each subset, which was calculated via the AddModuleScore function (B) and the AUCell function (C). The elastic fibre formation score of the 23 subsets is shown for HDs (n=10) and IPF patients (n=8) and was calculated via the AddModuleScore function (D) and the AUCell function (E). (B, C) *, $P < 0.05$ for comparisons of AF1 cells with all other subgroups except AF2 cells; #, $P < 0.05$ for comparisons of AF2 cells with all other subgroups except AF1 cells. P values were

determined using (B and C) 1-way ANOVA with Tukey's post-hoc test or (D and E) 2-way ANOVA (*$P < 0.05$) with Tukey's post-hoc test. The data are presented as (B, C, D and E) the means ± SDs.

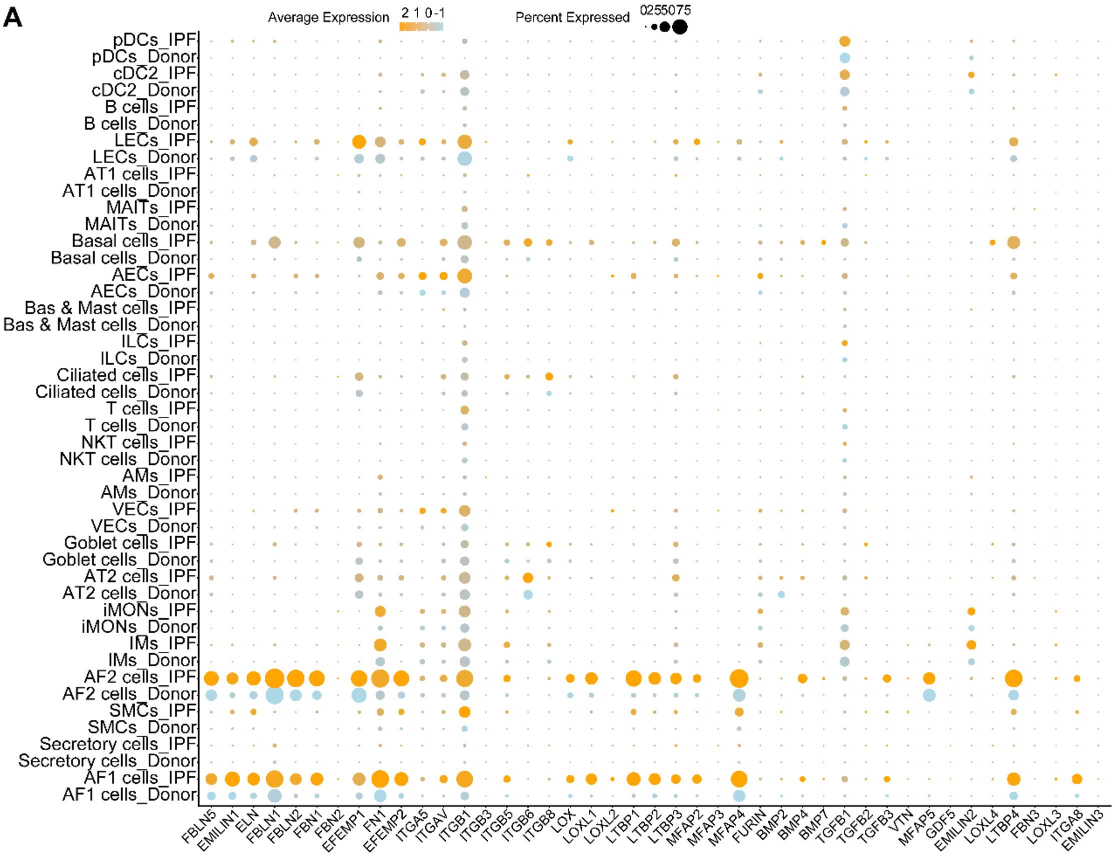

Figure S56. The expression levels of genes involved in elastic fibre formation are shown across 23 subsets in both HDs (n=10) and IPF patients (n=8).

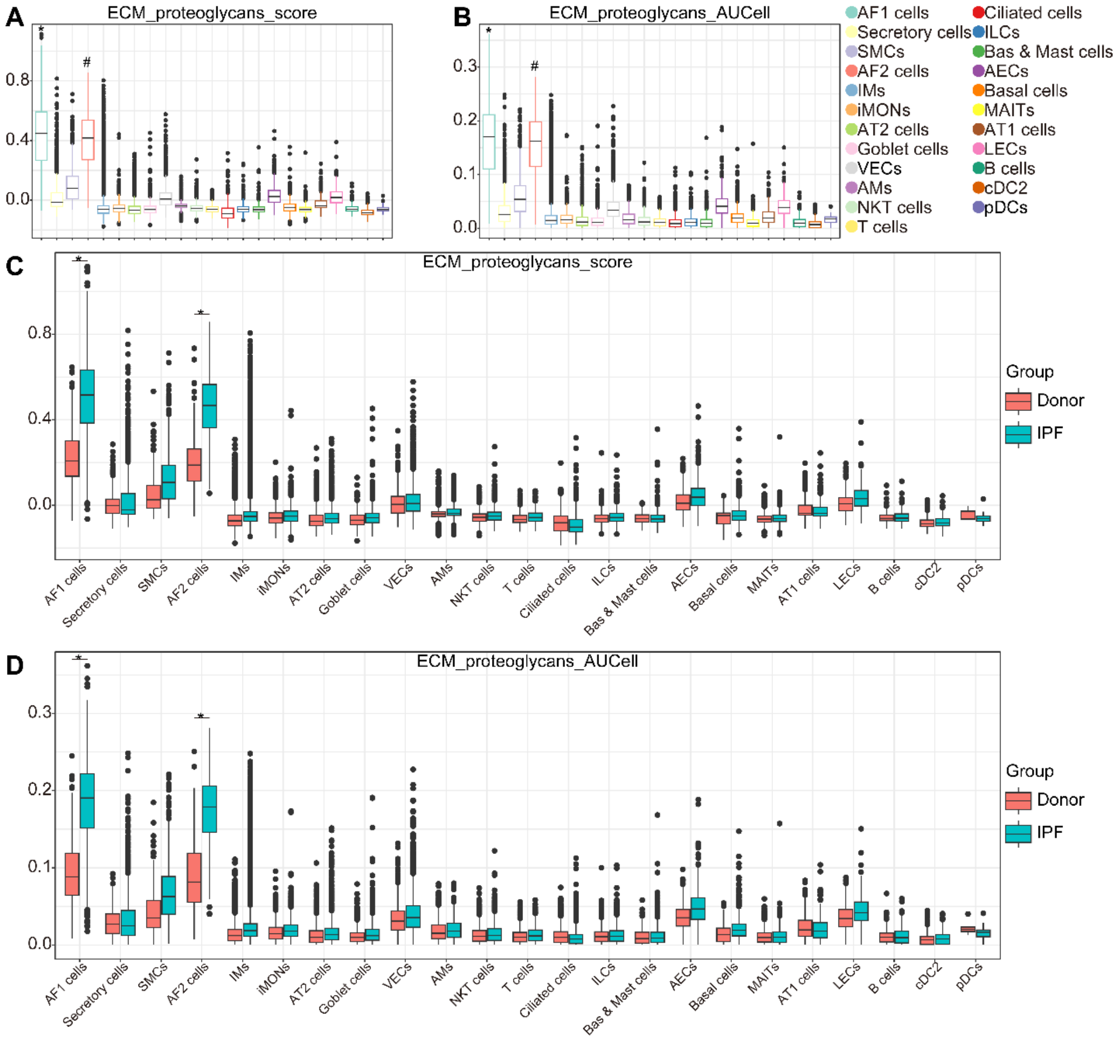

Figure S57. AF1 and AF2 cells presented significantly elevated ECM proteoglycans activity. The activity score of "Reactome: R-HSA-3000178 ECM proteoglycans" is shown for each subset and was calculated via the AddModuleScore function (A) and the AUCell function (B). The ECM proteoglycans score of the 23 subsets in HDs (n=10) and IPF patients (n=8) were calculated via the AddModuleScore function (C) and the AUCell function (D). (A, B) *, P < 0.05 for comparisons of AF1 cells with all other subgroups except AF2 cells; #, P < 0.05 for comparisons of AF2 cells with all other subgroups except AF1 cells. P values were determined using (A and B) 1-way ANOVA with Tukey's post-hoc test or (C and D) 2-way ANOVA (*P < 0.05) with Tukey's post-hoc test. The data are presented as (A, B, C and D) the means ± SDs.

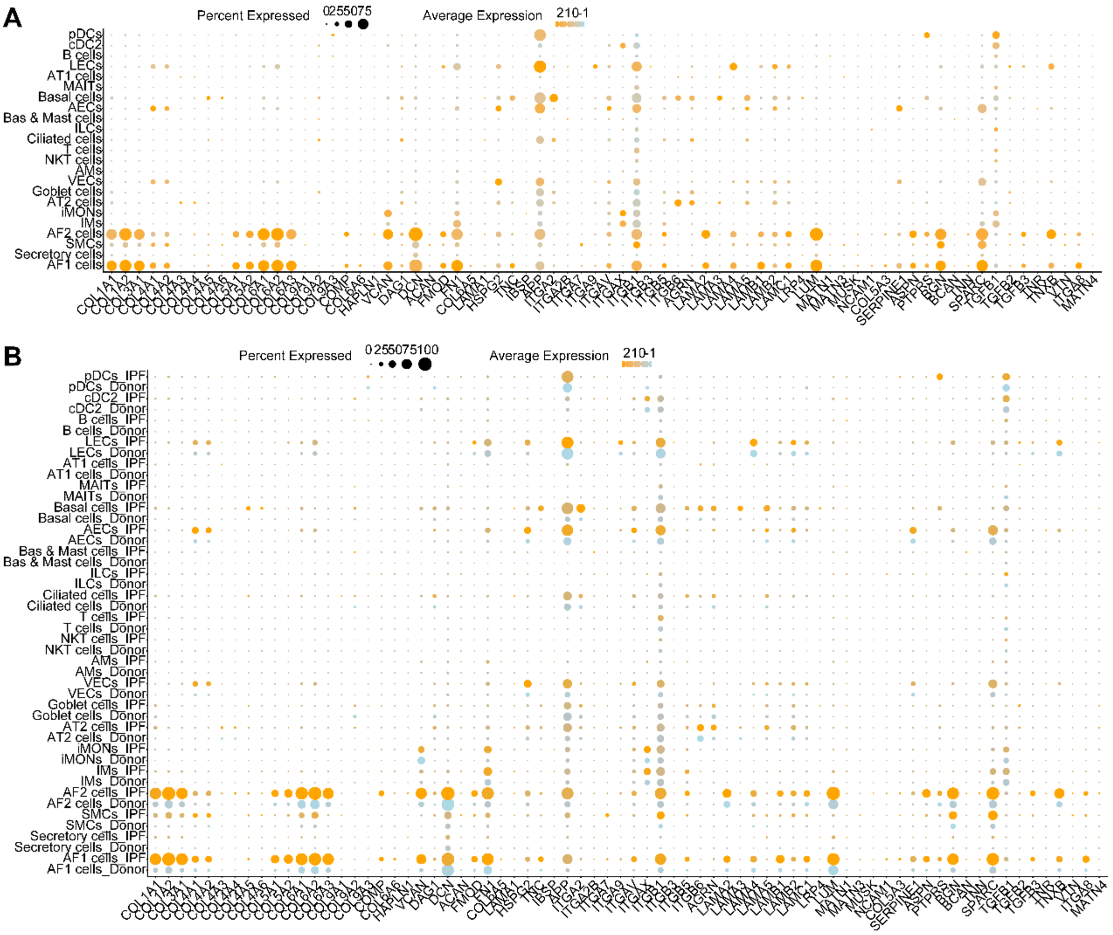

Figure S58. The expression levels of genes associated with ECM proteoglycans across 23 subsets. The expression levels of genes involved in "Reactome: R-HSA-3000178 ECM proteoglycans" are shown across 23 subsets (A) in both HDs (n=10) and IPF patients (n=8) (B).

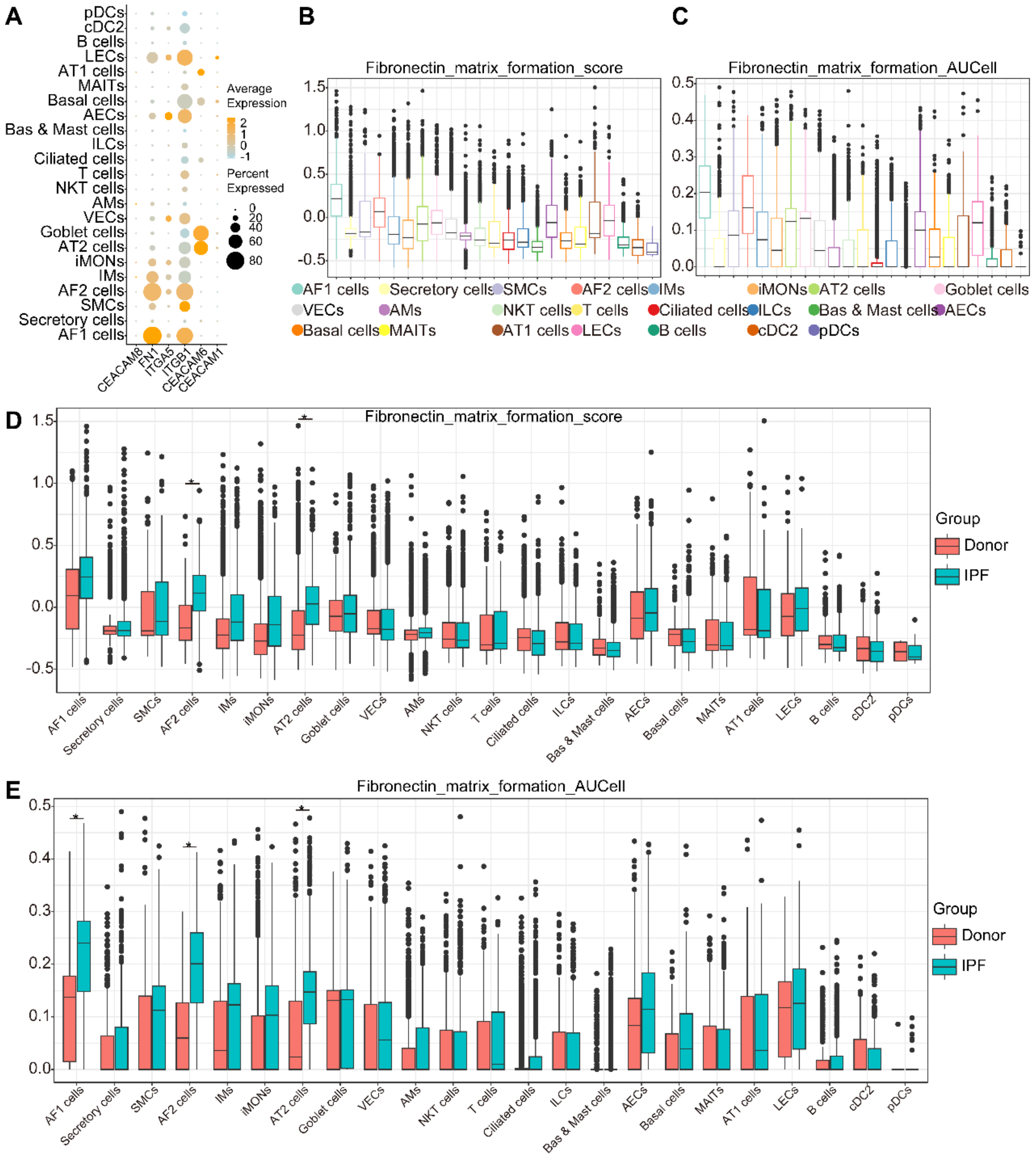

Figure S59. AF1 and AF2 cells exhibited significantly elevated fibronectin matrix formation activity. (A) The expression levels of genes involved in "Reactome: R-HSA-1566977 fibronectin matrix formation" are shown across 23 subsets. The activity score of fibronectin matrix formation is shown for each subset, which was calculated via the AddModuleScore function (B) and the AUCell function (C). The fibronectin matrix formation score of the 23 subsets is shown in HDs (n=10) and IPF patients (n=8) and was calculated via the AddModuleScore function (D) and the AUCell function (E). (B, C) *, P < 0.05 for comparisons of AF1 cells with all other subgroups except AF2 cells; #, P < 0.05 for comparisons of AF2 cells with all other subgroups except AF1 cells. P values were determined using (B and C) 1-way ANOVA with Tukey's post-hoc test or (D and E) 2-way ANOVA (*P < 0.05) with Tukey's post-hoc test. The data are presented as (B, C, D and E) the means ± SDs.

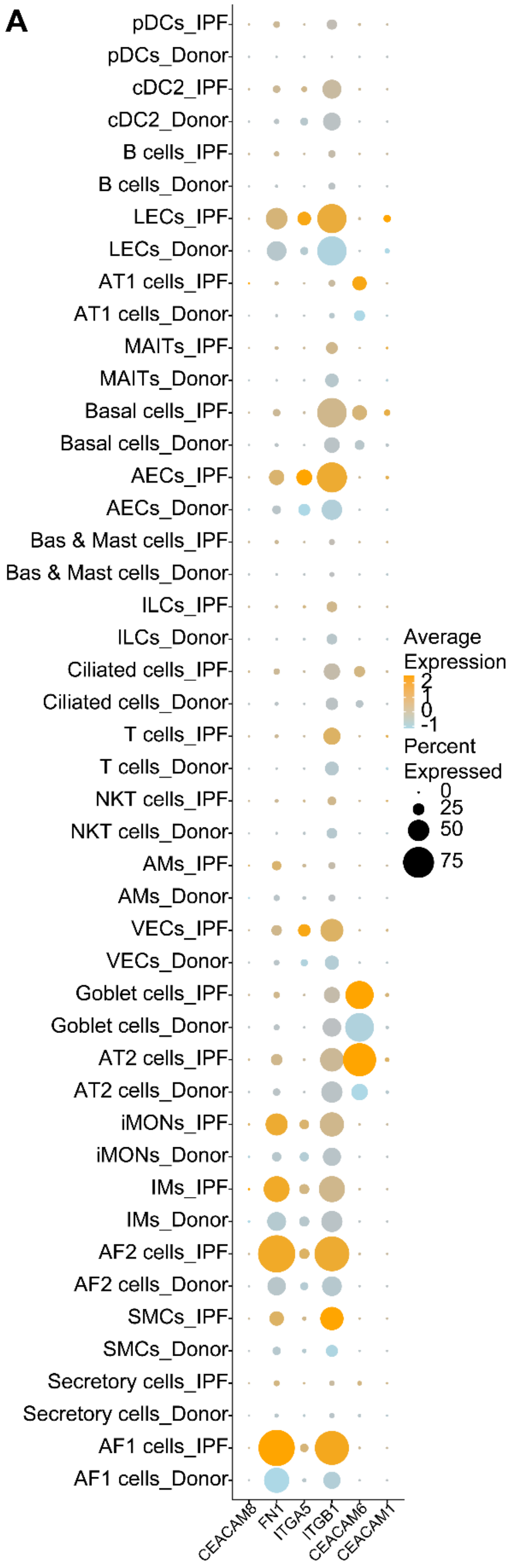

Figure S60. The expression levels of genes involved in fibronectin matrix formation are shown across 23 subsets in both HDs (n=10) and IPF patients (n=8).
